# Bacteria existing in pre-pollinated styles (silks) can defend the exposed male gamete fertilization channel of maize against an environmental *Fusarium* pathogen

**DOI:** 10.3389/fpls.2023.1292109

**Published:** 2023-12-04

**Authors:** Anuja Shrestha, Victor Limay-Rios, Dylan J. L. Brettingham, Manish N. Raizada

**Affiliations:** ^1^ Department of Plant Agriculture, University of Guelph, Guelph, ON, Canada; ^2^ Department of Plant Agriculture, University of Guelph, Ridgetown, ON, Canada

**Keywords:** maize, silk, style, microbiome, *Fusarium*, mycotoxin, Gibberella ear rot, biocontrol

## Abstract

In flowering plants, fertilization requires exposing maternal style channels to the external environment to capture pollen and transmit its resident sperm nuclei to eggs. This results in progeny seed. However, environmental fungal pathogens invade developing seeds through the style. We hypothesized that prior to environmental exposure, style tissue already possesses bacteria that can protect styles and seed from such pathogens. We further hypothesized that farmers have been inadvertently selecting immature styles over many generations to have such bacteria. We tested these hypotheses in maize, a wind-pollinated crop, which has unusually long styles (silks) that are invaded by the economically-important fungal pathogen *Fusarium graminearum* (*Fg*). Here, unpollinated silk-associated bacteria were cultured from a wild teosinte ancestor of maize and diverse maize landraces selected by indigenous farmers across the Americas, grown in a common Canadian field for one season. The bacteria were taxonomically classified using 16S rRNA sequencing. In total, 201 bacteria were cultured, spanning 29 genera, 63 species, and 62 unique OTUs, dominated by *Pseudomonas, Pantoea* and *Microbacterium*. These bacteria were tested for their ability to suppress *Fg in vitro* which identified 10 strains belonging to 6 species: *Rouxiella badensis, Pantoea ananatis, Pantoea dispersa, Pseudomonas koreensis, Rahnella aquatilis*, and *Ewingella americana.* Two anti-*Fg* strains were sprayed onto silks before/after *Fg* inoculation, resulting in ≤90% reductions in disease (Gibberella ear rot) and 70-100% reductions in associated mycotoxins (deoxynivalenol and zearalenone) in progeny seeds. These strains also protected progeny seeds post-harvest. Confocal fluorescent imaging showed that one silk bacterium (*Rouxiella* AS112) colonized susceptible entry points of *Fg* on living silks including stigmatic trichomes, wounds, and epidermal surfaces where they formed thick biofilms. Post-infection, AS112 was associated with masses of dead *Fg* hyphae. These results suggest that the maize style (silk) is endowed with potent bacteria from the mother plant to protect itself and progeny from *Fusarium*. The evidence suggests this trait may have been selected by specific indigenous peoples, though this interpretation requires further study.

## Introduction

In flowering plants, the fertilization process is initiated by maternal-style channels exposed to the external environment on which the pollen (male gametophyte) lands (Dresselhaus et al., 2011; Dresselhaus et al., 2016). The pollen then germinates on the stigma and elongates as pollen tubes encased within the style through which the sperm nuclei migrate to the ovule, resulting in fertilization and seed formation. However, the exposed style passage also acts as the entry point for environmental fungal pathogens that target seeds (Thompson and Raizada, 2018).

The maternal parent plays diverse roles in the reproduction process from guiding pollen tubes to ovules, to controlling seed development after fertilization (Yadegari and Drews, 2004; Drews and Koltunow, 2011). Similar to placental mammals (Chettoor et al., 2016), in flowering plants, an embryo develops inside the surrounding tissue of the mother, and its well-being depends on the protection and nurture provided by the maternal tissue, including against pathogens (Robert et al., 2018; Nguyen et al., 2021). Quantitative trait loci (QTLs) have been identified that improve style resistance to disease (Reid et al., 1996a; Reid et al., 1996b; Akohoue and Miedaner, 2022), suggestive that the maternal parent may protect the stigma-style passage, but little appears to be known about the underlying mechanism(s).

The style and stigma tissues have microbiomes. These microbiomes have only been explored in a few plants, including pear stigma (Stockwell et al.,1999), styles of the wild tree *Metrosideros polymorpha* (Junker and Keller, 2015), stigma and styles of the yellow monkeyflower (Rebolleda Gómez and Ashman, 2019), apple stigma (Cui et al., 2021), and maize styles with stigmatic hairs (Khalaf et al., 2021; Diniz et al., 2022a). In pear and apple, epiphytic bacteria from stigmata surfaces were reported to reduce the severity of fire blight disease caused by the bacterial pathogen *Erwinia amylovora* (Wilson and Lindow, 1992; Wilson et al., 1992; Johnson et al., 1993; Kearns, 1993; Lindow et al., 1996; Nuclo et al., 1998; Stockwell et al., 1999; Cui et al., 2021), suggesting that the microbiome of the maternal parent may contribute to its ability to protect the male migration route from environmental pathogens. However, to the best of our knowledge, in these studies, the flowers were open and hence the stigma/style was exposed to the environment, making it unclear if the microbes originated from the air and/or insects, or pollen/pollen tubes since pollen have rich, diverse microbiomes in comparison to other phyllosphere plant tissues (Obersteiner et al., 2016; Zasloff, 2017; Manirajan et al., 2018; Oteros et al., 2018; McFrederick and Rehan, 2019), or alternatively whether unpollinated styles have their own defensive microbiome of maternal origin.

Maize (*Zea mays* L.) is a wind-pollinated plant, requiring environmental exposure of the style to capture airborne pollen. These style channels are known as silks, the threads that extend outside of husk leaves at the tips of corn cobs, with each silk terminating in an ovule (Kroh et al., 1979; Heslop-Harrison et al., 1985). The environmental fungal pathogen, *Fusarium graminearum* (*Fg*) Schwabe, invades and grows through the silks at the time of pollination, resulting in a seed disease called Gibberella ear rot (GER) (Reid et al., 1992). Maize silks are also targets of foraging insects (Hazzard et al., 2003), which create entry (wound) sites for fungal pathogens including *Fg*. *Fg* and associated species then synthesize several mycotoxins, including deoxynivalenol (DON) and zearalenone (ZEA), which affect human and livestock health after being consumed, causing major economic losses to maize farmers globally (Miller et al., 2007; Thompson and Raizada, 2018). DON also suppresses plant defense responses (McCormick et al., 2011), thereby promoting *Fg* infection (Mudge et al., 2006). Critically, *Fusarium* species are also known to be present in pollen, and DON and ZEA have been shown to suppress pollen tube growth (Tejaswini, 2002; Kačániová et al., 2011; Kostić et al., 2019) which would prevent fertilization. Therefore, the silk-invading pathogen *Fg* and its mycotoxins reduce the fitness of future progeny and ultimately the genetic contribution of the female gametes. The long length, abundance, and accessibility of maize silks, combined with the economic importance of *Fg*, make it a model system for style-pathogen interactions.

A previous study from our group showed that maize silks have a complex microbiome: silks from a North American maize diversity panel were shown to host >1300 bacterial genera when combined (Khalaf et al., 2021). The study also demonstrated that some silk-associated bacterial taxa increased in relative abundance after silk *Fg* infection but they were not cultured and hence could not be tested functionally. One challenge is that the study employed short-length 16S rRNA sequencing (V4 region) (Khalaf et al., 2021) which has limited taxonomic resolution, and hence silk-associated bacterial species that may have anti-*Fg* activity could not be identified at the species level. Furthermore, the study focused on open-pollinated maize silks, and hence as already noted, the maternal contribution of these microbes was ambiguous.

Another recent study involving modern Brazilian maize hybrids (unidentified) reported dominant silk-associated bacteria belonging to the genera *Bacillus, Burkholderia, Achromobacter, Pseudomonas*, and *Serratia* (Diniz et al., 2022a). The silk-derived bacteria were shown to be antagonistic to the maize stem rot pathogen *Fusarium verticillioides* both *in vitro* and in greenhouse trials, also reducing fungal growth when applied to stored grains, but their activity on silks was not tested (Diniz et al., 2022b). Furthermore, in the Brazilian study, as in the above studies, the microbes were isolated from open-pollinated silks, making their parental origin unknown. In maize, the microbiota of unpollinated silks has not been reported to the best of our knowledge. However, endophytic microbes inhabiting other tissues in maize and its relatives (e.g. seeds, roots) have been shown to combat *Fg* when sprayed onto silks (Mousa et al., 2015).

The ability of the maternally derived microbiome to defend the male gamete migration route against environmental pathogens remains unclear. Any study must screen microbes from stigma-styles/silks that are not exposed to pollen (the male microbiome), pollinators, and/or the ambient environment, and critically, must show direct evidence that these microbes can protect stigma-style tissues (e.g. using microscopy). If this maternal ability exists, then there should be evidence that farmers have been able to select for such microbes in their crops.

Here, we hypothesized that in plants, the maternal parent can pro-actively protect the male gamete migration route (stigma-style channel) from environmental pathogens. Specifically, in maize, we hypothesized that unpollinated silks possess bacteria that can protect silks from *Fg* at the time of pollination. Furthermore, we hypothesized that indigenous farmers across the Americas have inadvertently selected immature silks to have such bacteria, either by selecting seed for re-planting that are symptomless (healthy) and/or which belonged to seed batches that did not make humans or livestock sick because they had low concentrations of mycotoxins – though the latter would be less likely.

To test these hypotheses, we cultured bacteria from unpollinated maize silks, protected from environmental exposure by husk leaves and ear bags, originating from maize landraces selected by indigenous peoples from across the Americas. Unpollinated silks were collected from plants grown in a common field in Canada for one season. The bacteria were taxonomically classified using full-length 16S rRNA sequencing, tested for anti-*Fg* activity *in vitro*, and then sprayed onto silks prior to deliberate *Fg* exposure followed by progeny seed disease/mycotoxin analysis, to verify their biological relevance. A further trial tested whether the silk-derived bacteria could protect progeny seeds from *Fg* even after they had dispersed from the mother plant. Finally, we used confocal fluorescence imaging of living silks for direct evidence of whether silk-derived bacteria could defend silks against *Fg*.

**Figure 1:**
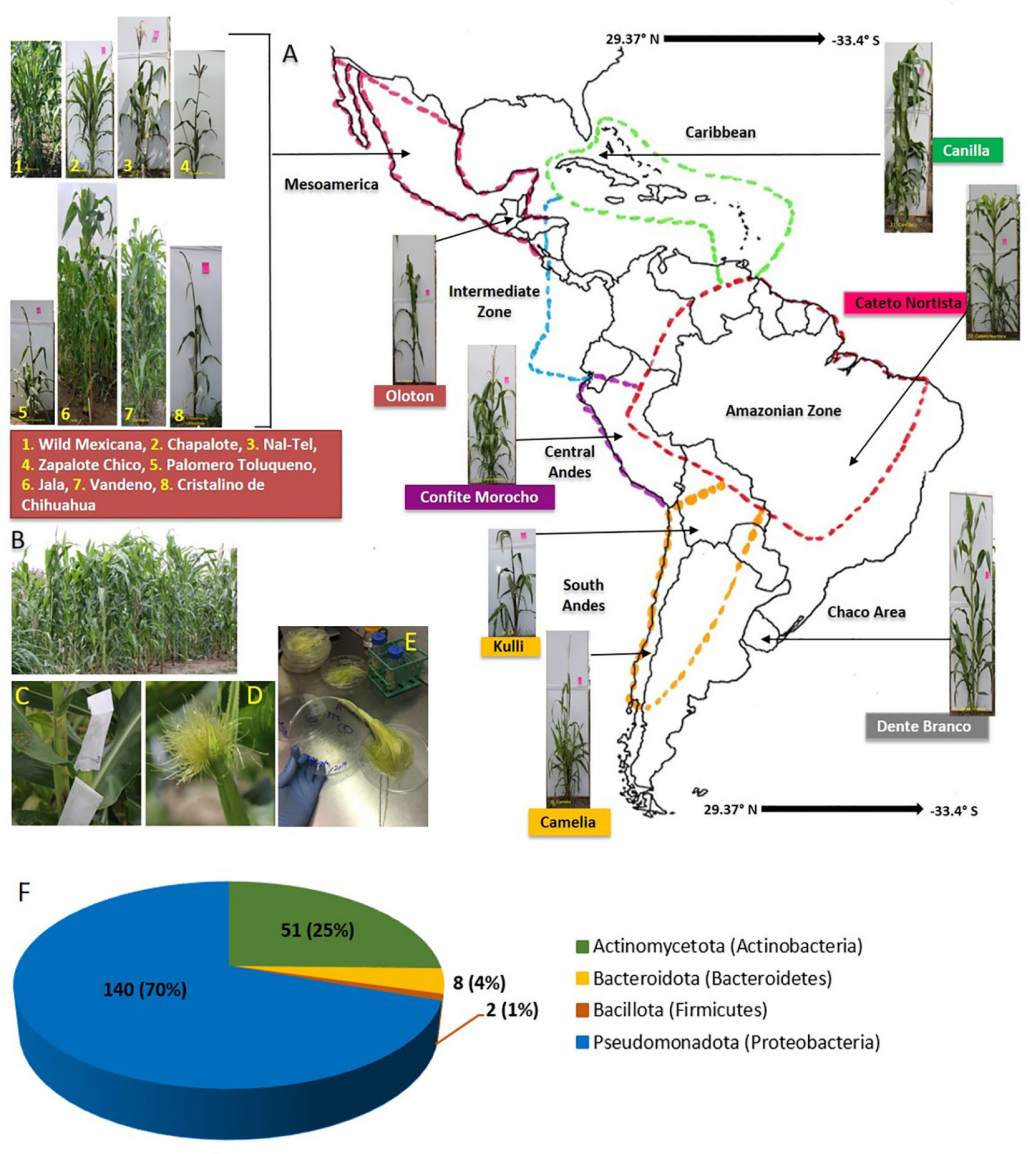
Origin of maize accessions used in this study to culture unpollinated silk-associated bacteria and their phylum level taxonomic distribution. Plants were grown in a common field in Canada. **(A)** A map of Latin America and the Caribbean showing the origin of the fifteen maize accessions used in this study. **(B)** Picture of the common maize field taken at the flowering stage, at Elora, Ontario, Canada. **(C)** Example of a maize ear covered within an ear bag to avoid silk exposure to the environment. **(D)** Example of silks ready for harvesting wherein silks had emerged inside the ear bags. **(E)** Processing of silk samples in the laboratory under sterile conditions prior to freezer storage in glycerol. **(F)** Phylum-level taxonomic distribution of the 201 bacterial isolates cultured in this study; the numbers on the left are the absolute number of isolates belonging to each phylum, while the numbers in brackets indicate their relative percentage in the silk-associated bacterial library.

Maize landraces as sources of silk-derived microbes were selected that spanned diverse geographies, cultivation timespans, and indigenous peoples (farmers) from across the Americas. Maize was domesticated about 9000 years ago in southwestern Mexico from wild teosintes including *Zea mays* ssp. *mexicana*, the minor teosinte ancestor of modern maize, which has contributed 12% of modern maize alleles (Matsuoka et al., 2002). After being domesticated, ancient maize was migrated across the Americas by distinct indigenous groups (**Figure 1**; **Table 1**) (Matsuoka et al., 2002; Warburton et al., 2011; Prasanna, 2012; Bedoya et al., 2017). In each new environment, farmers independently selected maize landraces to adapt to the local environment and needs (Ruiz Corral et al., 2008; Vigouroux et al., 2008; Langner et al., 2019).

**Table 1:** Information about the origin of maize accessions selected in this study.

*Zea* accessions	Category	Seed Origin	Latitude	Longitude	Rainfall^^^	Altitude*	Accession ID
Cristalino de Chihuahua	Landrace	Mexico	29.37	-107.73	Dry	Highland	CIMMYTMA 7961^2^
Chapalote	Landrace, Primitive	Mexico	24.86	-107.42	Moderate	Lowland	NSL 2833^1^
Jala	Landrace	Mexico	21.09	-104.43	Moderate	Mid-altitude	CIMMYTMA 2246^2^
Canilla	Landrace	Cuba	20.9	-76.25	Moderate	Lowland	CIMMYTMA 5393^2^
Nal-Tel	Landrace, Primitive	Mexico	20.25	-89.65	Dry	Lowland	CIMMYTMA 2357^2^
Palomero Toluqueno	Landrace, Primitive	Mexico	19.283	-99.667	Wet	Highland	CIMMYTMA 6756^2^
*Zea mays* ssp. *mexicana*	Minor wild ancestor	Mexico	19.16	-98.55	Moderate	Highland	PI 566680^1^
Vandeno	Landrace	Mexico	17.52	-101.28	Moderate	Mid-altitude	CIMMYTMA 177^2^
Zapalote Chico	Landrace	Mexico	16.217	-93.889	Moderate	Lowland	CIMMYTMA 10473^2^
Oloton	Landrace	Guatemala	14.633	-90.517	Moderate	Highland	CIMMYTMA 2510^2^
Confite Morocho	Landrace, Primitive	Peru	-12.77	-75.03	Wet	Highland	CIMMYTMA 8381^2^
Cateto Nortista	Landrace	Brazil	-16.667	-49.255	Wet	Mid-altitude	CIMMYTMA 26373^2^
Kulli	Landrace, Primitive	Bolivia	-18.18	-65	Moderate	Highland	CIMMYTMA 14235^2^
Dente Branco	Landrace	Uruguay	-32.683	-58.133	Wet	Lowland	CIMMYTMA 6162^2^
Camelia	Landrace, Primitive	Chile	-33.45	-70.667	Dry	Lowland	CIMMYTMA 15218^2^

^1^ Denotes seeds obtained from U.S. Department of Agriculture (Germplasm Resources Information Network, GRIN) and ^2^ from CIMMYT, Mexico.

*Lowland = 0-700 m.a.s.l; Mid-altitude = 701-1600 m.a.s.l; Highland = >1601 m.a.s.l.

^Rainfall classification: Dry= 500 - <800 mm; Moderate=>800 - <1270 mm; and Wet= >1270 mm.

Additional information is in **Supplementary Table 1**.

Near the center of maize diversification in south-central Mexico, ancient indigenous peoples selected the highland Mexican popcorn landrace, Palomero Toluqueno (Rocío Aguilar-Rangel, 2018); it is reported to be resistant to drought and pests (Arnason et al., 1994), and is a progenitor of many productive modern highland landraces (Perez-Limó et al., 2022).

From its center of diversification, maize was then migrated north and south. Moving northwards, the ancient landrace Jala became known for its giant plant height (up to 5 m tall); it bears the longest cobs in the world and hence extremely long silks (Rice, 2007). Also in northern Mexico, the primitive lowland popcorn landrace Chapalote became highly valued by traditional farmers and has been continuously grown for 3000 years (Bonavia, 2013); Chapalote is thought to be the first maize to enter the United States more than 2000 years ago (Da Fonseca et al., 2015). Cristalino de Chihuahua, a more recent northern Mexican landrace, was adapted to very dry conditions (Ruiz Corral et al., 2008).

On the southern migration route of maize, the ancient Mayans of the Yucatan Peninsula in southern Mexico grew the landrace Nal-Tel, a distinctive primitive popcorn (Turrent and Serratos, 2004) that was critical to their diet (Santillán-Fernández et al., 2021). Similarly, Zapalote Chico was adapted to southern Mexican/Central American environments and later contributed to food security across Latin America (Taba et al., 1995). Also in this region, Vandeno became an agronomically important landrace that was the progenitor of many modern maize varieties (Bedoya et al., 2017) and has been extensively used in breeding programs (Taba, 1995); it is resistant to aflatoxin produced by the fungus, *Aspergillus flavus* (Ortega-Beltran et al., 2014). In Guatemala, indigenous peoples selected the highland landrace Oloton which was later introduced to Mexico (Wellhausen, 1952), becoming a staple food for the Mixe indigenous people in the Sierra Mixe region of Oaxaca in southern Mexico (Pskowski, 2019); it is highly resistant to pests and pathogens (CONABIO, 2017) and has aerial roots that produce a mucous-like gel that fixes nitrogen (Pskowski, 2019). In South America, Confite Morocho from Peru became the primitive ancestor of Andean popcorn (Goodman and Brown, 1988), known for its short stature and small ears, and hence short silks (Grobman et al., 1961). Kulli became a Bolivian highland maize landrace (Cuevas Montilla et al., 2011), popular for making fermented beer (Ricardo et al., 1960). Cateto Nortista, a tropical flint, was selected as late-flowering maize in coastal Brazil (Bedoya et al., 2017), while Camelia was selected in lowland Chile to be resistant to Fusarium ear rot (USDA-ARS, 2018). Dente Branco was reportedly migrated from the United States to lowland Uruguay and possesses resistance to Fusarium ear rot (USDA-ARS, 2018). Maize was also migrated to the Caribbean, where the landrace Canilla was adapted to the lowland region of Cuba (Hatheway, 1957).

Here we provide multiple lines of evidence in maize, including from direct microscopy, to demonstrate that while sexual reproduction necessitates that silks become exposed to the environmental pathogen *Fg* to enable capture and transmission of male gametes, some maternally derived silks possess bacteria that can protect silks and progeny seed from *Fg*. Furthermore, we provide preliminary evidence that suggests that distinct indigenous peoples in the Americas have selected for such defensive bacteria, but further confirmation is required. Since there are no prior studies on bacteria isolated from unpollinated maize silks, we also report preliminary qualitative observations on taxonomic patterns within the culturable maize silk microbiome across maize accessions, with the limitation that the bacteria were cultured from plants grown in a common field and a single season.

## Materials and methods

### Maize accession selection, growth conditions, and experimental design

A diverse panel of fifteen maize accessions including wild maize and ancient maize landraces from the Americas were selected for the study (**Figure 1**); the detailed information of which is presented in **Tables 1** and **S1**. Seeds of 2 maize accessions (Chapalote and *Zea mays* ssp. *mexicana*) were received from the U.S. Department of Agriculture (Germplasm Resources Information Network, GRIN), and the remaining 13 from the Maize Germplasm Bank of the International Maize and Wheat Improvement Center (CIMMYT), Mexico. These maize accessions required a short day length between V5 to V8 growth stages to subsequently induce flowering in Ontario’s long-day field conditions since they were short-day adapted maize from the tropical regions, and were sensitive to photoperiod. To allow this, the seeds were first sown in 5X5 inch biodegradable pots filled with a mixture of Sunshine Mix (LA4, Sungrow®Horticulture, Brantford, Ontario, Canada) and field soil from the Elora Research Station, Elora, Ontario, Canada. Field soil was added to provide a source of microbes since some endophytes are known to be taken up through the roots. The pots were arranged in trays by maize accession and were grown in a growth room under a controlled environment in the University of Guelph Crop Science growth room facility with a 14-hour photoperiod until the seedlings reached the V5 growth stage. Then, the photoperiod was reduced to 10 hours until the V8 growth stage. Manual irrigation as well as fertilization with 20:20:20 plus micronutrients (Plant-Prod 20-20-20 Classic, Product Number: 10529, Brampton, Ontario, Canada) was done. The photoperiod for the plants was supplied with fluorescent light (LED 18 ET9/4/850 bulbs, GE), supplemented with LED 9.5 A19/DIM/0/827/G4 1100 Lumen 2700K bulbs (OSRAM, Canada). To supply equal light distribution to the plants, the trays were rotated twice per week, where the light intensity was 425-515 μmol m-2 s-1 at pot level. At the V8 growth stage, a total of 780 large corn seedlings were loaded in a trailer and transported to the field at Elora Research Station (latitude: 43°41’ 3.59” N; longitude: -80° 25’ 22.79” W). The seedlings were acclimatized to the field environment by keeping them under direct sunlight for 2-3 hours/day and left under the shade for the remaining time. This was important to avoid sunburn problems and seedling death since they originated from a controlled environment inside the growth room. After 3 days, the seedlings were transplanted to the field on the 10^th^ of July, 2019, in a randomized block design with five replicate blocks, each having six plants. The field was pre-applied with 160 kg/ha of N, 60 kg/ha of P_2_O_5_, 80 kg/ha of K_2_O, 10 kg/ha of S fertilizers and herbicides (4.0 l/ha of Primextra, and 0.3 l/ha of Callisto). Irrigation of the seedlings was done manually for one week after transplantation.

### Silk harvesting, culturing and taxonomic analyses

Silk harvesting was undertaken from the 3^rd^ week of August to the 2^nd^ week of October 2019. The ears were bagged before the silks emerged; they were allowed to develop and then harvested at the first emergence. The harvested ears were brought back to the lab with the ear bags on, then under sterilized conditions, ~ 3 cm at the cob tips were cut and removed, the remaining cob was de-husked, and their silks were dissected and kept for culturing. Each silk sample was pooled from all five field blocks, with 3-5 ears per block. Thereafter, 40% sterile glycerol was added to the pooled samples which were then stored at -80°C. The frozen silk samples were ground and the lysates were used to culture microbes on Reasoner’s 2A (R2A) (R2A powder 18.12 g/L – containing 0.5 g yeast extract, 0.5 g proteose peptone, 0.5 g casein hydrolysate, 0.5 g glucose, 0.5 g starch, 0.3 g di-potassium phosphate, 0.024 g magnesium sulphate, 0.3 g sodium pyruvate, and 15 g agar, per liter, adjusted to pH 7.2 with NaOH and autoclaved) and LB media (Luria-Bertani, composed of 10 g NaCl, 5 g yeast extract, and 10 g tryptone, per liter, adjusted to pH 7.2), followed by 16S rRNA based taxonomic profiling using 27F and 1492R primers, and phylogenetic tree construction using MEGA-X software. For details of culturing, glycerol stock preparation, taxonomic analysis, OTU assignment and phylogenetic tree construction, see **Supplemental Methods**.

### *In vitro* assays for anti-*Fusarium* screening

Dual culture assays were undertaken to test silk-associated bacteria *in vitro* for anti-*Fg* activity, by modifying an earlier method (Mousa et al., 2015). See **Supplemental Methods** for details. For analyzing the results of these assays, the diameter of each *Fg*-inhibition zone was statistically modeled with a generalized linear mixed model (GLMM) using PROC GLIMMIX, then analyzed using One-Way ANOVA, and compared the means using Tukey’s pairwise comparison in SAS 9.4 (SAS Institute, Cary, NC) with a significance level of P ≤ 0.05. However, while reporting here, each silk-associated anti-*Fg* bacteria was compared only with the negative control (LB Control) and the positive control (Proline, the commercial fungicide) treatments.

### Greenhouse trials to test the ability of silk-associated bacterial strains to suppress Gibberella ear rot disease

#### Microbial treatment selection: antibiotic susceptibility testing

Candidate anti-*Fg* bacteria were tested for susceptibility to 20 clinical antibiotics to assure human safety, and only the safest strains proceeded to greenhouse trials (see **Supplemental Methods** for details).

#### Plant growth conditions and experimental design

Seeds (surface-sterilized) belonging to a moderately susceptible commercial maize hybrid DKC55-05RIB (Bayer Crop Science, Canada) were grown in the University of Guelph Crop Science Greenhouse Facility, starting May 25, 2021 (Trial 1) and June 11, 2021 (Trial 2) as described (See **Supplemental Methods** for details). In total, there were 168 maize pots organized as a randomized block design. There were seven treatments (two silk-associated bacteria, a negative and positive control, along with three non-silk-associated bacteria to be reported in a separate manuscript). The treatments were arranged into 6 blocks, each having 4 treatment replicates. One replicate was defined as one plant in one pot. A second completely independent trial was initiated two weeks after the first trial in a separate greenhouse. Only the upper (primary) ear from each plant was pollinated, treated, and used for disease scoring.

#### Bacterial and control treatments

Each bacterial strain and the negative control treatment were sprayed onto silks twice to ensure a high titer. The first spray was done at 48 h after pollination followed by waiting for 72 h to ensure effective colonization of bacteria. The bacterial strains were grown in LB broth (pH 7.2) for 48 h at 30°C with shaking at 200 rpm, then centrifuged for 10 min, resuspended to OD_600_ of 0.4-0.6 and then 1 mL was sprayed onto each ear using spray bottles. To avoid any cross-contamination of the sprays, the air coolers in each greenhouse zone were turned off each day during and after spraying (from 4:30 pm to 6:00 am). The second bacterial inoculation (1 mL/ear) was done 48 h following the pathogen application and prepared as already noted. The negative control included spraying with 1 mL of LB liquid (pH 7.2) (as a buffer control) similar to the bacterial treatment mentioned above. The positive control was a commercial fungicide, Prothioconazole (PROLINE 480 SC, Bayer Crop Science, Canada), mixed with 1.125% V/V of the surfactant Agral 90 (registration #11809, Syngenta Canada, Guelph, ON), and sprayed only once (1 mL/ear) onto the silks at 48 h after pollination, similar to the other bacterial treatments.

#### Pathogen (*Fg*) treatment

The *Fg* spore broth was prepared as follows: 0.35 g KNO_3_, 0.35 g KH_2_PO_4_, 0.175 g MgSO_4_, 0.175 g KCL, and 0.175 g dextrose were added to 175 mL of distilled water in a 1 L Erlenmeyer flask. Then, 175 µL micronutrient solution (20 mg/100 mL of each minor element: FeCl_2_, MnSO_4_, ZnSO_4_) was added to this flask and sterilized at 121°C for 20 min. Two PDA plugs of *Fg* isolate (the same isolate used for the dual culture assays) were added to the flask once the spore broth cooled down, covered with aluminum foil, and incubated in a dark incubator at 25°C with shaking at 120 rpm for 2 weeks. This spore broth was first filtered with cheesecloth and stored in the fridge, which was then standardized to 20,000 spores/mL with a haemocytometer on the day of pathogen inoculation. One mL of this standardized solution was sprayed onto the silks of any ears that were marked as pollinated, 72 h after the first bacterial treatment. The ears were then covered and tied up with white plastic bags to preserve humidity. These plastic bags were removed after 10 days, and water misting was done every afternoon for the next two weeks to keep each treated ear moist. Automated misting was set in the greenhouse zone (2 min/h) to keep the zone humid.

#### Harvesting of ears

Once the ears had matured, watering was ceased, approximately 45 days after the last pollination/crosses, and ears were let to dry for an additional week before harvesting. Criteria such as hardening of the cobs, size of the kernels, and the color of the cobs (deep yellow/orange) were checked to verify the maturity of the cobs. All the treated primary ears were harvested with husks on, packed in labeled bags with their pot identification number, and stored in cardboard boxes inside the greenhouse zone before their disease assessments.

#### Visual disease scoring of cobs

The disease scoring was done by visually phenotyping each harvested cob for the percentage of apparent infection, which was scored as the length of the disease area from the infection-starting site on the cob (cob tip) relative to the total length of each respective cob. The husks of the ears were removed before scoring. The measurements were taken from four different sides of each cob, and then the average of the infected portion was calculated relative to the total cob length. The cobs were then shelled and grain weight was taken from all the harvested cobs. All the scoring was done by the researcher blindly by selecting the bags with ears randomly. Once the scoring was complete for both greenhouse zones, then the results were organized by treatment and block based on the greenhouse treatment map. The percent cob-infected data was statistically modeled using PROC GLIMMIX in GLMM, analyzed using a beta distribution, and compared using Tukey’s pairwise comparison. The average grain weight data were analyzed using One-Way ANOVA and compared using Tukey’s pairwise comparison, both in SAS version 9.4 (SAS Institute, Cary, NC) with a significance level of P ≤ 0.05.

#### Visual assessment of kernel disease severity

In addition to visual disease scoring of cobs, a visual disease severity assessment of kernels from each cob was performed. Kernel disease scoring was done blindly by a co-author (D.B.), by visualizing one side/angle of the cob, i.e., half the side of each cob using pictures of cobs. The scoring was based on a 1-4 scale, with 4 being the most infected kernel (infected from all sides); 3 described as a medium infected kernel [(limited damage to the kernel on the top portion + damage on the sides of the kernel (e.g., covered with mycelia)]; 2 as a less infected kernel (healthy kernel on the top portion + damage on the sides of the kernel, or limited damage on the top portion + no damage on the sides of the kernel); and 1 noted as no kernel damage + not covered with *Fg*-mycelium. The individual kernel disease severity percentage data was statistically modeled with a GLMM using PROC GLIMMIX, and analyzed using One-Way ANOVA where the percentage means were compared using Tukey’s pairwise comparisons in SAS version 9.4 (SAS Institute, Cary, NC) with a significance level of P ≤ 0.05.

#### Quantification of deoxynivalenol and other associated mycotoxins

After the grain weights were taken, the seeds were pooled from all four cobs of each treatment per block, mixed, and then one-third of the mixture was sent for multi-toxin analyses. In total, there were 84 samples (6 replicate samples per treatment, 7 treatments/zone, and 2 greenhouse zones). Analyses of DON and other associated mycotoxins were conducted using liquid chromatography-tandem mass spectrometry (LC-MS/MS) method following a previously reported protocol (Limay-Rios and Schaafsma, 2021). The data were statistically modeled with a GLMM using PROC GLIMMIX, then analyzed using One-Way ANOVA Dunnett’s comparisons in SAS version 9.4 (SAS Institute, Cary, NC) with a significance level of (P ≤ 0.05).

### Light microscopy to visualize *in vitro* interactions between *Fg* pathogen and silk-associated bacterial strains

Light microscopy was used to study the *in vitro* interactions between *Fg* and the bacterial strains. For this, microscope slides were coated with a thin layer of 1 mL Potato Dextrose Agar (PDA) and allowed to solidify. 50 µL of *Fg* mycelia, grown for 72 h in Potato Dextrose Broth (PDB) at 25°C at 120 rpm, was applied in the center of the slide. On the adjacent side of this, 50 µL of each bacterial strain, cultured in LB liquid (pH 7.2) by incubating at 30°C at 200 rpm for 48 h, were applied. Likewise, LB broth was placed on the left side as a negative control. These slides were placed in closed Petri dishes and incubated at 25°C for 24 h. The positive control included Proline fungicide (1:10 v/v with ddH_2_O). The slides were then stained with the vitality stain, Evans blue (Catalog # E2129, Sigma Aldrich, Canada) by placing 1 mL of stain on the slide, which was then incubated for 5 min at room temperature, and washed 3-4 times with deionized water. Slides were prepared in triplicate for each treatment, visualized under a light microscope and pictures were captured.

### Bacterial fluorescent tagging and confocal scanning fluorescence microscopy

Strain AS112 competent cells were transformed with plasmid pSW002-PpsbA-DsRed-Express2 then selected on LB agar (pH 7.2) with 5 mg/mL tetracycline, incubated at 30°C for 24 h and screened for fluorescence (see **Supplemental Methods** for details). Seeds belonging to modern maize inbreds (PHRE1, LH82) were surface disinfected, germinated in the lab for 7 days, followed by transplanting into pots filled with 100% Turface® clay at the University of Guelph Crop Science Greenhouse Facility. In the laboratory, a previous protocol from our lab was followed (Thompson and Raizada, 2023). Briefly, DsRed-tagged AS112 culture was inoculated onto silks at the tip of each cob; subsequently, 24 h later, GFP-tagged *Fg* [strain ZTE-2A (Miller et al., 2004)] was inoculated. To mimic damage from insects, silks were wounded. Cobs were placed in darkness for 48 h at 25°C, then the silks were stained with propidium iodide solution (1 mg/mL) (Catalog# P4864-10ML, Sigma-Aldrich, USA) and imaged using a confocal laser scanning microscope (model TCS SP5, Leica Microsystems, Mannheim, Germany) at the University of Guelph Molecular and Cellular Imaging Facility.

### Testing anti-*Fg* bacterial strains for their ability to protect mature seed after harvest

This experiment was conducted as previously described (Mousa et al., 2016). The objective was to coat *Fg*-infected maize seeds with anti-*Fg* bacteria, dry and store the seeds at room temperature, and test for mycotoxin accumulation. For this experiment, deliberately *Fg*-infected maize seeds collected from the field with a moisture content of 17.5% were stored for 8 months, both in Falcon tubes and in envelopes to determine if the storage conditions affected the mycotoxin concentration. Ten grams of infected maize seeds were weighed per replicate per Falcon tube or sealed envelope. The anti-*Fg* bacteria were cultured in salt-reduced (5 g/L NaCl) LB broth (pH 7.2), then grown for 2 days at 30°C, with shaking at 200 rpm. The bacterial liquid cultures were then centrifuged for 10 min, followed by resuspension in LB broth to an optical density (OD_600_) of 0.4-0.6. One ml of this bacterial culture was applied to the infected maize seeds and mixed thoroughly. The negative control was seed coated with LB liquid (pH 7.2), whereas the positive control was a fungicide (PROLINE® 480 SC Foliar Fungicide, Bayer Crop Science) applied onto seeds, both at 1 mL per replicate. Proline was mixed with 0.125% v/v Agral 90 (registration #11809, Syngenta Canada, Guelph, ON).

There were 4 treatments in total: 2 anti-*Fg* bacterial treatments, a negative control (LB broth), and a positive control (Proline fungicide), with 3 replicates per treatment. After the inoculation step, the Falcon tubes and envelopes were kept in a secondary container and stored inside a cupboard in the lab at room temperature. ELISA analysis was performed to quantify DON levels in the maize seeds after 8 months of storage. For this, all seeds from each replicate were ground to a fine powder for one minute using a coffee grinder. Ten grams of these ground samples were diluted in 100 mL of deionized water followed by vigorously shaking for 3 min using a benchtop reciprocal Eberbach shaker equipped with a flask carrier (Eberbach, Corp. Ann Arbor, MI, USA). The sample extracts were allowed to sit for 3 min, allowing some particles to settle, and then 5 mL of each extract (aliquot) was filtered through a Neogen syringe filter, collecting a minimum of 3 mL into a sample collection tube. This filtrate was used for DON mycotoxin testing. Veratox for DON 5/5 (Lot #309312, NEOGEN Corporation, Lansing, USA), was used for ELISA analysis following the manufacturer’s protocol. The data were statistically modeled with a GLMM using PROC GLIMMIX and analyzed using One-Way ANOVA using Tukey’s multiple comparisons in SAS 9.4 with a significance threshold of P ≤ 0.05.

## Results

### Taxonomically diverse bacteria were cultured from unpollinated silks of diverse American maize accessions

**Figure 2:**
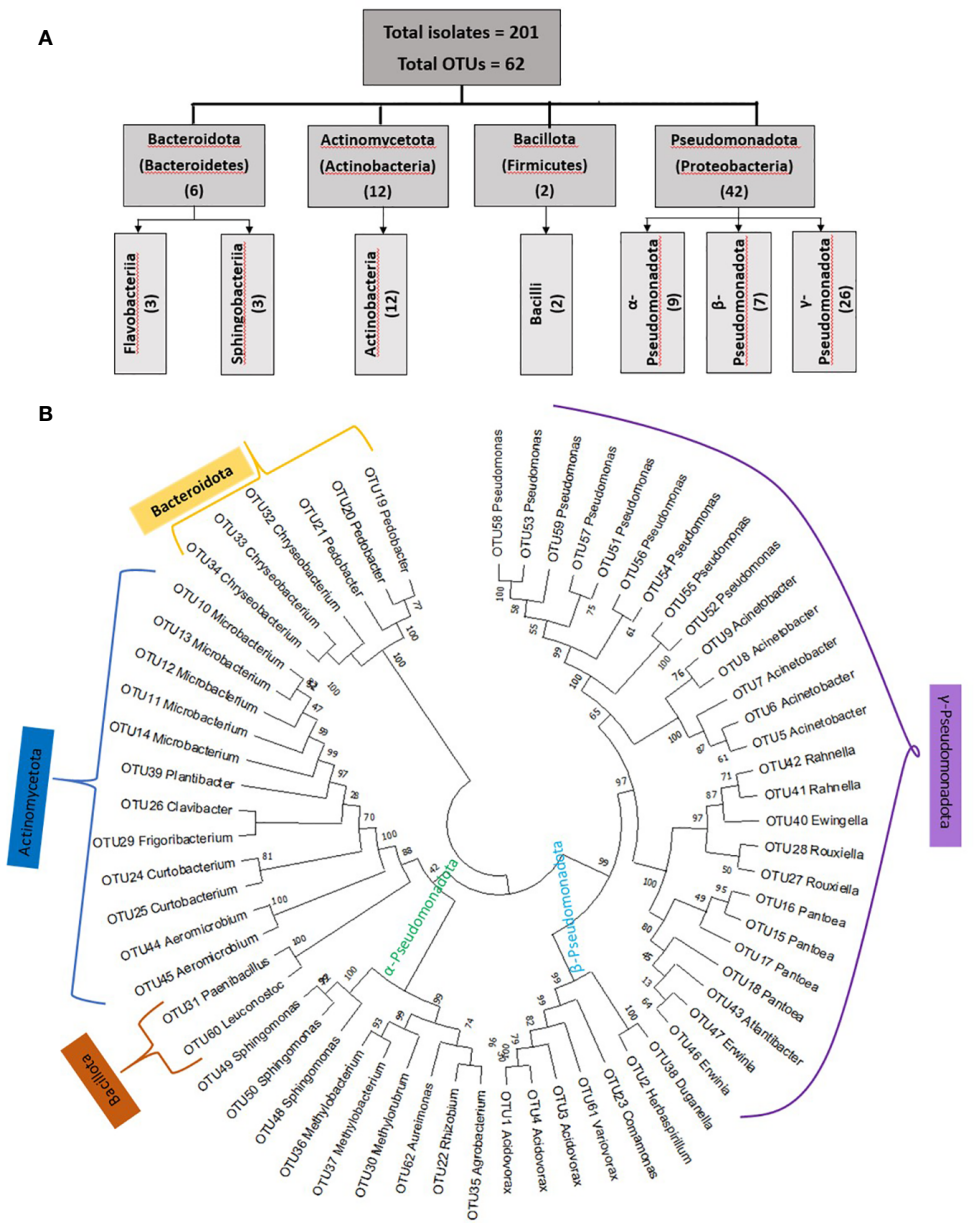
Summary of taxonomic classifications of unpollinated silk-associated bacteria cultured from diverse American maize accessions. **(A)** Diagrammatic sketch of the taxonomies at the phylum and class level based on full-length 16S RNA sequences. **(B)** Maximum likelihood (ML) phylogenetic tree of the entire bacterial population based on unique operational taxonomic units (OTUs). Bootstrap values are indicated above the branches.

**Figure 3:**
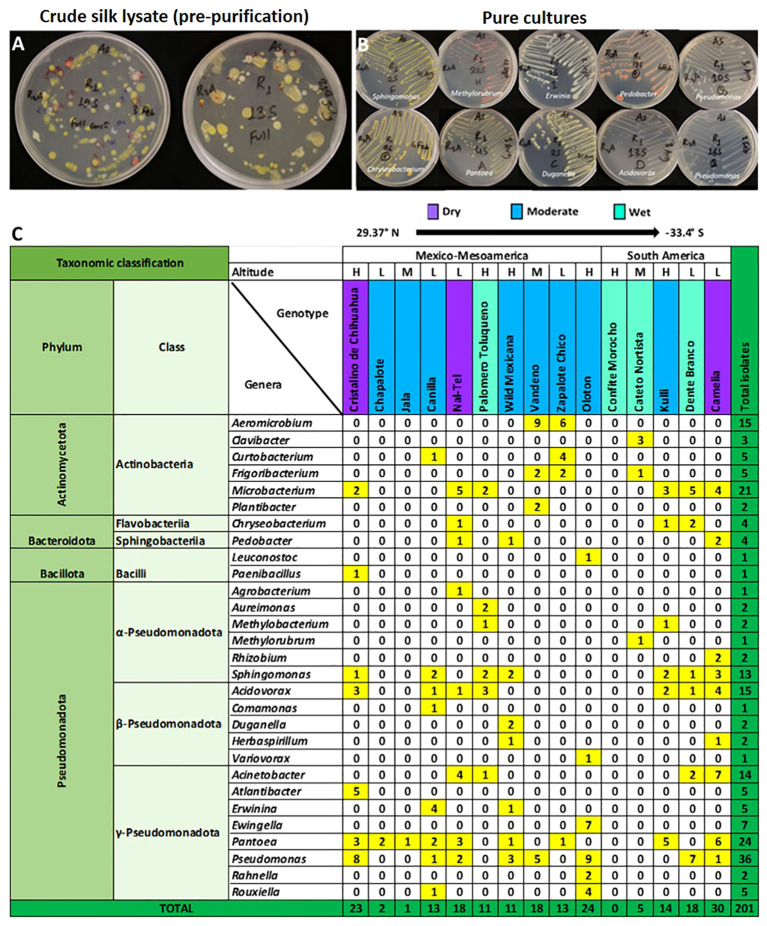
Summary of the taxonomies of unpollinated silk-associated bacteria at the phyla, class, and genus level. **(A)** Example photos of plates with crude silk lysate (pre-purification) after 5 days of incubation showing the presence of bacterial isolates with diverse colour, shape, and size. **(B)** Example photos of plates with pure cultures showing diverse bacterial genera after purification. **(C)** Phyla, class, and genera level summary of the cultured bacteria arranged by source host maize accession, arrayed by latitude from north to south. The predicted bacterial genera are based on full-length 16S Blastn searches at NCBI. The yellow and dark green colours highlight the total number of isolates (including redundant and unique sequences) belonging to the corresponding bacterial genus, arranged by host maize accession. The letter ‘H’ denotes an accession originating from a highland environment, ‘M’ denotes an accession originating from a mid-altitude environment, and ‘L’ denotes an accession originating from a lowland origin. The rainfall classification is shown in different colour codes (Dry, Moderate, and Wet). All plants were grown in a common field at Elora, Ontario, Canada.

From fifteen diverse American maize accessions including landraces and one wild teosinte (Mexicana, the minor ancestor of modern maize), all grown in a common field, a total of 201 unpollinated silk-associated bacterial isolates were cultured and taxonomically classified based on sequencing of the full-length 16S rRNA gene (**Figures 1**–**3**, **S1**; **Table 1**; **Supplementary Table 1**). The sequences were deposited in Genbank, and the accession numbers are reported **(Supplementary Table 2**). The isolates belonged to four phyla dominated by Pseudomonadota (Proteobacteria) with 140 isolates, followed by Actinomycetota (Actinobacteria) with 51 isolates, Bacteroidota (Bacteroidetes) with 8 isolates and Bacillota (Firmicutes) with 2 isolates (**Figures 1**–**3**). Within the phyla Actinobacteria and Firmicutes, all bacterial isolates belonged to only one class whereas, within Bacteroidetes and Proteobacteria, they belonged to more than one class. In total, the diverse American unpollinated silk-associated cultured library spanned 29 predicted bacterial genera, 63 predicted species, and 62 unique OTUs (**Figures 2**, **3**; **Supplementary Table 2**). Of the bacterial genera, 19 belonged to Pseudomonadota with 42 OTUs, representing more than 65% of all unpollinated silk-associated OTUs (**Figures 2A, B**, **3**). Another 6 belonged to phylum Actinomycetota with 12 unique OTUs, 2 belonged to Bacteroidota with 6 unique OTUs, and 2 belonged to Bacillota with 2 unique OTUs.

### Prevalence of culturable maize silk bacteria across maize accessions

We asked whether some unpollinated silk-associated bacteria were prevalent across maize accessions. Within phylum Pseudomonadota, the class Gammapseudomonadota was cultured from 13/15 host maize accessions studied, belonging to 8 different bacterial genera (*Atlantibacter, Rahnella, Erwinia, Pantoea, Ewingella, Rouxiella, Acinetobacter*, and *Pseudomonas*) (**Figure 3**). It is noteworthy that no bacterium was cultured from maize host Confite Morocho. Within phylum Actinomycetota, class Actinobacteria was isolated from 10/15 maize accessions, represented by six genera (*Clavibacter, Curtobacterium, Frigoribacterium, Microbacterium, Plantibacter*, and *Aeromicrobium*).

At the genus level, *Pseudomonas* was the most conserved in the unpollinated silk microbial library, accounting for 18% (36/201) of the total isolates, and was shared across 8/15 maize accessions (**Figure 3**). The second most conserved genus was *Pantoea*, accounting for 12% (24/201) of the total cultured isolates, and shared across 9/15 host accessions. *Microbacterium* was the third highest conserved genus, accounting for 10.4% (21/201) of the total cultured isolates and shared across 6/15 host accessions (**Figure 3**).

At the predicted species level, *Pantoea agglomerans* was the most prevalent, shared across 7/15 maize accessions, followed by *Microbacterium testaceum* (6/15 accessions), and *Acidovorax wautersii* (5/15 accessions) (**Supplementary Table 2**).

**Figure 4:**
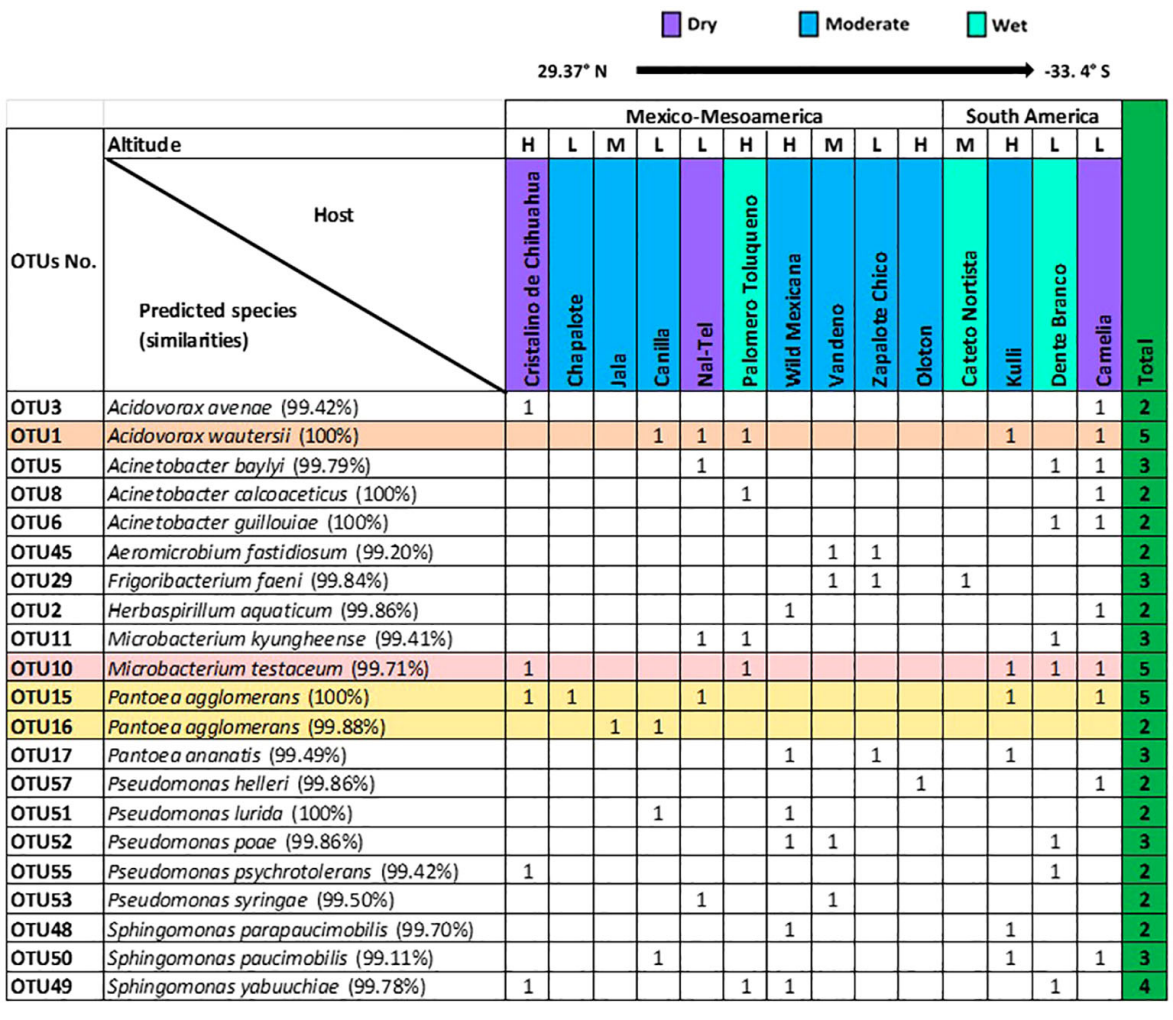
Summary of conserved OTUs of cultured unpollinated silk-associated bacteria across diverse American maize host accessions. Full-length 16S RNA sequences sharing the same genus were multi-aligned, and each unique sequence was assigned a distinct OTU number. Shown are the resulting species- and OTU-level taxonomic predictions of bacteria that were cultured from the unpollinated silks of more than one host maize accession. The most conserved OTUs are highlighted horizontally. The corresponding species prediction is noted along with the percentage identity to the top match in brackets. The data are organized by the latitude origin of source maize plants. The letter ‘H’ denotes a highland origin, ‘M’ denotes a mid-altitude origin, and ‘L’ denotes a lowland origin with respect to source maize plants. The rainfall classification is shown (Dry, Moderate, and Wet). All plants were grown in a common field in Elora, Ontario, Canada.

At the OTU level, 21 OTUs were cultured from more than one host maize accession (**Figure 4**). Of these shared OTUs, OTU1 (best match: *Acidovorax wautersii*), OTU10 (best match: *Microbacterium testaceum*), and OTU15 (best match: *Pantoea agglomerans*) were equally conserved and shared across 5/15 maize accessions (**Figure 4**).

### Unpollinated silk-associated bacteria have high intra-genus and intra-species diversity across diverse American maize accessions

Though some unpollinated silk-associated bacteria appeared to be highly conserved across diverse American maize accessions based on genus-level taxonomy (**Figure 3**), species/OTU level taxonomy revealed less conservation across host plants due to intra-genus level diversity. The 36 *Pseudomonas* isolates which appeared to be shared amongst 8 out of 15 maize accessions, actually encompassed 11 species (*P. azotoformans*, *P. cannabina, P. cerasi, P. chlororaphis, P. helleri, P. koreensis, P. lurida, P. oryzihabitans, P. poae, P. psychrotolerans*, and *P. syringae*) and 9 unique OTUs (OTU58, OTU53, OTU59, OTU57, OTU51, OTU56, OTU54, OTU55, and OTU52) (**Supplementary Table S2**; **Figures 2**, **3**, **S1**). Of these, *P. azotoformans*, *P. chlororaphis*, *P. helleri*, and *P. koreensis* were only cultured from host Oloton, *P. cannabina*, and *P. cerasi* from host Vandeno, and *P. oryzihabitans* from host Camelia (**Supplementary Table S2**). At even greater taxonomic resolution within *Pseudomonas*, at the OTU level, OTU56 (best match: *P. azotoformans*/*chlororaphis*) and OTU54 (best match: *P. koreensis*) were only cultured from host Oloton, while OTU58 (best match: *P. cannabina*) and OTU59 (best match: *P. cerasi*) were only isolated from Vandeno (**Supplementary Table S2**). Similarly, the 24 isolates of *Pantoea*, apparently conserved amongst 9/15 maize accessions, spanned four diverse species and 4 unique OTUs. Of these*, P. anthophila* was only cultured from host Camelia, and *P. dispersa* from host Kulli. At the OTU level, OTU18 (best match: *P. dispersa*) was only cultured from Kulli. Similarly, the 21 isolates of *Microbacterium*, which also appeared conserved amongst 6/15 host accessions, included 3 species and 5 unique OTUs. Within this genus, *M. neimengense* was only cultured from host Nal-Tel. At higher resolution, OTU14 (best match: *M. neimengense*) was isolated from Nal-Tel, while OTU12 (best match: *M. testaceum*) was only cultured from host Dente Branco, and OTU13 (best match: *M. testaceum*) was only isolated from Nal-Tel (**Supplementary Table S2**; **Figures 2**, **3**).

### Maize host-specific silk microbiota

Amongst the maize host accessions, some of the silk-associated bacterial genera (14 in total) were uniquely cultured from a single maize accession (**Figures 3**, **S2**; **Supplementary Table S2**), though this could have occurred by random chance. For example, the genera *Clavibacter* and *Methylorubrum* were only cultured from landrace Cateto Nortista; *Plantibacter* from Vandeno; *Paenibacillus* and *Atlantibacter* from Cristalino de Chihuahua; *Agrobacterium* from Nal-Tel; *Aureimonas* from Palomero Toluqueno; *Rhizobium* from Camelia; *Comamonas* from Canilla; and *Duganella* from Mexicana. The landrace Oloton yielded unique genera (*Leuconostoc*, *Variovorax*, *Ewingella*, *Rahnella*). Furthermore, 8/62 OTUs (OTU28, OTU40, OTU41, OTU42, OTU54, OTU56, OTU60, and OTU61) in this study were unique to this landrace (**Figures 2**, **3**, **S1**; **Supplementary Table S2**).

Descriptively, out of the total 201 silk-associated isolates cultured, 30/201 (~15%) were isolated from maize landrace Camelia, belonging to 9 bacterial genera and 14 unique OTUs (**Figure 3**; **Supplementary Table S2**), out of which 10 OTUs were shared with other host accessions (**Figures 4**, **S3**). The second highest number of isolates (24/201, ~12%) was cultured from the maize landrace Oloton, belonging to 6 different genera and 9 unique OTUs, out of which only one OTU was shared with other host accessions, suggesting these bacterial OTUs were potentially unique to this specific maize host. The next highest number of isolates (23/201, ~11.5%) was retrieved from landrace Cristalino de Chihuahua, belonging to 7 bacterial genera and 7 unique OTUs, out of which 5 OTUs were shared with other maize accessions (**Figures 3**, **4**). No bacterium was cultured from maize host Confite Morocho. Only a single bacterial isolate was captured from Jala, two from Chapalote, and 5 from Cateto Nortista (**Figures 3**, **S2**).

### Testing the ability of cultured unpollinated silk-associated bacteria to suppress *Fusarium graminearum in vitro*

**Figure 5:**
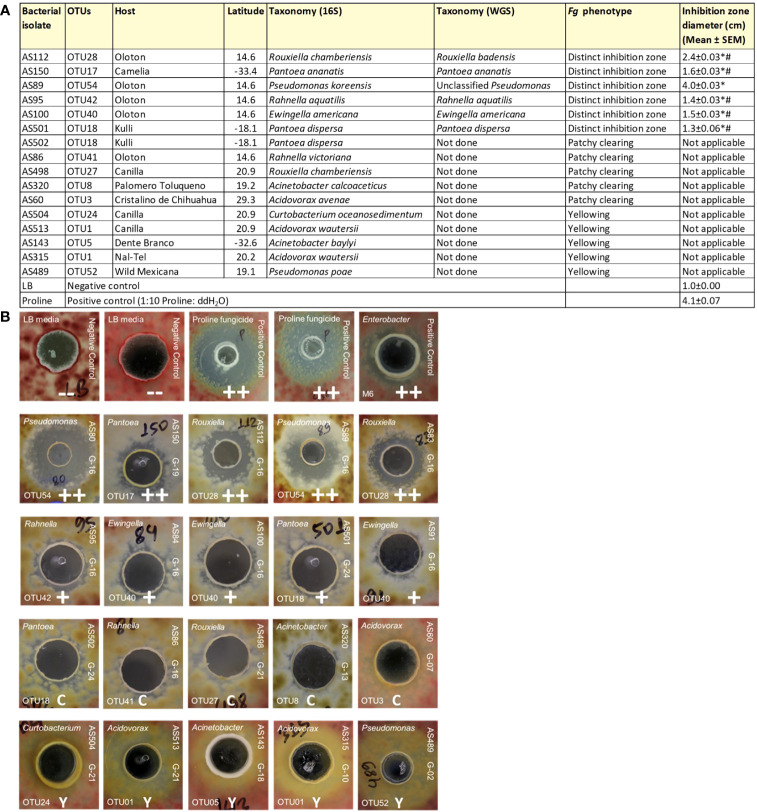
Dual culture assays to test the ability of unpollinated silk-associated bacteria to suppress *Fusarium graminearum (Fg) in vitro*. In these assays, each agar plate was embedded with *Fg* (pink background), then a well was created, into which was pipetted a single unpollinated silk-associated bacterial liquid culture or control solution. **(A)** Summary of the unpollinated silk-associated bacterial isolates that showed anti-*Fg* phenotypes along with their host source, taxonomies based on 16S and whole genome sequencing, and their zone of inhibition against *Fg* (in cm). An asterisk (*) indicates that the zone of inhibition is significantly different from the LB buffer negative control; and a number sign (^#^) indicates that the zone of inhibition is significantly different from the Proline fungicide positive control (P ≤ 0.05, see Methods). **(B)** Results of the dual culture assay with representative pictures of the effects of individual silk-associated bacteria on *Fg*. The rows on the top are the control treatments: LB buffer negative control with no zone of inhibition (–), Proline fungicide positive control, and M6 positive control which is *Enterobacter* sp. M6, previously shown to have anti-*Fg* activity (positive biological control). The isolates in the second row have a strong zone of *Fg* inhibition (++); third-row isolates cause minor inhibition (+); fourth-row isolates have a clearing (C) effect on *Fg* without any organized zone of inhibition; and fifth-row isolates have a yellowing (Y) effect on *Fg*. For each unpollinated silk isolate, the strain identifier information is noted: 16S BLAST genus prediction (top left corner), OTU number (bottom left corner), isolate sample identifier (ID) (top right side), and the source maize accession ID (bottom right side). The host maize accession IDs are as follows: G16, Oloton; G19, Camelia; G24, Kulli; G21, Canilla; G13, Palomero Toluqueno; G07, Cristalino de Chihuahua; G18, Dente Branco; G10, Nal-Tel; G02, *Zea mays* ssp. *mexicana*.

As noted earlier, the fertilization process in maize requires its style tissues (silks) to become exposed to the environment to capture and transmit pollen with its resident sperm nuclei to eggs. This makes progeny seed susceptible to fungal pathogens, which use the same style/silk route to enter maize ears and reach kernels. Therefore, we hypothesized that there may have been selection pressure on silks by indigenous farmers to recruit protective bacteria to combat such fungal pathogens. To screen bacteria for their ability to suppress the growth of *Fg in vitro* using dual culture assays, a total of 112 unpollinated silk-associated bacterial isolates were selected which maximized both bacterial species and host maize diversity (**Supplementary Table S2**). In total, 10 bacterial isolates consistently and strongly inhibited *Fg* growth (**Supplementary Table S2**; **Figure 5B**). Among these, duplicate strains were removed that shared the identical 16S rRNA sequence based on multiple sequence alignments which originated from the same maize accession; from these, a single strain was randomly selected, resulting in 6 unique anti-*Fg* strains. These results were statistically analyzed (**Figure 5A**). The inhibitory zones of all these 6 strong anti-*Fg* strains were significantly different (P ≤ 0.05) from the inhibitory zones of LB (the negative control), as well as Proline fungicide (the positive control), except for the strain AS89, which showed no significant difference with Proline (at P ≤ 0.05).

These anti-*Fg* strains belonged to diverse bacterial genera; however, most of them were cultured from the same host accession, Oloton (**Figures 5A, B**). Except for strain AS150 (OTU17), all the remaining anti-*Fg* strains were unique to a specific maize accession: AS112 (OTU28), AS89 (OTU54), AS95 (OTU42), and AS100 (OTU40) were unique to the landrace Oloton, while AS501 (OTU18) was unique to landrace Kulli based on the 16S sequences. Strain AS89, belonging to *Pseudomonas* (OTU54, predicted to be *Pseudomonas koreensis*, 99.41% identity) resulted in the largest zone of inhibition (**Figure 5A**). Of these potent anti-*Fg* strains, only one strain AS150 belonged to a maize accession that originated from lowland and/or dry environments (500-800 mm rainfall) whereas the remaining strains belonged to hosts originating from highlands with moderate rainfall (>800 to <1270 mm) (**Figures 5A**, **S2**).

In addition to the above potent anti-*Fg* bacteria, some isolates displayed patchy clearing effects with respect to *Fg* growth without distinct zones of inhibition, while others caused yellowing of *Fg* (**Figure 5B**; **Supplementary Table S2**).

### Suppression of Gibberella ear rot by silk-associated anti-*Fg* strains in greenhouse trials

To test whether the *in vitro* anti-*Fg* activities of unpollinated-silk associated bacteria were biologically relevant *in planta* (e.g. in pollinated silks through which *Fg* invades grain at the time of pollination), replicated greenhouse trials were conducted where the bacteria were sprayed onto silks before inoculation with *Fg* pathogen, followed by a repeated bacterial spray. Two unpollinated silk-associated bacterial isolates were selected for greenhouse trials due to space limitations. They were selected based on the results of the dual culture assays and human safety concerns including initial human biosafety risk group assessments based on 16S-based phylogenetic tree reconstruction (**Figure S4A**, see detailed figure legend) and clinical antibiotic disc susceptibility testing (**Figure S4B**). Only biosafety Risk Group 1 strains (safest group) with low/average antibiotic resistance were selected. The bacterial treatments were AS112 (*Rouxiella* OTU28, from landrace Oloton), and AS150 (*Pantoea*, OTU17, from landrace Camelia) (**Figure 5A**).

#### Greenhouse trial results: visual assessment of the percentage of cob length showing disease

**Figure 6:**
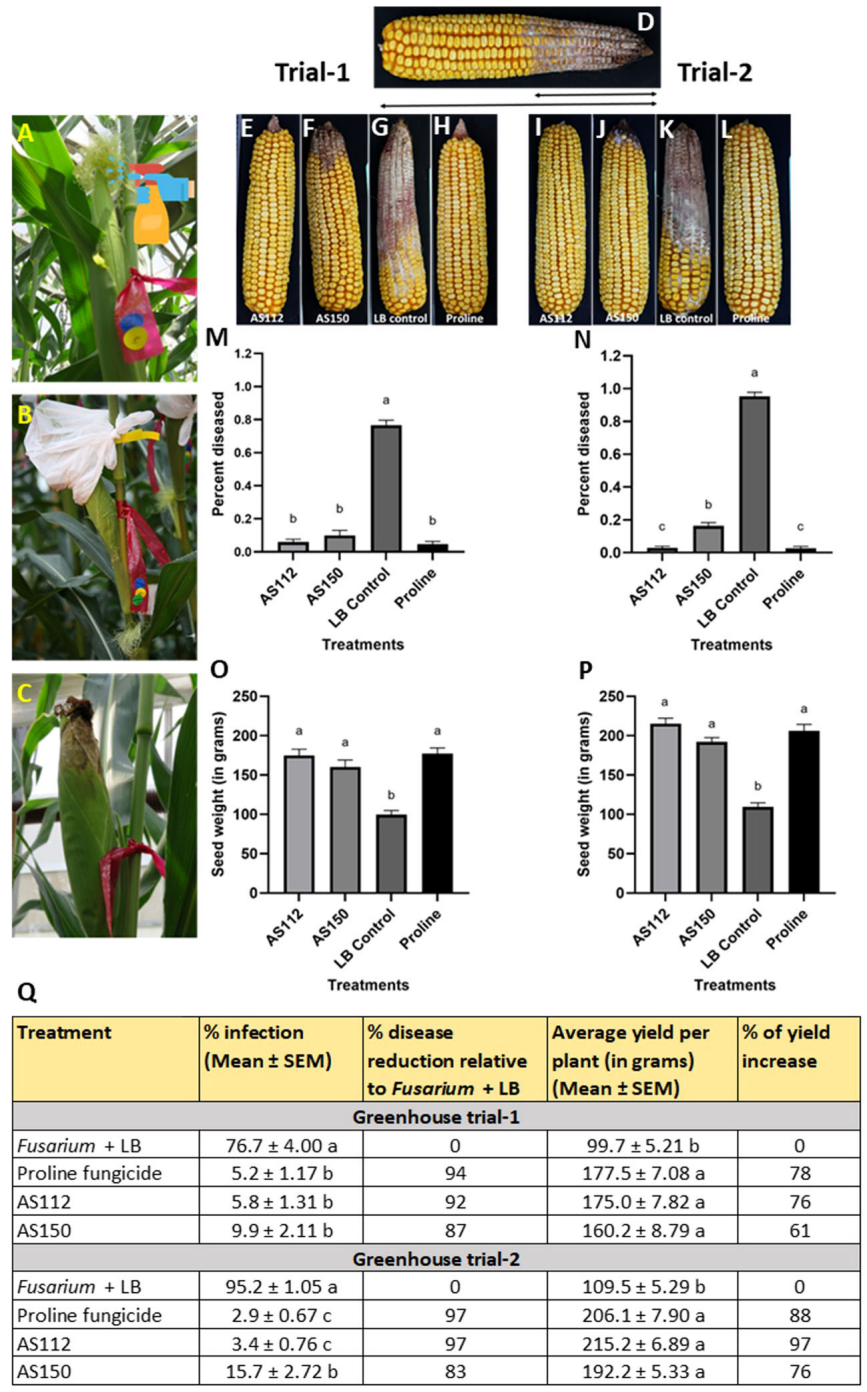
Greenhouse trials to test the ability of unpollinated silk-associated bacteria to suppress Gibberella ear rot (GER) in modern hybrid maize. **(A-C)** Application treatment procedure: all silks were sprayed with a bacterial strain or a control solution before inoculation with *Fusarium graminearum* (*Fg*), and then again after (except Proline). The cobs treated with *Fg* were covered with plastic bags which were removed 10 days after *Fg* application. **(D)** Picture of a mature cob illustrating the visual disease scoring method used which was quantified as the proportion of the diseased cob relative to the total length of the cob. The diseased portion was measured from tip to base (average of 4 measurements from 4 different sides of the cob), then multiplied by 100 to calculate the percentage of disease. **(E-H)** Representative treated cobs from each treatment in Trial 1, and **(I-L)** Trial 2. **(M-P)** Quantification of the effects of different treatments on GER suppression: **(M, N)** percentage diseased ear in **(M)** Trial 1 and **(N)** Trial 2, and **(O, P)** average seed weight (in grams) in Trial 1 **(O)** and Trial 2 **(P)**. **(Q)** The effect of the bacterial strains on the percentage disease reduction and percentage yield increase relative to the *Fusarium* + LB buffer treatment (negative control). For both measurements, n= 6 blocks per treatment, completely randomized (with 4 plants per treatment per block). Error bars indicate the standard error of the mean (SEM). The different letters on the top of the histograms **(M–P)** and to the right of infection or yield measurements in **(Q)** indicate that the mean values are significantly different from each other (P ≤ 0.05, see Methods).

Initially, GER disease was scored visually at the whole cob level by measuring the fraction of the cob length that was diseased, a measure of disease spread, since *Fg* was inoculated at the cob tip. The results showed that the silk-associated bacteria resulted in significant reductions (P ≤ 0.05) in GER disease symptoms compared to the *Fusarium* + LB buffer treatment (**Figure 6**). Disease symptom reductions ranged from 87 to 92% in Trial 1, and 83 to 97% in Trial 2. Strain AS112 showed no significant difference (P ≤ 0.05) in disease suppression as compared to the positive Proline fungicide control, in both trials; while strain AS150 showed no significant difference compared to the fungicide treatment in Trial 1. Both of these bacterial strains showed a significant increase (P ≤ 0.05) in grain yield in both trials when compared to the negative control (*Fusarium* + LB buffer treatment) but no significant difference (P ≤ 0.05) when compared to the Proline fungicide treatment (**Figure 6Q**).

#### Greenhouse trial results: visual assessment of disease severity at the individual kernel level

**Figure 7:**
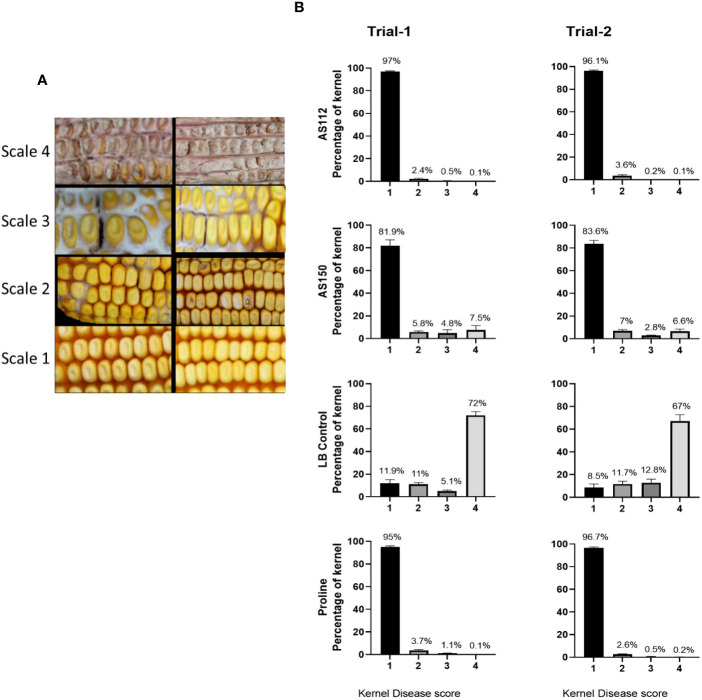
Testing the ability of unpollinated silk-associated bacteria to suppress Gibberella ear rot (GER) in modern hybrid maize based on visual disease severity assessment at the individual kernel level. All silks were sprayed with a bacterial strain or a control solution before inoculation with *Fusarium graminearum*, and then again after *Fusarium* (with the exception of Proline). **(A)** Representative photos of maize kernels showing the scale used for assessing the kernel disease severity visually. The scale ranged from 1 to 4, with a value of 1 indicating no infection; 2 indicating low infection; 3 indicating medium infection, and 4 indicating highly infected. The two images associated with each scale value indicate the range of phenotypes assigned to that disease score (see Methods). **(B)** The result of individual kernel disease severity assessment is presented in percentages, which is the fraction of the kernel population with each disease score. Each percentage is the mean of 24 cobs (plants). Proline is the positive fungicide control; LB is the negative buffer control; AS112 and AS150 are the bacterial treatments used in the trials. All error bars indicate the standard error of the mean (SEM).

Individual kernels of the treated cobs that were visually assessed at the whole cob level were again scored for ear rot symptoms. A visual rating scale from 1 to 4 was used with “4” indicating the most severely infected kernels, and “1” assigned to kernels showing no visual disease symptoms (**Figure 7A**). In both greenhouse trials, both silk-associated bacterial sprays resulted in a significant increase (P ≤ 0.05) in the percentage of seeds with no visual disease symptoms (scale of 1), compared to the *Fg*-only treated kernels (**Figure 7B**; **Supplementary Tables 4A.7, 4A.8, 4B.7, 4B.8**) and a simultaneous decrease in the percentage of seeds with severe disease symptoms (scale of 4) (**Figure 7B**; **Supplementary Tables 4A.1, 4A.2, 4B.1, 4B.2**). Among these anti-*Fg* strains, AS112 (*Rouxiella* OTU28, from Oloton) had a significantly lower percentage of diseased kernels (scale of 4) as compared to AS150 (*Pantoea* OTU17, from Camelia) (**Supplementary Tables 4A.1, 4A.2, 4B.1, 4B.2**), consistent with the whole cob level results.

#### Greenhouse trial results: grain mycotoxin accumulation

We hypothesized that indigenous farmers in the Americas may have inadvertently selected unpollinated silk-associated bacteria that could suppress *Fg*-associated mycotoxins in the grain, by associating diseased seed with human and/or livestock illness following their consumption. To test this hypothesis, seeds of the same treated cobs reported earlier for disease symptoms were pooled from each plant per treatment per block, resulting in a total of 6 replicates per treatment. Liquid chromatography-tandem mass spectrometry (LC-MS/MS) was used to quantify concentrations of the following *Fg*-producing mycotoxins in grain immediately after harvest: deoxynivalenol (DON), 3-acetyl-deoxynivalenol (3ADON), 15-acetyl-deoxynivalenol (15ADON), DON-3-glucoside, and zearalenone.

Overall, both silk-associated bacterial strains resulted in significant reductions (P ≤ 0.05) of mycotoxins ranging from 70-100% (except DON-3-glucoside in Trial 2) compared to the *Fg* + LB buffer-treated plants (**Table 2**). Furthermore, treatment with strain AS112 showed higher reductions in mycotoxin concentrations in Trial 2, while strain AS150 was better in Trial 1 (**Table 2**). This result also matched the earlier cob-level visual disease scoring.

**Table 2:** Results of greenhouse Trials 1 and 2, showing the reduction in mycotoxin accumulation in *Fg*-infected grains following treatment with the unpollinated silk-associated anti-*Fg* bacterial strains.

Treatment	Trial 1		Trial 2	
	DON content (mean ± SEM)*	% DON reduction relative to *Fusarium* + LB	DON content (mean ± SEM)*	% DON reduction relative to *Fusarium* + LB
*Fusarium* + LB	222332.7 ± 26043.31	0	228890.0 ± 16449.41	0
Proline fungicide	2036.3 ± 246.98*	99	1084.3 ± 325.19*	100
AS112	25360.9 ± 2252.26*	89	4894.8 ± 1987.70*	98
AS150	20890.9 ± 9709.10*	91	47186.3 ± 15116.59*	79
	3ADON content (mean ± SEM)*	% 3ADON reduction relative to *Fusarium* + LB	3ADON content (mean ± SEM)*	% 3ADON reduction relative to *Fusarium* + LB
*Fusarium* + LB	2845.6 ± 387.93	0	3015.1 ± 386.27	0
Proline fungicide	15.0 ± 2.58*	99	12.6 ± 3.71*	100
AS112	266.9 ± 242.82*	91	39.3 ± 11.14*	99
AS150	195.9 ± 107.27*	93	368.9 ± 95.75*	88
	15ADON content (mean ± SEM)*	% 15ADON reduction relative to *Fusarium* + LB	15ADON content (mean ± SEM)*	% 15ADON reduction relative to *Fusarium* + LB
*Fusarium* + LB	1683.5 ± 392.82	0	1948.0 ± 105.21	0
Proline fungicide	5.5 ± 2.09*	100	4.5 ± 1.66*	100
AS112	251.1 ± 187.12*	85	138.7 ± 41.87*	93
AS150	127.8 ± 53.31*	92	503.8 ± 189.93*	74
	DON3-glucoside content (mean ± SEM)*	% D3-glucoside reduction relative to *Fusarium* + LB	DON3-glucoside content (mean ± SEM)*	% D3-glucoside reduction relative to *Fusarium* + LB
*Fusarium* + LB	29028.0 ± 6428.94	0	22650.0 ± 5070.14	0
Proline fungicide	28.8 ± 28.46*	100	35.0 ± 34.98*	100
AS112	6714.4 ± 682.68*	77	900.5 ± 305.70*	96
AS150	3666.7 ± 1764.78*	87	13741.3 ± 5658.31	39
	Zearalenone content (mean ± SEM)*	% Zearalenone reduction relative to *Fusarium* + LB	Zearalenone content (mean ± SEM)*	% Zearalenone reduction relative to *Fusarium* + LB
*Fusarium* + LB	47156.8 ± 11133.15	0	54430.4 ± 7406.29	0
Proline fungicide	211.9 ± 194.27*	100	14.8 ± 7.28*	100
AS112	8376.9 ± 885.05*	82	180.0 ± 79.70*	100
AS150	347.9 ± 184.11*	99	10576.8 ± 4921.02*	81

All concentrations are noted in parts per billion (ppb). Asterisks indicate that the value is significantly different from the respective negative control treatment (*Fusarium* + LB buffer) (*=P ≤ 0.05, see Methods).

### *In vitro* interactions between silk-associated anti-*Fg* bacterial strains and *Fg* using light microscopy

**Figure 8:**
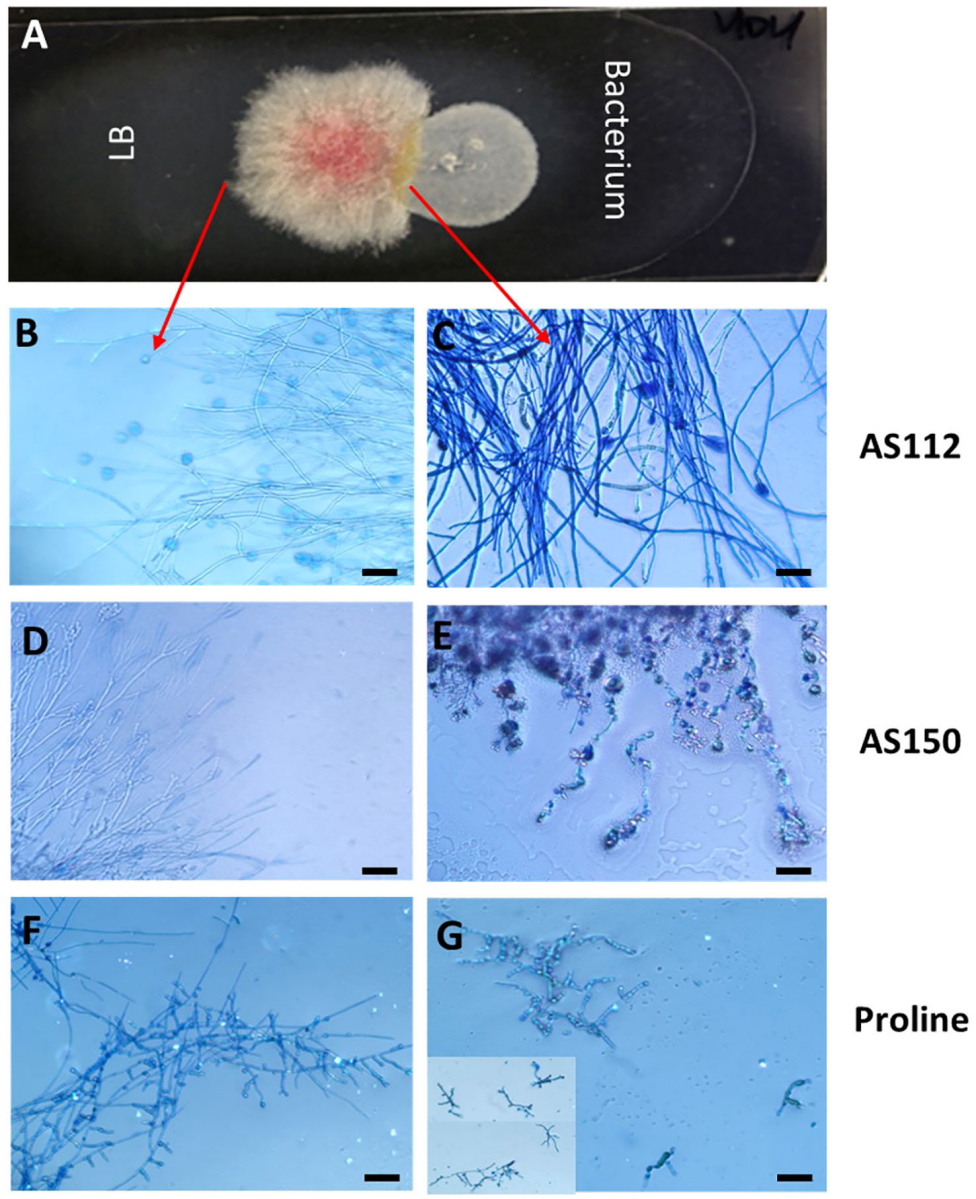
The interactions of unpollinated silk-associated anti-*Fg* bacterial strains with *Fusarium graminearum* (*Fg*) *in vitro* after staining with the vitality stain, Evans blue. **(A)** The methodology used: Potato dextrose agar-coated microscope slide with *Fg* in the center, flanked by an anti-*Fg* bacterial strain (right) or LB buffer control (left). After 24 h of co-incubation, Evan’s blue was added and then visualized using a light microscope: only dead *Fg* hyphae take up the stain. **(B, D, F)** *Fg* hyphae on the control side (no bacteria) and **(C, E, G)** the corresponding *Fg* hyphae on the side exposed to the different treatments as follows: **(C)** strain AS112, **(E)** strain AS150, and **(G)** Proline fungicide. The inset in panel **(G)** is to show multiple *Fg* hyphae. The scale bar in all images is 5 µM.

The two silk-associated bacteria (AS112 and AS150) that inhibited *Fg* growth *in vitro* and *in planta* were visualized on microscopic slides to better understand their antifungal mode(s) of action (**Figure 8**). Each bacterium was grown alongside *Fg*, or a buffer control (LB) (**Figure 8A**), then stained with Evans blue, a vitality stain that stains fungal mycelia blue when dead (Gaff and Okong’O-Ogola, 1971). Microscopic analysis showed that *Fg* hyphae in contact with strain AS112 remained intact and appeared normal but stained darker blue compared to LB buffer exposure (**Figures 8B, C**). In the case of strain AS150, however, the *Fg* hyphae near the bacterial strain not only stained dark blue but showed morphological deformities (**Figures 8D, E**). Since AS112/AS150 exposed *Fg* hyphae took up more Evan’s blue, suggestive of death, we conclude that these strains have fungicidal activity against *Fg.*

### Confocal fluorescence imaging of interactions between AS112 and *Fusarium graminearum* on living silks

**Figure 9:**
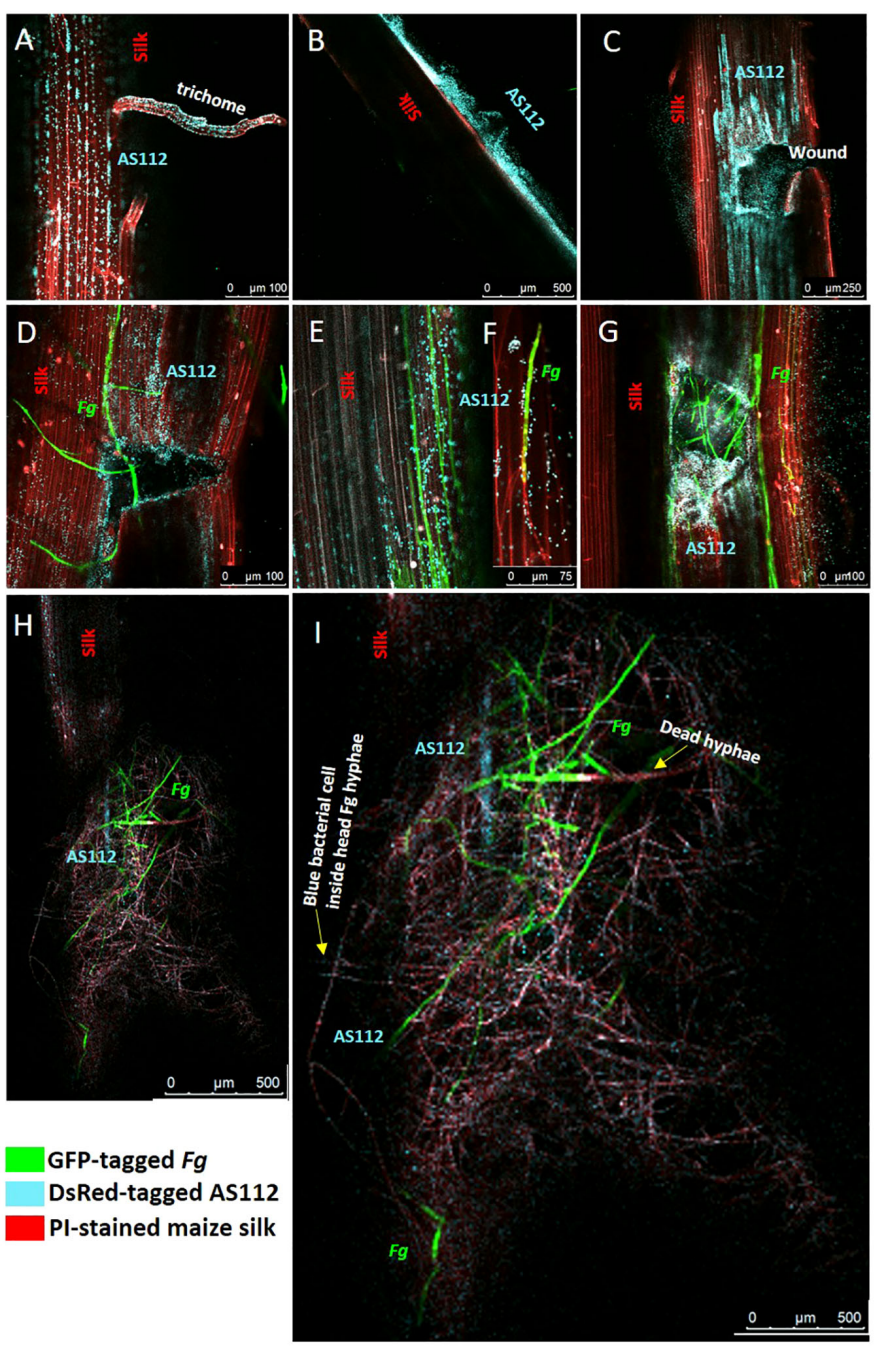
Confocal fluorescence microscopy imaging of maize silks showing interactions between AS112 and *Fusarium graminearum* (*Fg*). AS112 was tagged with DsRed, but false coloured blue, while *Fg* was tagged with GFP (green), and silks were stained with propidium iodide (red). Some silks were mechanically wounded to mimic insect damage, which promotes *Fg* infection. **(A)** AS112 colonizing a stigmatic trichome in the absence of *Fg*. **(B)** AS112 forming a biofilm on the silk epidermis in the absence of *Fg*. **(C)** AS112 heavily colonizing at and near a wound site in the absence of *Fg.* **(D)** AS112 colonizing a wound site in the presence of *Fg*. **(E, F)** AS112 colonizing *Fg* hyphae on the silk epidermis, leading to apparent *Fg* hyphal death (hyphae turning red and no longer expressing GFP). **(G)** AS112 heavily colonizing a wound site in the presence of *Fg*. **(H, I)** Residual AS112 colonization associated with a large mass of apparently dead *Fg* hyphae (red without GFP expression). Once *Fg* hyphae appear to die, the bacterial cells fluoresce less blue (this image was brightened to make bacterial cells more visible).

The previous greenhouse trials indicated AS112 could inhibit *Fg* when sprayed onto silks, which was then visualized on a light microscope, suggesting it has fungicidal activity *in vitro*. To further understand the mode of action of the anti-*Fg* bacterial strain AS112 on silk channels, intact silks from detached cobs were used, co-applied with fluorescently tagged AS112 and *Fg*, and visualized using confocal scanning fluorescence microscopy. AS112 was tagged with fluorescent protein DsRed (digitally transformed to a blue colour), *Fg* using GFP (green colour), and the silk cells were stained with propidium iodide (red colour) (**Figure 9**).

The confocal results showed that before *Fg* infection, the anti-*Fg* bacterial strain AS112 could: colonize trichomes (stigmatic hair), which are also known to be the susceptible entry points of *Fg* (**Figure 9A**); form biofilm structures on the silk epidermal surface (**Figure 9B**); and heavily colonize silk wound sites (**Figure 9C**). After *Fg* infection on silks, AS112 could colonize wound sites infected with *Fg* but seemed to be more attracted to the wounds rather than to *Fg* (**Figures 9D, G**); in other images, AS112 could colonize the *Fg* hyphae directly (**Figures 9E, F**), and was associated with large masses of dead *Fg* hyphae (**Figures 9H, I**). Overall, combined with the ability to suppress *Fg* disease in the greenhouse trials, these results suggested that AS112 has both preventative and protective activities against *Fg* on living silks.

### Effect of the anti-*Fg* bacterial strains on protecting seed progeny after harvest

To further protect the genetic contribution of the female gametes, we hypothesized that the maternally derived silk-associated bacteria may protect progeny seed from the further progression of *Fg* after the seed had dispersed from the mother plant. To test this hypothesis, *Fg* field-infected grain was treated with the silk-associated anti-*Fg* bacterial strains or controls (LB buffer as the negative control; and Proline fungicide as the positive control), then stored for 8 months under low humidity conditions (sealed envelopes) or high humidity (sealed Falcon tubes), the latter known to promote *Fg* growth (Munkvold, 2003; Thapa et al., 2021), and then finally quantified for DON mycotoxin concentrations using ELISA. The low humidity condition resulted in a non-significant difference (P ≤ 0.05) in DON mycotoxin between the negative and positive controls. However, under high humidity conditions, maize seeds treated with strain AS150 showed a significant reduction (P ≤ 0.05, equaling 72%) in DON concentration, compared to the LB buffer negative control (**Table 3**). The impact of strain AS112 was only significant at P ≤ 0.10 (mean reduction of 53%) with respect to its efficacy compared to the buffer control (**Table 3**).

**Table 3:** Testing the ability of silk-associated anti-*Fg* bacterial strains to protect seed after harvest.

Treatment	Host	DON content (in ppm) (Mean ± SEM)*	% of DON reduction
Grains stored in Falcon tubes (High humidity)			
LB Buffer control		13.0 ± 1.57	0
Proline fungicide		3.5 ± 1.28 *	73
AS112	Oloton	6.1 ± 2.69 **	53
AS150	Camelia	3.6 ± 1.19 *	72
Grains stored in sealed envelopes (Low humidity)			
LB Buffer control		7.9 ± 3.50	0
Proline fungicide		3.5 ± 0.52	56
AS112	Oloton	5.5 ± 0.90	30
AS150	Camelia	3.8 ± 0.61	51

Asterisks indicate that the value is significantly different from the respective negative control treatment (LB buffer control) (*=P ≤ 0.05; **=P ≤ 0.10, see Methods).

## Discussion

During the fertilization process in flowering plants, there would be selection on the maternal parent to promote progeny fitness by protecting the exposed style/silk (male gamete fertilization channel) and their connected ovules and future seeds from environmental pathogens such as *Fg*, which enter from the air and also hitchhike on the pollen (Card et al., 2007; Thompson and Raizada, 2018; Khalaf et al., 2021). Here, anti-fungal activity was discovered in bacteria cultured from unpollinated maize silks of ancient maize accessions, and their beneficial roles were validated in living silks of a modern maize hybrid. These bacteria were sprayed onto silks of modern maize to mimic the route via which *Fg* enters developing seeds. The assays suggested that the silk-associated bacteria could reduce *Fusarium* infection along the male gamete fertilization channel by forming what appeared to be protective barriers on known *Fg* entry points on silks (trichomes, wound sites) including forming dense biofilms on the silk epidermis, but also subsequently having the ability to colonize *Fg* hyphae directly after infection. Additionally, these maternally derived bacteria could protect the seed after harvest, when no longer attached to the mother.

### Comparison to prior maize silk microbiome studies

Here, the unpollinated maize silk library was found to be dominated by the bacterial genera *Pseudomonas* and *Pantoea;* furthermore, we showed that the most potent anti-*Fg* bacterial strains from the *in vitro* dual culture assays belonged to these two genera (**Figures 3**, **5**). This is only moderately consistent with the prediction from a previous study by Khalaf et al. (2021) which reported that *Pantoea* but not *Pseudomonas* increased in abundance in pollinated silks of North American maize in response to *Fg* infection. Nevertheless, in that study, *Pseudomonas* constituted 9-13% of all read counts, the third most abundant genus, but it was abundant in both *Fg*-infected and healthy silks. It might be, similar to our findings here with *Rouxiella* strain AS112 (**Figure 9**), that some silk-associated bacteria can defend against *Fg* before and after infection and hence are always present. A recent study by Diniz et al. (2022a) similarly reported that silks exposed to pollen contained *Pseudomonas*.

Khalaf et al. (2021) further reported that the genera *Herbaspirillium, Delftia, Stenotrophomonas*, and *Sphingomonas* increased in abundance in silks after *Fg* infection; though *Herbaspirillium* and *Sphingomonas* were cultured here, they were not identified as anti-*Fg* strains. Furthermore, other silk-associated bacterial genera reported by Diniz et al. (2022a) (such as *Burkholderia*, *Achromobacter*, *Serratia* and *Bacillus*) were not cultured in this study. Various studies have shown that diverse bacteria can have anti-*Fg* activity including *Bacillus* strains (Zhao et al., 2014; Mahmoud, 2016; Dimkić et al., 2022; Jimenez-Quiros et al., 2022).

There were some additional differences between this study and the prior studies. First, in both prior studies, unlike this study, the silks may have contained pollen-transmitted bacteria. Second, Khalaf et al. (2021) used North American rather than Latin American maize accessions and relied on short-read 16S-V4 region sequencing, which has limited taxonomic resolution. Finally, this study differed from Diniz et al. (2022a) in terms of the culture media and/or did not rely on culturing in the case of Khalaf et al. (2021).

### Preliminary evidence for indigenous farmer selection on silk microbiomes in the Americas

Indigenous peoples may have unknowingly selected the style microbiome to defend against an environmental pathogen that enters during pollination. Here, the *in vitro* dual culture assay identified diverse silk-associated bacterial genera from diverse maize accessions in the Americas as having anti-*Fusarium* activities (**Figure 5**). Since the anti-*Fg* bacteria belonged to only a subset of maize accessions (<50%), one possibility is that only specific indigenous peoples may have successfully selected for these traits (i.e. those that faced *Fusarium* grain disease). This interpretation requires further validation, as the plants in this study were grown in a common field for a single season; hence it is entirely possible that some of the silk-associated bacteria were derived from the Canadian field soil or soil associated with the seed banks that collected the accessions (e.g. CIMMYT in Mexico), rather than being vertically transmitted.

Interestingly, most accessions in this study that originated from the center of maize diversification (Oloton, Palomero Toluqueno, Cristalino de Chihuahua) (Matsuoka et al., 2002) were sources of anti-*Fg* bacteria, of which Oloton from Guatemala was the source of multiple potent anti-*Fg* strains (**Figure 5A**). Fungal pathogens associated with maize including *Fusarium* are prevalent in Guatemala (Rodrigo Mendoza et al., 2017). Oloton is a highland maize that was later introduced to Mexico, and is a staple food for the Mixe indigenous people in the Sierra Mixe region in southern Mexico (Pskowski, 2019); it is known to be highly resistant to pests and pathogens (CONABIO, 2017). It is noteworthy that this maize landrace predominates in a highly humid climate, which is known to favor fungal pathogens. Additionally, DON mycotoxin promotes *Fg* infection and suppresses pollen tube growth (Tejaswini, 2002; Mudge et al., 2006; Kačániová et al., 2011; Kostić et al., 2019), and also decreases host immunity (McCormick et al., 2011). These combined forces may have resulted in strong selection pressure for the Mixe people to have selected for silks to carry bacteria that could suppress *Fusarium* and DON directly (by degrading DON) or indirectly (by suppressing *Fg*) in order to protect pollen tube growth and preserve successful fertilization and hence seed yield. More evidence is needed to support this hypothesis, including whether silks directly collected from the Mixe region possess these anti-*Fg* bacteria. However, bacteria with anti-*Fg* traits were also cultured from the maize landrace Kulli which originated in Bolivia, and Camelia which originated in Chile, far away from the center of maize diversification.

It is compelling that all the OTUs shown to have anti-*Fg* activity *in vitro* were apparently host-specific (**Figure 5**). Also compelling is that anti-*Fg* bacterial strains belonged to diverse genera. Together, these results lead to the hypothesis that indigenous farmers selected for anti-*Fusarium* bacteria independently in the Americas. This selection would have occurred unknowingly by farmers, perhaps by selecting only visually healthy seed for replanting, or associating these disease symptoms with sick livestock or family members after ingestion of grain containing associated mycotoxins.

Another possibility is that indigenous farmers across the Americas were selecting silk-associated bacteria not only to combat *F. graminearum* but also other *Fusarium* species. Different *Fusarium* species and their associated mycotoxins have been reported to be prevalent in Mexico and Central America (Odjo et al., 2022). GER caused by *Fg* pathogen is favored by high levels of moisture during silking, followed by moderate temperatures and high precipitation (Sutton, 1982). However, in this study, the maize silk-associated anti-*Fusarium* bacterial isolates were cultured from maize hosts inhabiting environments ranging from wet to dry. *F. verticilloides*, which is prevalent across the Americas, favors dry environments (Miller, 2001; Sancho et al., 2017; Pfordt et al., 2020). Furthermore, a recent study (Diniz et al., 2022b) reported that some silk-associated bacterial isolates suppressed maize stalk rot severity caused by *F. verticillioides* in greenhouse trials, though they did not test *F. graminearum*. Intriguingly, the maize landrace Camelia from Chile, which possessed anti-*Fg* bacteria, is known to have resistance against ear rots caused by *F. verticillioides* (USDA-ARS, 2018). Chilean maize does suffer from mycotoxins associated with *Fg* including DON (Raj et al., 2021). Future studies are needed to test whether some of the anti-*Fg* isolates described in this study have broad-spectrum anti-*Fusarium* activity.

### Silk-associated bacteria have the potential to defend the male gamete fertilization channel against a fungal pathogen

Here, the dual culture anti-*Fg* assays identified six silk-associated bacterial isolates that strongly suppressed *Fg in vitro* as follows:

#### *Rouxiella badensis* (AS112)

This strain was derived from maize accession Oloton which was sprayed onto silks, in replicated greenhouse trials, resulting in almost complete suppression of GER disease (up to 97%) and related mycotoxin accumulation (in general, 77-91% reductions in DON, 3ADON, 15ADON, DON3-glucoside, ZEA) and significantly increased grain yield (76-97%). Additionally, AS112 demonstrated fungicidal activity against *Fg in vitro*, while confocal imaging on living silks showed that AS112 could protect silks before infection by forming what appeared to be protective barriers on silks, and after infection by colonizing and apparently killing *Fg*. The former mechanism is consistent with a study from pear which showed that epiphytic style-associated bacteria could prevent the colonization of the bacterial pathogen *Erwinia amylovora* through pre-emptive exclusion (Stockwell et al., 1999). It is noteworthy that AS112, a highly effective silk colonizer, encodes two Type III secretion system operons (**Supplementary Table 5**). It has been reported that the Type III secretion systems facilitate the secretion of effectors that suppress host defense, to promote stable colonization (Zboralski et al., 2022). In addition, AS112 could also protect progeny seeds from further *Fg* progression after being harvested from the mother plant, although high concentrations of bacteria were used. The preliminary AS112 genome mining results showed the presence of chitinase; a biosynthesis gene encoding an anti-fungal metabolite (*phzF* for phenazine); and a biosynthetic gene encoding a signaling compound known to facilitate host resistance (acetoin) (**Supplementary Table 3**). However, these observations must be further validated experimentally.

*R. badensis* has not been previously reported to suppress GER disease in maize to the best of our knowledge. It has been reported as a novel biocontrol agent against the postharvest fungal pathogens *Botrytis cinerea* and *Fusarium brachygibbosum* in strawberry fruit, affecting mycelial development (Morales-Cedeño et al., 2021). It also showed different levels of *in vitro* antagonism against 20 fungal pathogens in berries (Morales-Cedeño et al., 2021). Here, we showed that *R. badensis* (AS112) seemed to colonize wound sites on silks more than *Fg* hyphae. However, AS112 could apparently kill *Fg* by colonizing the hyphae. Combined, one interpretation of these results is that AS112 has a saprophytic mode of action, prioritizing nutrient-rich host wound sites, followed by killing and feeding on dead *Fg* hyphae. *R. badensis* colonizes decaying strawberry fruits and could suppress *Fusarium brachygibbosum* and *Botrytis cinerea* in fungal growth inhibition bioassay on strawberry fruit (Morales-Cedeño et al., 2021), consistent with it being a saprophyte in plants that perhaps protects its territory from competitors. Furthermore, despite being one of the best anti-*Fg* strains *in vitro* and confirmed by greenhouse trials, AS112 did not perform well as strain AS150 in protecting mature seed progeny after harvest. This could be because AS112 might be adapted to moist and nutrient-rich environments such as silk tissues but not dry seeds. Indeed, in addition to inhabiting decaying strawberry fruits, previous studies have shown that *R. badensis* prefers peat bog soil (Fléche-Matéos et al., 2017) and the human intestine (Yahfoufi et al., 2021) – both moist environments. Additionally, strain AS112, when cultured on media plates in the lab, could not survive for more than 4-5 days, consistent with it favoring nutrient-rich conditions.

#### *Pantoea ananatis* (AS150)

This strain was derived from maize accession, Camelia. Similar to AS112, it was sprayed onto silks, in replicated greenhouse trials, resulting in suppression of GER disease (up to 87%) and related mycotoxins accumulation (in general, 87-99% reductions in DON, 3ADON, 15ADON, DON3-glucoside, ZEA) and significantly increased grain yield (61-76%). AS150 also demonstrated fungicidal activity against *Fg in vitro* similar like AS112. Additionally, AS150 could also protect progeny seeds from further *Fg* progression after being harvested from the mother plant. *P. ananatis* has not been previously reported to suppress GER disease in maize though it has been shown to reduce DON mycotoxin by 50% in wheat infected with *Fg* (Deroo et al., 2022). Preliminary genome mining results (**Supplementary Table 3**) show that the genome of AS150 encodes *phzF*, chitinase and a gene required for acetoin biosynthesis.

*P. ananatis* has been reported as a maize seed endophyte (Rijavec et al., 2007; Johnston-Monje and Raizada, 2011). In addition to suppressing *Fg* in wheat, *P. ananatis* has been shown to inhibit the growth of the maize-associated fungus *Lecanicillium aphanocladii in vitro* (Rijavec et al., 2007). In rice, it suppressed the causative agent of rice blast disease, *Pyricularia oryzae* (Someya et al., 2003), as well as enhanced crop growth and increased yield (Megías et al., 2016; Megías et al., 2017). Interestingly, *P. ananatis* was reported to rapidly colonize plant wounds before the establishment of the pathogen (*Botrytis cinerea*) thereby suppressing mycelial growth and disease symptoms (Roca-Couso et al., 2021).

The other four silk-derived bacteria with strong anti-*Fg* activity *in vitro* and anti-fungal genes (**Supplementary Table 3**), were also not reported to control GER disease in maize but were shown to suppress *Fg*, other *Fusarium* species, or other pathogens in other crops. Specifically:

#### *Pseudomonas koreensis* (AS89)

*Pseudomonas koreensis* (AS89) was derived from maize accession Oloton, similar to AS112. This strain showed the largest ring of *Fg*-inhibition *in vitro*, even better than Proline fungicide (the positive control). However, it was not chosen for greenhouse trials because of human biosafety concerns (tolerance to specific clinical antibiotics) (**Figure S4B**). *P. koreensis* was also previously not reported to control GER in maize. It was reported to suppress *Cephalosporium maydis*, the causative agent of late wilt disease in maize, in greenhouse trials and field experiments (Ghazy and El-Nahrawy, 2021). Additionally, *P. koreensis* significantly reduced disease caused by *Pythium ultimum* in hydroponic tomato cultivation (Hultberg et al., 2010a) and *Phytophthora infestans* which causes potato late blight (Hultberg et al., 2010b).

#### *Rahnella aquatilis* (AS95)

*Rahnella aquatilis* (AS95) was also derived from Oloton which inhibited *Fg* in this study. Previous studies have reported that *R. aquatilis* as biocontrol agents for different diseases but not for GER in maize. For example, *R. aquatilis*, a rice isolate showed antagonism against *F. graminearum* and *Magnaporthe oryzae in vitro* (Sun et al., 2020). It is also reported as a biocontrol agent against grapevine crown gall (Chen et al., 2007), apple fire blight (Abo-Elyousr et al., 2011), and fruit storage rots (Navarta et al., 2011).

#### *Ewingella americana* (AS100)

AS100 was again derived from Oloton, and was one of the anti-*Fg* strains in this study. *E. americana* has not been previously shown to suppress GER disease in maize. It has been reported as a maize seed endophyte (Shiomi et al., 2008), able to enhance maize growth (Rana et al., 2021) and suppress northern corn leaf blight disease in maize (Shiomi et al., 2015).

#### *Pantoea dispersa* (AS501)

It was cultured from maize accession, Kulli, and showed *Fg* inhibition *in vitro*. *P. dispersa* is known to be an endophyte of maize and wheat, reported to promote growth in maize, wheat, and rice (Suman et al., 2020; Fernando Amezquita-Aviles et al., 2022; Shovitri et al., 2022). Most relevant to this study, it has been shown to be antagonistic to *Fusarium moniliforme* as well as *Rhizoctonia solani* pathogens of maize (Swamy et al., 2022). It strongly inhibited mycelium growth and spore germination *in vitro* of the pathogenic fungus *Ceratocytis fimbriata*, the causative agent of black rot in sweet potatoes, and inhibited its growth *in planta*, suggesting *P. dispersa* has fungicidal rather than fungistatic activity (Jiang et al., 2019).

### Exploiting plant maternal reproductive tissue as a source of disease-suppressive bacteria

In humans, the resident microbiota of the female reproductive tract plays a role in the maintenance of the mother’s health as well as in protecting babies from environmental infections before and after birth (Feng and Liu, 2022). In flowering plants such as maize, the transmission of different fungal pathogens occurs during the flowering phase when the male parent (pollen grains) and the maternal parent contact each other indirectly during pollination (Antonovics, 2005). Such fungal pathogens including *Fg* affect the health of future progeny seed in part by depositing hazardous mycotoxins, ultimately jeopardizing the contribution of the male gamete. DON mycotoxin also reduces the health of humans, cattle, poultry, and swine, especially lactating animals, with strict limits placed by different jurisdictions (Canadian Food Inspection Agency, 2015) which ultimately limit the profits of farmers globally (Konkin et al., 2022). In maize, pollination is correlated with changes to the innate plant defense system; the flavonoid antioxidant capacity decreases as maize silks mature, which might contribute to increased ear rot susceptibility after pollination (Rahman and Wan Rosli, 2014). To control such fungal pathogens, this study adds to emerging studies that the natural microbiome of disease-target tissue may have evolved or been selected to suppress disease (Stockwell et al., 1999; Cui et al., 2021; Khalaf et al., 2021). In particular, the style/silk provides an excellent niche for microbial colonization since it is rich in lipids, proteins, carbohydrates, minerals, and vitamins, and also has a high moisture content (Guo et al., 2009). Moving forward, our prior study reported seasonal variation of the pollinated silk-associated microbiome in response to *Fg* (Khalaf et al., 2021); therefore, the next focus should be on metagenome profiling and breeding for host genes that stabilize the defensive silk microbiome across seasons, environments and varieties. Since several studies, including now this one, have reported that wild and ancient relatives of crops possess an untapped reservoir of beneficial microbes (Johnston-Monje and Raizada, 2011; Yokoya et al., 2017; Preece and Peñuelas, 2020), the style tissues of diverse modern crops and ancient landraces selected by indigenous farmers, should be explored for their microbiome.

## Conclusion

In plants and animals, the mother has a major role in assuring the fitness of her future progeny (Galloway, 2005; Chettoor et al., 2016; Nguyen et al., 2021). In plants, the maternal-style tissue becomes susceptible to environmental pathogens due to its exposure to the ambient environment during the fertilization process. Here, we presented evidence from maize to suggest that in plants, the female parent has evolved a strategy to use its style microbiome to proactively defend the male gamete fertilization channel against future fungal attacks, thereby protecting the progeny and hence the genetic contribution of the mother. Specifically, *in vitro* and *in planta* experiments demonstrated that maize silks host bacteria that possess anti-*Fusarium* traits, perhaps selected independently by specific indigenous farmers in the Americas, at and distant from the site of maize diversification, though we cannot rule out that these bacteria originated later through horizontal transmission into silks. Confocal microscopy-based imaging suggests that one of these silk-associated bacteria protects the male gamete fertilization channel from *Fg* by a novel mechanism, specifically by colonizing wounds and trichomes (stigmatic hair), and forming biofilms on the silk surface before *Fg* infection; and later by colonizing and apparently killing *Fg* hyphae after infection. Overall, the results show that the microbiome of the female reproductive tissue of plants has the potential to defend the maternal parent and its progeny. Furthermore, this ability may have been inadvertently exploited by indigenous farmers, though this interpretation requires further evidence. Specifically, the origin of these bacteria needs investigation, in particular the extent to which they originate from seed via the maternal vascular tissue.

## Data availability statement

The original contributions presented in the study are included in the article/**Supplementary Material**. Further inquiries can be directed to the corresponding author.

## Author contributions

AS: Conceptualization, Formal Analysis, Investigation, Methodology, Validation, Visualization, Writing – original draft. VL-R: Investigation, Writing – review & editing. DB: Investigation, Writing – review & editing. MR: Conceptualization, Funding acquisition, Supervision, Writing – review & editing.

## Supplementary material

Supplementary Figure 1 A Maximum likelihood (ML) phylogenetic tree of the entire bacterial population based on unique operational taxonomic units (OTUs) (in black letters) and all the 201 bacterial strains cultured (strains ID in green letters). Bootstrap values are indicated above the branches.

Supplementary Figure 2 Predicted genus-level taxonomy of unpollinated silk-associated bacteria cultured from different host maize accessions spanning the Americas after growth in a common field. The predicted genera are based on full-length 16S Blastn searches at NCBI. The colours (yellow and dark green) highlight the total number of isolates (including redundant and unique sequences) belonging to the corresponding bacterial genus, arranged by host maize accession. The data is organized based on the driest to the wettest environments of source maize plants differentiated with different color codes (Dry, Moderate, and Wet) with the actual rainfall values (in mm) of the location from which the seeds were collected. The letter ‘H’ denotes a highland origin, ‘M’ denotes a mid-altitude origin, and ‘L’ denotes a lowland origin with respect to source maize plants. The latitudes and countries from where the seeds of host accessions were collected are presented. All plants were grown in Elora, Ontario, Canada.

Supplementary Figure 3 The most prevalent cultured unpollinated silk-associated bacteria at the OTU level from diverse American maize after growth in a common field. Full-length 16S RNA sequences sharing the same genus were multi-aligned, and each unique sequence was assigned a distinct OTU number (see Methods for stringency criteria). Shown are the resulting species- and OTU-level taxonomic predictions of silk-associated bacteria that were cultured from more than one host maize accession. The corresponding species prediction is noted along with the percentage identity to the top match at NCBI in brackets. The data is organized by the rainfall environment of source maize plants with the actual rainfall value (in mm) classified as Dry, Moderate, and Wet. The letter ‘H’ denotes a highland origin, ‘M’ denotes a mid-altitude origin, and ‘L’ denotes a lowland origin with respect to source maize plants. The latitude values and the country of origin of the source maize plants are noted. The most conserved OTUs are highlighted horizontally. All plants were grown in a common field in Elora, Ontario, Canada.

Supplementary Figure 4 Selection of safe anti-*Fg* bacterial strains from unpollinated silks for the replicated greenhouse trials. **(A)** Selection based on the initial human biosafety risk group assessments based on 16S-based phylogenetic tree reconstruction. 16S rRNA as well as whole genome sequencing were done for taxonomic identification of the unpollinated silk-associated anti-*Fg* bacterial strains. A BLAST search using the 16S rRNA sequences was done against the NCBI GenBank database that showed that AS112 resembled *Rouxiella chamberiensis* (99.34% identity match), and AS150 as *Pantoea ananatis* (99.41% identity match). The Gene bank accession identification for these strains are OL670733 and MW385266 respectively. The 16S rRNA-based neighbor-joining phylogenetic tree of these anti-*Fg* bacterial strains was constructed with these predicted taxonomies which confirmed that AS112 was closely related to *Rouxiella badensis* and AS150 to *Pantoea ananatis*. The Red arrows point to the anti-*Fusarium* bacterial strains used in the replicated greenhouse trials. The sequences other than the anti-*Fusarium* strains AS112 and AS150 were pooled from the gene bank which were closely related to these anti-*Fg* strains. These sequences were then aligned and evolutionary analyses were conducted in MEGA X to generate a neighbor-joining tree for all strains, with bootstrap values based on 900 replicates. **(B)** Selection based on clinical antibiotic disc susceptibility testing using a disc diffusion method. Anti-Fg bacterial strains were grown for two days in LB broth (pH 7.2) at 30°C, at 200 rpm. Then, a 500 µL liquid culture of each bacterial strain was spread evenly on a 150 mm X 15 mm plate containing Muller-Hilton agar. Ten antibiotic discs per plate were placed on the agar surface and pressed gently followed by incubation at 30°C for 24 h. Each antibiotic disc was tested in three different independent plates for each bacterial strain. The zones of inhibition were measured in mm and categorized as Susceptible (S): a larger ring of inhibition at >15mm diameter (green); Moderate (M): a smaller ring of inhibition (less than 15mm diameter) (yellow); Resistant (R): No ring of inhibition to the antibiotic disc used (red).
