# Supplementary material for: Bacteria existing in pre-pollinated styles (silks) can defend the exposed male gamete fertilization channel of maize against an environmental Fusarium pathogen
Source: Front Plant Sci. 2023 Dec 4;14:1292109. doi: 10.3389/fpls.2023.1292109 (PMC10726056; doi:10.3389/fpls.2023.1292109)
Supplement: Supplementary Figure 1 — A Maximum likelihood (ML) phylogenetic tree of the entire bacterial population based on unique operational taxonomic units (OTUs) (in black letters) and all the 201 bacterial strains cultured (strains ID in green letters). Bootstrap values are indicated above the branches. [file DataSheet_1.zip › Supplemental Methods.docx]

**Shrestha et al. – Supplemental Methods**

**Details for Bacteria Culturing, Glycerol Stock Preparation, Taxonomic Analysis**

### Operational Taxonomic Unit (OTU) Assignment, Phylogenetic Tree Construction,

### *In vitro* Assays for Anti-*Fusarium* Screening, and Whole Genome Sequencing and Genome mining

### Bacteria Culturing and Glycerol Stock Preparation

The frozen silk samples were allowed to thaw, followed by the addition of 600 µL of sterile 0.05 M sodium phosphate buffer (14.425 mL of 1M Na_2_HPO_4_, and 10.575 mL of 1M NaH_2_PO_4_, in a final volume of 500 mL of autoclaved ddH_2_O) at pH 7, followed by grinding in sterile mortars and pestles. The ground solution was then serially diluted with the same buffer, resulting in 1/10, 1/100, and 1/1000 dilutions. Then, 200 µL of each non-diluted sample and each dilution was plated onto 150 mm X 15 mm Petri plates containing 50 mL of Reasoner’s 2A (R2A) agar (R2A powder 18.12 g/L – containing 0.5 g Yeast extract, 0.5 g Proteose peptone, 0.5 g Casein hydrolysate, 0.5 g Glucose, 0.5 g Starch, 0.3 g Di-potassium phosphate, 0.024 g Magnesium sulphate, 0.3 g Sodium pyruvate, and 15 g Agar, per liter, adjusted to pH 7.2 with NaOH and autoclaved). . R2A is a nutrient-limited medium that was selected to allow the growth of slow-growing bacterial species, which would otherwise be crowded out by fast growers on more nutrient-rich media such as Luria-Bertani (LB) agar medium. These plates were labeled according to their host number and the date of plating, incubated at 30°C without Parafilming and checked after 3 days; unique colonies were restreaked onto new R2A agar plates. The plates were again incubated for 2 more additional days for a total of 5 incubation days to capture slow-growing colonies, which were also restreaked onto new R2A agar plates. A single pure colony from each restreaked R2A plate was then grown in a 3 mL liquid culture of LB (Luria-Bertani, composed of 10 g NaCl, 5 g yeast extract, and 10 g tryptone, per liter, adjusted to pH 7.2) and incubated at 30°C with shaking at 200 rpm for 1-2 days depending on the growth rate, whereas the original plate was Parafilmed and stored at 4°C. This switching of R2A later to LB liquid was for allowing the faster growth of the strains since LB is nutrient-rich medium.Subsequently, 400 µL of this liquid culture was added to 600 µL of 40% sterile glycerol which were made in pairs, in sterile screw-capped cryovials (Catalog#50000020, ThermoFisher, Canada), for storage at -80°C.

### Taxonomic Analysis

Two day old LB liquid cultures (1 mL each) were simultaneously used to extract bacterial genomic DNA for taxonomic analysis using the QIAamp DNA mini kits (catalog #51306, Qiagen, USA), following the manufacturer's protocol. Then 2 µL of each extracted DNA was quantified using a Qubit v1.2 fluorometer (Catalog #Q32857, Molecular Probes, Invitrogen by Life Technologies) followed by 16S rRNA PCR amplification and sequencing with the universal primers 27f (5'-AGAGTTTGATCMTGGCTCAG-3’) and 1492r(5'-GGTTACCTTGTTACGACTT-3’). A PCR master mix working solution was prepared by mixing 20 µL of GoTaq Green Master Mix (M712C, Promega, USA), 1 µL of 10 µM 27f primer, and 1 µL of 10 µM 1492r primer. To this, 100 ng of bacterial genomic DNA and Molecular Grade water were added to make a total final volume of 40 µL. The PCR amplification conditions were as follows: initial denaturation at 96 °C for 3 min, followed by 35 amplification cycles (94 °C for 30 sec, 48 °C for 30 sec, 72 °C for 90 sec), and a final extension at 72 °C for 7 min, using a PTC200 DNA Thermal Cycler (MJ Scientific, USA). Subsequently, the amplicon DNA was confirmed via gel electrophoresis, purified using the GFX PCR DNA purification kit (Fisher, Catalog#45001489, GE Healthcare), and re-quantified using a Qubit v1.2 fluorometer (Catalog #Q32857, Molecular Probes, Invitrogen by Life technologies). These samples were then proceeded for PCR cycle sequencing, the procedure of which was as follows: template DNA (28 ng per 1 kb) was added to the bottom of the wells of a 96-well PCR plate, which was then placed on a heat block at 96 °C for 10 min until the DNA was dried completely. A master mix was prepared by mixing 1 µL BigDye (v.3.1) terminator mix, 2 µL 5X SeqBuffer, 8 µL Molecular Grade Water, and 1 µL of 10 pmol/µL of each primer [27f primer (5'-AGAGTTTGATCMTGGCTCAG-3’) and 1492r primer (5'-GGTTACCTTGTTACGACTT-3’)]; mixed well and then this Master Mix was added to the dried DNA samples (12 µL per well) on the same PCR plate. The PCR plate was sealed and vortexed thoroughly to resuspend the DNA with Master Mix, followed by brief centrifugation. The plate was thermocycled under the following conditions: initial denaturation at 96 °C for 2 min, followed by 30 amplification cycles (96 °C for 30 sec, 50° C for 15 sec, 60° C for 4 min), using a PTC200 DNA Thermal Cycler (MJ Scientific, USA). The samples were then submitted for sequencing at the Department of Molecular and Cellular Biology Genomics Facility at the University of Guelph. All 16S DNA sequences received were first trimmed and aligned to construct contigs using BioEdit software (Hall, 2011). Only one 27F or 1492R sequence was used if contigs could not be constructed due to a large number of ‘N’ undetermined nucleotides. Nucleotide BLAST searches of the sequences were performed against the 16S ribosomal RNA sequence (Bacteria and Archaea) database, optimized for Blastn at NCBI to obtain taxonomic predictions. The highest percent identity matches, with the higher query coverage, were counted as the top matches and recorded. The query coverage percentage reported was greater than 95%.

### Operational Taxonomic Unit (OTU) Assignment and Phylogenetic Tree Construction

The ClustalW multiple sequence DNA alignment tool (Madeira et al., 2019) was used to multi-align the 16S RNA sequences that share the same genus. The objective of this step was to assign each strain to a unique Operational Taxonomic Unit (OTU). Those sequences that were mismatched within 10 bp of the 5’ and 3’ termini were not counted as differences. In addition, mismatches of a nucleotide base to an ‘N’ were also not counted as a difference. Based on these criteria set, a few of the OTUs represent more than one species. The longest high-quality sequence of all the sequences within an OTU group with the least number of ‘Ns’, was selected to represent that specific OTU group. The silk phylogenetic tree was constructed based on the 62 unique OTUs representing the complete culturable silk library. MEGA-X software (Kumar et al., 2018) was used to generate a maximum likelihood (ML) phylogenetic tree with bootstrapping of 900 replicates, where 16S sequences were first trimmed and edited using BioEdit software (Hall, 2011), and the sequences were aligned with the Muscle tool from MEGA-X.

### *In vitro* Assays for Anti-*Fusarium* Screening

To screen silk-associated bacteria for *in vitro* inhibition of the growth of *Fusarium graminearum* (*Fg*), dual culture assays were conducted as previously described by (Mousa et al., 2015). The *Fg* strain, *Fg*MT#1, was isolated in the Raizada Lab from *Fg*-contaminated corn seeds from Southwestern Ontario in the Summer of 2018, which was received from the Ridgetown Campus, University of Guelph. For the assays, *Fg* from a fresh PDA plate was inoculated into 10 mL of sterile potato dextrose media (PDB) (Catalog # DF0549-17-9, Fisher Scientific, Canada), incubated for 72 hours at 25 °C with shaking at 120 rpm. After 3 days, sterile ceramic beads were added to the Falcon tube, and vortexed for 3 min to break the large mycelial mass and create a uniform *Fg* suspension. One mL of this *Fg* mycelial suspension was then added into 100 mL of sterile, melted, cooled (to 41°C) potato dextrose agar (PDA) media, which was then swirled to mix evenly, and then 50 mL poured into 150-mm diameter Petri dishes which were allowed to solidify. Two-day-old cultures of silk-associated bacterial isolates were used for the assay. They were grown in an LB liquid broth (pH 7.2) for 2 days at 30 °C with shaking at 200 rpm. The bacterial liquid cultures were then centrifuged for 10 min, followed by resuspension in LB liquid media to an optical density (OD_600_) of 0.4-0.6. Once the *Fg-*embedded PDA plates were solidified, seven wells (1 cm diameter/well) were punched out using sterile glass tubes, three for controls and four for bacterial isolates. 100 µL silk-associated bacterial liquid cultures were pipetted into each well for co-culturing. These plates were then incubated at 25 °C for 48 hours, after which the diameter of each zone of inhibition was measured (in cm) and pictures of the plates were taken. The two positive controls used were a commercial foliar fungicide, PROLINE® (registration #28359, Bayer, Calgary, AB, Canada) at a concentration of 1:10 Proline: ddH_2_O, and a known anti-*Fg* bacterial endophyte from the Raizada lab, M6 (*Enterobacter* spp-OD_600_ of 0.4-0.6). The negative control was LB liquid media (pH 7.2). The plates were made in triplicate, each treated as an independent replicate, where each bacterium was inoculated randomly between plates. For analyzing the results of these assays, the diameter of each *Fg*-inhibition zone was modeled with a generalized linear mixed model (GLMM) using PROC GLIMMIX, then analyzed using a Gaussian distribution, and compared using Tukey’s pairwise comparison in SAS 9.4 (SAS Institute, Cary, NC) with a significance level of P≤0.05. However, while reporting here, each silk-associated anti-*Fg* bacteria was compared only with the negative control (LB Control) and the positive control (Proline, the commercial fungicide) treatments.

### Whole Genome Sequencing and Genome Mining

Two-day-old liquid cultures of the anti-*Fg* bacterial isolates (grown for 48 hours at 30 °C with shaking at 200 rpm) were used to extract DNA using a DNeasy®UltraClean Microbial Kit (Catalog # 10196-4, Qiagen, USA) following the manufacturer’s protocol. The DNA samples were then submitted to the Microbial Genome Sequencing Center (Pittsburgh, Pennsylvania, USA) for whole genome sequencing using Illumina (NextSeq 2000 platform). At MiGS, quality control, and adapter trimming were undertaken with bcl2fastq (version 2.20.0.445, default parameters) (Illumina, 2022). Species-level taxonomy were undertaken using MetaPhlAn3 (Tool: 3.0.7, December 9, 2020, database version; default parameters + ‘add_viruses) (Beghini et al., 2021). The short read mode of Unicycler (version 0.4.8, default parameters) (Wick et al., 2017) was used as a SPAdes optimizer (Prjibelski et al., 2020) to generate short-read assemblies. Gene annotation employed PGAP (Tool: Build5132, database version January 11, 2021, using default parameters) (Seemann, 2014) and prokka (tool and database version: 1.14.5; using default parameters + ‘—rnammer’ + ‘—rfam’, added ‘—metagenome’ when processing metagenomic/unclassified samples, added ‘—kingdom Viruses’ when processing viral & bacteriophage samples) (Feldgarden et al., 2019). The gene annotation files for the bacterial isolates were compiled into an excel file and the genes of interest were searched for using acronyms of gene names.

### Greenhouse Trials to Test the Ability of Silk-Associated Bacterial Strains to Suppress Gibberella Ear Rot (GER) Disease

**Microbial Treatment Selection: Antibiotic Susceptibility Testing**

To select strains for the greenhouse trials, the anti-*Fg* silk-associated bacterial strains were further tested for antibiotic susceptibility using a disc diffusion assay. Twenty different antibiotic discs were tested against the anti-*Fusarium* strains to examine if they were resistant or susceptible. The antibiotic discs used were: LEV_5_ – Levofloxacin (5 µg/mL), FOX_30_ – Cefoxitin (30 µg/mL), RD_5_ – Rifampicin (5 µg/mL), CIP_5_ – Ciprofloxacin 5 µg/mL), VA_30_ – Vancomycin (30 µg/mL), S_10_ – Streptomycin (10 µg/mL), TE_30_ – Tetracycline (30 µg/mL), TGC_15_ – Tigecycline (15 µg/mL), AMP_10_ – Ampicillin (10 µg/mL), K_30_ – Kanamycin (30 µg/mL), C_30_ – Chloramphenicol (30 µg/mL), E_5_ – Erythromycin (5 µg/mL), TEC_30_ – Teicoplanin (30 µg/mL), FD_10_ – Fusidic Acid (10 µg/mL), FCA_25_ – Fluconazole (25 µg/mL), MEL_10_ – Mecillinam (10 µg/mL), CAZ_10_ – Ceftazidime (10 µg/mL), NV_30_ – Novobiocin (30 µg/mL), NA_30_ – Nalidixic acid (30 µg/mL), and W_5_ – Trimethoprim (5 µg/mL).

Each bacterial culture (500 µL) at OD_600_ 0.4-0.7 (grown for 2 days at 30 °C, 200 rpm) was added onto each Petri dish (150 mm X 15 mm) containing 50 mL of Mueller-Hilton agar media (Catalog No. OXCM0337B, Fisher Scientific, Canada). The culture on the agar plate was spread evenly using a sterile plate spreader and let dry for 5 min. Then, ten antibiotic discs were placed on the agar surface of each plate, evenly spaced, and pressed gently with sterile forceps. The plates were checked after 24 h of incubation at 30 °C and the zones of inhibition were measured with a clear ruler. Each antibiotic disc was tested in three different independent plates for each bacterium.

**Greenhouse Trials**

**Seedling Preparation and Transplanting**

Commercial hybrid maize DKC55-05RIB (Bayer Crop Science, Canada), moderately susceptible to *Fg,* was selected. Seeds were surface sterilized with 0.1% Triton X-100 detergent for 10 min with shaking, decanted, and then 3% of sodium hypochlorite was added for 10 min, rinsed with sterile distilled water, then washed with 95% ethanol for 10 min, concluding with 5-6 times rinses with autoclaved, distilled water. The sterilization process was verified by plating the last wash on LB and PDA plates at 30 °C for 2 days.

These seeds were germinated on medium-sized moistened vermiculite. A black tray with holes at the bottom was used. Below this, a white tray was kept to hold water. The tray was covered with a clear plastic lid and kept in the dark for 5 days, followed by placing it under the light in the lab for additional 36 hours before moving to the greenhouse. The trays with seedlings were left in the greenhouse for the whole day until being transplanted in the late afternoon. Uniformly germinated seedlings (3 seedlings/pot) were transplanted into 20 L pots containing Turface clay (Turface Athletics Inc, USA). These pots were disinfected and holes were drilled on the bottom and sides before filling with Turface. To avoid sunburn and to allow the seedlings to adjust to the light for 1-2 days, the shade curtains were drawn across the greenhouse zone before allowing them to grow under full sunlight. Seedling thinning was done after 2 weeks, and one plant was left per pot.

**Plant Growth Conditions and Experimental Design**

There were two independent greenhouse trials; the second trial was started 2 weeks after the first one. Both the greenhouse zones used a randomized complete block design (RCBD) with seven treatments in 6 blocks; each treatment group consisted of 24 plants in total (4 treatment replicates in each block). There were seven treatments (two silk-associated bacterial candidates, a negative and positive control, along with three non-silk-associated bacterial candidates to be reported in a separate manuscript). A drip irrigation system at an injector rate of 1:200 with 100 PPM Nitrogen Plant-Prod 24-10-20 fertilizer (Product Number: 10535, Brampton, Ontario, Canada) was installed with an additional 1.746 g/L MgSO_4_. In addition, 1 tablespoon of limestone was added directly onto the pots every week and then the plants were watered to mix the powder with the Turface. An issue was raised of having a very low pH in the irrigation water supplied via drip lines, which was solved by adding 20 g/L of KHCO_3_ to the fertilizer mixture. The plants in the greenhouse were grown under natural sunlight; and to ensure the plants were getting enough light (≥800 µmol m^-2^s^-1^), light levels were measured both at the canopy and pot level using a Quantum photosynthetically active radiation (PAR) light meter (Apogee Instruments, USA). All pots in the greenhouse zone were rotated from block to block to ensure that the plants were receiving uniform light levels. Regular airflow in the greenhouse zones was supplied via evaporative cooler vents. Yellow insect traps, as well as AMBLYforceS (BeneficialInsectary, USA), were used to prevent aphids and thrip infestation in the greenhouse zones.

**Self/Sibling Pollination**

Maize plants were regularly monitored for flowering, which started 46 to 50 days after sowing. Approximately, 48 h after silk emergence, deliberate self/sibling pollination was done in the mornings, for which tassels were bagged the prior day in the late afternoons to collect pollen for pollination. The pollinated ears were then marked, followed by waiting for 48 h to permit fertilization. For this study, only the primary ears were chosen for pollination and treatment application.

####

### Bacterial Fluorescent Tagging and Confocal Scanning Fluorescence Microscopy

#### Fluorescent Tagged *Fg* Strain

A green fluorescent protein (GFP)-tagged *Fg* strain (ZTE-2A) (Miller et al., 2004) was obtained, courtesy of R. Proctor, National Center for Agricultural Utilization Research, Agricultural Research Service, U.S. Department of Agriculture, Peoria, IL, USA. The strain was in a GZ3639 genetic background. GZ3639 was initially isolated from scabby wheat in the Midwest United States (Lutz et al., 2003). *Fg* ZTE-2A inoculum was prepared by inoculating the strain in 25 mL of PDB in a 50 mL Falcon tube, followed by shaking at 120 rpm at 25°C for 72 h.

#### AS112 Competent Cell Preparation

Twenty-five milliliters of LB broth (pH 7.2) was inoculated with 250 µL of the overnight liquid culture (prepared from a single AS112 bacterial colony from a re-streaked plate, grown at 30°C at 200 rpm) and incubated until the early log phase (OD_600_ = 0.2 to 0.3). The bacterial cells were then harvested by chilling for 15-20 min on ice and centrifuged at 4000 x *g* for 15 min at 4°C. After removing the supernatant, the pellets were re-suspended in ice-cold water (half the original amount), pipetted up, and down to mix thoroughly, and centrifuged as mentioned earlier; these steps were repeated 3 times. Then, the pellets were re-suspended in 5 mL of ice-cold 10% glycerol, centrifuged, and finally re-suspended in 100 µL of 10% ice-cold glycerol from which 50 µL aliquots were made and frozen in liquid nitrogen and stored at -80°C.

#### AS112 DsRed Plasmid Transformation

A broad-host promoter plasmid, pSW002-PpsbA-DsRed-Express2, was obtained (Catalog #111257, Addgene, USA) and stored at -80°C. The plasmid was extracted following a standard protocol using the Quantum Prep® Plasmid miniprep kit (catalog #732-6100, Bio-Rad Laboratories, Inc., USA), then introduced into competent AS112 bacterial cells by electroporation. Suspensions of 40 µL chilled AS112 competent cells were mixed with 2 µL of plasmid DNA, kept chilled in ice for one minute, then electroporated at 1.6 kV for 1 sec using a Bio-Rad Gene Pulser 200/2.0 (Bio-Rad Hercules, USA). Immediately after electroporation, the cells were incubated for 2-3 h in 1 mL of pre-warmed LB broth (pH 7.2) with shaking at 200 rpm. The transformed cells (50 µL) were plated on LB agar containing tetracycline (5 mg/mL) and incubated for 24 h at 30°C, then the plate was examined for fluorescent colonies (Illumatool, Catalog# LR 92240, LightTools Research, USA). A single fluorescent colony from this plate was restreaked onto a new LB plate containing tetracycline (5 mg/mL). A single colony grown on this new plate was used to prepare a liquid culture and glycerol stocks for future use.

**References**

Beghini, F., Mciver, L. J., Blanco-Míguez, A., 1​, ​, Dubois, L., Asnicar, F., et al. (2021). Integrating taxonomic, functional, and strain-level profiling of diverse microbial communities with bioBakery 3. *Elife*. 10, e65088. doi: 10.7554/eLife.65088.

Feldgarden, M., Brover, V., Haft, D. H., Prasad, A. B., Slotta, D. J., Tolstoy, I., et al. (2019). Validating the AMRFinder tool and resistance gene database by using antimicrobial resistance genotype-phenotype correlations in a collection of isolates. *Antimicrob. Agents Chemother*. 63, e00483-19. doi: 10.1128/AAC.00483-19.

Hall, T. (2011). BioEdit: An important software for molecular biology. *GERF Bull. Biosci.* 2, 60–61.

Illumina (2022). bcl2fastq: A proprietary illumina software for the conversion of bcl files to basecalls. Available at: https://support.illumina.com/sequencing/sequencing_software/bcl2fastq-conversion-software.html.

Kumar, S., Stecher, G., Li, M., Knyaz, C., and Tamura, K. (2018). MEGA X: Molecular Evolutionary Genetics Analysis across computing platforms. *Mol. Biol. Evol.* 35, 1547. doi: 10.1093/MOLBEV/MSY096.

Madeira, F., Park, Y. M., Lee, J., Buso, N., Gur, T., Madhusoodanan, N., et al. (2019). The EMBL-EBI search and sequence analysis tools APIs in 2019. *Nucleic Acids Res.* 47, W636–W641. doi: 10.1093/nar/gkz268.

Miller, S. S., Chabot, D. M. P., Ouellet, T., Harris, L. J., and Fedak, G. (2004). Use of a *Fusarium graminearum* strain transformed with green fluorescent protein to study infection in wheat (*Triticum aestivum*). *Can. J. Plant Pathol.* 26, 453–463. doi: 10.1080/07060660409507165.

Mousa, W. K., Shearer, C. R., Limay-Rios, V., Zhou, T., and Raizada, M. N. (2015). Bacterial endophytes from wild maize suppress *Fusarium graminearum* in modern maize and inhibit mycotoxin accumulation. *Front. Plant Sci.* 6, 805. doi: 10.3389/fpls.2015.00805.

Prjibelski, A., Antipov, D., Meleshko, D., Lapidus, A., and Korobeynikov, A. (2020). Using SPAdes De Novo Assembler. *Curr. Protoc. Bioinforma.* 70, e102. doi: 10.1002/CPBI.102.

Seemann, T. (2014). Genome analysis Prokka: rapid prokaryotic genome annotation. *Bioinformatic Appl. Note* 30, 2068–2069. doi: 10.1093/bioinformatics/btu153.

Wick, R. R., Judd, L. M., Gorrie, C. L., and Holt, K. E. (2017). Unicycler: Resolving bacterial genome assemblies from short and long sequencing reads. *PLOS Comput. Biol.* 13, e1005595. doi: 10.1371/JOURNAL.PCBI.1005595.
