# Supplementary material for: Bacteria existing in pre-pollinated styles (silks) can defend the exposed male gamete fertilization channel of maize against an environmental Fusarium pathogen
Source: Front Plant Sci. 2023 Dec 4;14:1292109. doi: 10.3389/fpls.2023.1292109 (PMC10726056; doi:10.3389/fpls.2023.1292109)
Supplement: Supplementary Figure 1 — A Maximum likelihood (ML) phylogenetic tree of the entire bacterial population based on unique operational taxonomic units (OTUs) (in black letters) and all the 201 bacterial strains cultured (strains ID in green letters). Bootstrap values are indicated above the branches. [file DataSheet_1.zip › Supplemental Results and Discussion.docx]

**Shrestha et al.-Supplemental Results and Discussion**

###

### Result

### Genome Mining of Silk-Associated Anti-*Fusarium* Bacterial Isolates

To gain a better understanding of candidate antifungal mechanisms of action, whole genome sequencing of the 6 bacterial isolates was conducted that showed strong suppression of *F. graminearum* *in vitro,* followed by genome mining guided by the literature (Table 2). The genome mining results demonstrated the presence of the anti-*Fusarium* gene *phzF,* responsible for phenazine biosynthesis, in all of the strong anti-*Fg* bacterial isolates; phenazine is well known to have broad-spectrum anti-pathogen and anti-fungal activities (Chin-A-Woeng et al., 2003; Pierson and Pierson, 2010) and to promote induced systemic resistance (ISR) in plants (De Vleesschauwer et al., 2007). Furthermore, 4 of the 6 bacterial isolates (except AS89 and AS501) were endowed with genes encoding chitosanase/chitinase; these enzymes can inhibit the growth of phytopathogenic fungi by degrading cell walls, leading to severe damage and even pathogen death (Dimkić et al., 2022). Interestingly, the two isolates missing chitinase encoded nitronate monooxygenase, an enzyme that is reported to have a role in mitigating nitro-oxidative cellular damage and maintaining redox balance under stress conditions (Marroquin-Guzman et al., 2017). Additionally, except for strain AS89, the genomes of all of the potent isolates encoded the enzyme, acetolactate decarboxylase, responsible for the biosynthesis of the signaling compound acetoin (Tuttobene et al., 2019), known to trigger ISR in host plants against plant pathogens (Liang et al., 2022). However, other genes that biosynthesize known anti-*Fusarium* compounds were not found in the genome of these bacterial strains, specifically 2, 3-butanediol (Liang et al., 2022), colicin V (Mousa et al., 2016); and others (iturin, fengycin, bacillomycin) (Mora et al., 2015).

**Discussion**

**Preliminary Genome Mining Suggesting the Potential Mechanisms of Anti-*Fg* strains**

Regarding the mechanisms underlying the *in vitro* anti-*Fg* activities of the six potent strains in this study, the genomes of all six strains possessed similar genes like *phzF*, responsible for the biosynthesis of phenazine, a nitrogen-containing aromatic compound produced by bacteria, that exhibits potent antifungal activities that lead to the accumulation of toxic oxygen radicals (Dietrich et al., 2008; Mazurier et al., 2009). Zhou et al. (2016) reported that phenazine derivatives produced by *Pseudomonas* spp. contribute to the suppression of *Fg* mycelial growth as well as that of other phytopathogenic fungi. Likewise, Sun et al. (2021) showed *Pseudomonas aeruginosa* strain NF011 produces a phenazine compound that completely inhibits *Fg* spore germination and damages *Fg* hyphal vacuoles, suggesting that this strain could effectively prevent *Fg* spores from infecting wheat heads and grains, and suppress the production of DON mycotoxin. However, a more recent study has shown that phenazines may have diverse modes of action against *Fusarium.* For example, a phenazine secreted by *Pseudomonas piscium* strain ZJU60 was shown to directly target the activity of *Fusarium graminearum* protein *Fg*Fcn5, leading to the deregulation of histone acetylation in *Fg* and suppression of hyphal growth, virulence, and DON mycotoxin biosynthesis (Chen et al., 2018).

Except for AS89, all the other strong anti-*Fg* strains in this study contained genes responsible for the biosynthesis of acetoin, a hormone known to trigger induced systemic resistance (ISR) in host plants and protect them against pathogens (Chowdhury et al., 2015). For example, a study by Rudrappa et al. (2010) reported that acetoin secreted by *Bacillus subtilis* can trigger ISR in *Arabidopsis thaliana* to help suppress the pathogen *Pseudomonas syringae.* In the future, it will interesting to identify whether strain AS112 uses acetoin and/or other compounds to signal to host silk cells, given that it has the ability to colonize the silk trichome (stigmatic hair) and epidermis, and heavily colonize silk wound sites, suggestive of an intimate association between host and microbe.

Furthermore, four of the six potent anti-*Fg* strains also encode the enzyme chitinase, which has been demonstrated to successfully inhibit the growth of phytopathogenic fungi by hydrolyzing the glycosidic and peptide bonds of chitin, a principle component of the fungal cell wall, leading to cell lysis, severe damage, and even pathogen death (Dimkić et al., 2022). In addition, the hydrolysis of chitin produces N-acetyl glucosamine which also has been reported to induce plant host resistance (Gohel et al., 2016). Beyond these well-known mechanisms, the chitinase and chitosanase-producing endophytic fungus *Sphaerodes mycoparasitica* has been shown to absorb aurofusarin, a notable *Fusarium graminearum* mycotoxin, from attacked *Fusarium* cells, by lysing chitin, implying its ability to detoxify or neutralize this mycotoxin (Vujanovic and Goh, 2011). Chitinase-producing bacteria have been reported as biocontrol agents for different fungal diseases in plants. Examples include *B. cereus* against *F. verticillioides* in maize (Morales-Ruiz et al., 2021); *B. subtilis* against *F. graminearum* in wheat (Mahmoud, 2016); *B. pumilis* against *F. graminearum* and *Bipolaris sorokiniana* in wheat (Dimkić et al., 2022); *B. thuringiensis* against *Fusarium oxysporum* and *Sclerotium rolfsii* in soybean (Reyes-Ramírez et al., 2004; Lopes et al., 2017); *B. subtilis* against *F. oxysporum* in yam (Veliz et al., 2017); *Bacillus cereus* against *Rhizoctonia solani* in cotton (Gupta et al., 2006) and *Pseudomonas aeruginosa* against *Sclerotinia sclerotiorum* in peanuts (Gupta et al., 2006). Indeed, chitinase production has been identified as the main biocontrol mechanism in some studies (Veliz et al., 2017).

Interestingly, the two potent anti-*Fg* strains in this study that appear to lack the chitinase gene (AS89 and AS501) contain nitronate monooxygenase (NMO) genes, responsible for the mitigation of nitro-oxidative cellular damage under stress conditions (Marroquin-Guzman et al., 2017). For example, in the fungus *Magnaporthe oryzae*, the expression of NMO-encoded nitronate monooxygenases was reported to be associated with self-detoxification and suppression of the rice immune response (Marroquin-Guzman et al., 2017). Additionally, Pan et al. (2018) reported that the upregulation of the wheat NMO was correlated with the *Fg* infection level, suggesting that NMO has a potential role in the wheat immune response. It has been reported that the Type III secretion systems facilitate the secretion of effectors that suppress host defense, to promote stable colonization (Zboralski et al., 2022).

**References**

Chen, Y., Wang, J., Yang, N., Wen, Z., Sun, X., Chai, Y., et al. (2018). Wheat microbiome bacteria can reduce virulence of a plant pathogenic fungus by altering histone acetylation. *Nat. Commun.* 9, 3429. doi: 10.1038/s41467-018-05683-7.

Chin-A-Woeng, T. F. C., Bloemberg, G. V., and Lugtenberg, B. J. J. (2003). Phenazines and their role in biocontrol by *Pseudomonas* bacteria. *New Phytol.* 157, 503–523. doi: 10.1046/J.1469-8137.2003.00686.X.

Chowdhury, S. P., Hartmann, A., Gao, X., and Borriss, R. (2015). Biocontrol mechanism by root-associated *Bacillus amyloliquefaciens* FZB42 – a review. *Front. Microbiol.* 6, 780. doi: 10.3389/fmicb.2015.00780.

De Vleesschauwer, D., Cornelis, P., and Höfte, M. (2007). Redox-active pyocyanin secreted by *Pseudomonas aeruginosa* 7NSK2 triggers systemic resistance to *Magnaporthe grisea* but enhances *Rhizoctonia solani* susceptibility in rice. *MPMI* 19, 1406–1419. doi: 10.1094/MPMI-19-1406.

Dietrich, L. E. P., Teal, T. K., Price-Whelan, A., and Newman, D. K. (2008). Redox-active antibiotics control gene expression and community behavior in divergent bacteria. *Science (80-. ).* 321, 1203. doi: 10.1126/science.1160619.

Dimkić, I., Janakiev, T., Petrović, M., Degrassi, G., and Fira, D. (2022). Plant-associated *Bacillus* and *Pseudomonas* antimicrobial activities in plant disease suppression via biological control mechanisms - A review. *Physiol. Mol. Plant Pathol.* 117, 101754. doi: 10.1016/J.PMPP.2021.101754.

Gohel, V., Singh, A., Vimal, M., Ashwini, P., and Chhatpar, H. (2016). Bioprospecting and antifungal potential of chitinolytic microorganisms. *African J. Biotechnol.* 5, 54–72. doi: 10.4314/ajb.v5i2.

Gupta, C. P., Kumar, B., Dubey, R. C., and Maheshwari, D. K. (2006). Chitinase-mediated destructive antagonistic potential of *Pseudomonas aeruginosa* GRC1 against *Sclerotinia sclerotiorum* causing stem rot of peanut. *BioControl* 51, 821–835. doi: 10.1007/S10526-006-9000-1.

Liang, L., Fu, Y., Deng, S., Wu, Y., and Gao, M. (2022). Genomic, antimicrobial, and aphicidal traits of *Bacillus velezensis* ATR2, and its biocontrol potential against ginger rhizome rot disease caused by *Bacillus pumilus*. *Microorganisms* 10, 63. doi: 10.3390/MICROORGANISMS10010063/S1.

Lopes, R., Cerdeira, L., Tavares, G. S., Ruiz, J. C., Blom, J., Horácio, E. C. A., et al. (2017). Genome analysis reveals insights of the endophytic *Bacillus toyonensis* BAC3151 as a potentially novel agent for biocontrol of plant pathogens. *World J. Microbiol. Biotechnol.* 33, 185. doi: 10.1007/S11274-017-2347-x.

Mahmoud, A. F. (2016). Genetic variation and biological control of *Fusarium graminearum* isolated from wheat in Assiut-Egypt. *Plant Pathol. J.* 32, 145. doi: 10.5423/PPJ.OA.09.2015.0201.

Marroquin-Guzman, M., Hartline, D., Wright, J. D., Elowsky, C., Bourret, T. J., and Wilson, R. A. (2017). The *Magnaporthe oryzae* nitrooxidative stress response suppresses rice innate immunity during blast disease. *Nat. Microbiol.* 2, 17054. doi: 10.1038/NMICROBIOL.2017.54.

Mazurier, S., Corberand, T., Lemanceau, P., and Raaijmakers, J. M. (2009). Phenazine antibiotics produced by fluorescent pseudomonads contribute to natural soil suppressiveness to Fusarium wilt. *Int. Soc. Microb. Ecol.* 3, 977–991. doi: doi.org/10.1038/ismej.2009.33.

Mora, I., Cabrefiga, J., and Montesinos, E. (2015). Cyclic lipopeptide biosynthetic genes and products, and inhibitory activity of plant-associated *Bacillus* against phytopathogenic bacteria. *PLoS One* 10, e0127738. doi: 10.1371/JOURNAL.PONE.0127738.

Morales-Ruiz, E., Priego-Rivera, R., Figueroa-López, A. M., Cazares-Álvarez, J. E., and Maldonado-Mendoza, I. E. (2021). Biochemical characterization of two chitinases from *Bacillus cereus* sensu lato B25 with antifungal activity against *Fusarium verticillioides* P03. *FEMS Microbiol. Lett.* 368, 218. doi: 10.1093/FEMSLE/FNAA218.

Mousa, W. K., Schwan, A. L., and Raizada, M. N. (2016). Characterization of antifungal natural products isolated from endophytic fungi of finger millet (*Eleusine coracana*). *Molecules* 21, 1171. doi: 10.3390/molecules21091171.

Pan, Y., Liu, Z., Rocheleau, H., Fauteux, F., Wang, Y., McCartney, C., et al. (2018). Transcriptome dynamics associated with resistance and susceptibility against Fusarium head blight in four wheat genotypes. *BMC Genomics* 19, 642. doi: 10.1186/S12864-018-5012-3.

Pierson, L. S., and Pierson, E. A. (2010). Metabolism and function of phenazines in bacteria: Impacts on the behavior of bacteria in the environment and biotechnological processes. *Appl. Microbiol. Biotechnol.* 86, 1659–1670. doi: 10.1007/S00253-010-2509-3/FIGURES/3.

Reyes-Ramírez, A., Escudero-Abarca, B. I., Aguilar-Uscanga, G., Hayward-Jones, P. M., and Eleazar Barboza-Corona, J. (2004). Antifungal activity of *Bacillus thuringiensis* chitinase and its potential for the biocontrol of phytopathogenic fungi in soybean seeds. *J. Food Sci.* 69, M131–M134. doi: 10.1111/J.1365-2621.2004.TB10721.X.

Rudrappa, T., Biedrzycki, M. L., Kunjeti, S. G., Donofrio, N. M., Czymmek, K. J., Paré, P. W., et al. (2010). The rhizobacterial elicitor acetoin induces systemic resistance in *Arabidopsis thaliana*. *Commun. Integr. Biol.* 3, 130. doi: 10.4161/CIB.3.2.10584.

Sun, X., Xu, Y., Chen, L., Jin, X., and Ni, H. (2021). The salt-tolerant phenazine-1-carboxamide-producing bacterium *Pseudomonas aeruginosa* NF011 isolated from wheat rhizosphere soil in dry farmland with antagonism against *Fusarium graminearum*. *Microbiol. Res.* 245, 126673. doi: 10.1016/j.micres.2020.126673.

Tuttobene, M. R., Fernández-García, L., Blasco, L., Cribb, P., Ambroa, A., Müller, G. L., et al. (2019). Quorum and light signals modulate acetoin/butanediol catabolism in *Acinetobacter* spp. *Front. Microbiol.* 10, 1376. doi: 10.3389/FMICB.2019.01376/BIBTEX.

Veliz, E. A., Martínez-Hidalgo, P., and Hirsch, A. M. (2017). Chitinase-producing bacteria and their role in biocontrol. *AIMS Microbiol.* 3, 689. doi: 10.3934/MICROBIOL.2017.3.689.

Vujanovic, V., and Goh, Y. K. (2011). *Sphaerodes mycoparasitica* biotrophic mycoparasite of 3-acetyldeoxynivalenol- and 15-acetyldeoxynivalenol-producing toxigenic *Fusarium graminearum* chemotypes. *FEMS Microbiol. Lett.* 316, 136–143. doi: 10.1111/J.1574-6968.2010.02201.X.

Zboralski, A., Biessy, A., and Filion, M. (2022). Bridging the gap: Type III secretion systems in plant-beneficial bacteria. *Microorganisms* 10, 187. doi: 10.3390/microorganisms10010187.

Zhou, L., Jiang, H.-X., Sun, S., Yang, D.-D., Jin, K.-M., Zhang, W., et al. (2016). Biotechnological potential of a rhizosphere *Pseudomonas aeruginosa* strain producing phenazine-1-carboxylic acid and phenazine-1-carboxamide. *World J. Microbiol. Biotechnol.* 32, 50. doi: DOI 10.1007/s11274-015-1987-y.
