# Supplementary material for: Bacteria existing in pre-pollinated styles (silks) can defend the exposed male gamete fertilization channel of maize against an environmental Fusarium pathogen
Source: Front Plant Sci. 2023 Dec 4;14:1292109. doi: 10.3389/fpls.2023.1292109 (PMC10726056; doi:10.3389/fpls.2023.1292109)
Supplement: Supplementary Figure 1 — A Maximum likelihood (ML) phylogenetic tree of the entire bacterial population based on unique operational taxonomic units (OTUs) (in black letters) and all the 201 bacterial strains cultured (strains ID in green letters). Bootstrap values are indicated above the branches. [file DataSheet_1.zip › Tables S2-S5.docx]

Supplementary Table 2. A complete master list of unpollinated silk-associated bacterial strains isolated from different host maize accessions with their closest taxonomic identities based on full-length 16S RNA sequence BLAST searches in Genbank. The 16S RNA sequences are deposited in Genbank and are retrievable by the respective accession number. Also shown are the dual culture assay results, describing the effect of each bacterial strain on the growth or colour of *Fusarium graminearum* *in vitro*.

| **Host** | **Sample Name** | Strain ID | **OTUs No.** | **Predicted genus** | **Predicted species** | **% Query cover** | **% identity** | **Accession ID** | **Dual culture assay** |
| --- | --- | --- | --- | --- | --- | --- | --- | --- | --- |
| Camelia | R1-19S-L | AS158 | OTU1 | *Acidovorax* | *wautersii* | 99 | 99.50% | MW369871 |  |
| Camelia | R1-19S-V | AS165 | OTU1 | *Acidovorax* | *wautersii* | 100 | 99.57% | MW369878 | Color change |
| Camelia | R1-19S-A2 | AS-183 | OTU1 | *Acidovorax* | *wautersii* | 100 | 99.85% | OL670762 |  |
| Camelia | R1-19S-A | AS153 | OTU10 | *Microbacterium* | *testaceum* | 99 | 99.13% | MW369867 | Biofilm |
| Camelia | R1-19S-K | AS157 | OTU10 | *Microbacterium* | *testaceum* | 99 | 99.13% | MW369870 |  |
| Camelia | R1-19S-N | AS159 | OTU10 | *Microbacterium* | *testaceum* | 100 | 99.35% | MW369872 |  |
| Camelia | R1-19S-A6 | AS-187 | OTU10 | *Microbacterium* | *testaceum* | 100 | 99.71% | OL670765 |  |
| Camelia | R1-19S-B | AS-145 | OTU15 | *Pantoea* | *agglomerans* | 100 | 99.44% | OL672163 | No effect |
| Camelia | R1-19S-Z | AS-166 | OTU15 | *Pantoea* | *agglomerans* | 100 | 99.43% | OL672165 | No effect |
| Camelia | R1-19S-C | AS146 | OTU17 | *Pantoea* | *ananatis* | 100 | 99.28% | MW385265 |  |
| Camelia | R1-19S-E | AS-147 | OTU17 | *Pantoea* | *anthophila* | 100 | 99.84% | OL672164 |  |
| Camelia | R1-19S-O | AS150 | OTU17 | *Pantoea* | *ananatis* | 100 | 99.41% | MW385266 | Strong inhibition |
| Camelia | R1-19S-A1 | AS-182 | OTU17 | *Pantoea* | *anthophila* | 99 | 99.85% | OL672169 |  |
| Camelia | R1-19S-Q | AS-161 | OTU19 | *Pedobacter* | *suwonensis* | 100 | 99.06% | OL670751 | Biofilm |
| Camelia | R1-19S-S | AS-163 | OTU19 | *Pedobacter* | *suwonensis* | 100 | 98.99% | OL670752 |  |
| Camelia | R1-19S-X | AS-178 | OTU2 | *Herbaspirillum* | *aquaticum* | 100 | 99.86% | OL670760 | yellowing |
| Camelia | R1-19S-J | AS156 | OTU22 | *Rhizobium* | *nepotum* | 100 | 99.63% | MW369869 | No effect |
| Camelia | R1-19S-R | AS162 | OTU22 | *Rhizobium* | *nepotum* | 99 | 99.48% | MW369875 |  |
| Camelia | R1-19S-D | AS154 | OTU3 | *Acidovorax* | *avenae* | 100 | 99.26% | MW369868 | Color change |
| Camelia | R1-19S-G | AS-148 | OTU5 | *Acinetobacter* | *baylyi* | 99 | 99.56% | OL670747 | Color change |
| Camelia | R1-19S-M | AS151 | OTU5 | *Acinetobacter* | *baylyi* | 99 | 99.14% | MW369865 |  |
| Camelia | R1-19S-W | AS-152 | OTU5 | *Acinetobacter* | *baylyi* | 100 | 99.64% | OL670748 |  |
| Camelia | R1-19S-F | AS-155 | OTU50 | *Sphingomonas* | *paucimobilis* | 100 | 99.04% | OL670749 | Color change |
| Camelia | R1-19S-T | AS-164 | OTU50 | *Sphingomonas* | *paucimobilis* | 100 | 99.11% | OL670753 |  |
| Camelia | R1-19S-Y | AS-192 | OTU50 | *Sphingomonas* | *paucimobilis* | 99 | 99.04% | OL670769 |  |
| Camelia | R1-19S-U | AS-191 | OTU57 | *Pseudomonas* | *oryzihabitans* | 99 | 99.27% | OL670768 |  |
| Camelia | R1-19S-P | AS-160 | OTU6 | *Acinetobacter* | *guillouiae* | 99 | 100.00% | OL670750 | No effect |
| Camelia | R1-19S-A4 | AS-185 | OTU6 | *Acinetobacter* | *guillouiae* | 100 | 100.00% | OL670763 |  |
| Camelia | R1-19S-A5 | AS-186 | OTU8 | *Acinetobacter* | *calcoaceticus* | 99 | 99.93% | OL670764 | Color change |
| Camelia | R1-19S-H | AS149 | OTU9 | *Acinetobacter* | *lactucae* | 99 | 99.15% | MW369864 | Color change |
| Canilla | R1-21S-D | AS513 | OTU1 | *Acidovorax* | *wautersii* | 99 | 99.08% | MW370116 | Color change |
| Canilla | R1-21S-A | AS495 | OTU16 | *Pantoea* | *agglomerans* | 99 | 99.17% | MW385283 | No effect |
| Canilla | R1-21S-N | AS525 | OTU16 | *Pantoea* | *agglomerans* | 100 | 99.44% | MW385286 |  |
| Canilla | R1-21S-J | AS-510 | OTU23 | *Comamonas* | *sediminis* | 100 | 96.96% | OL670789 | Color change |
| Canilla | R1-21S-K | AS504 | OTU24 | *Curtobacterium* | *oceanosedimentum* | 99 | 99.07% | MW370108 | Color change |
| Canilla | R1-21S-F | AS498 | OTU27 | *Rouxiella* | *chamberiensis* | 99 | 99.93% | MW383971 | Strong inhibition |
| Canilla | R1-21S-B | AS496 | OTU46 | *Erwinia* | *endophytica* | 99 | 99.58% | MW370102 | No effect |
| Canilla | R1-21S-C | AS497 | OTU46 | *Erwinia* | *endophytica* | 99 | 99.36% | MW370103 |  |
| Canilla | R1-21S-L | AS523 | OTU46 | *Erwinia* | *endophytica* | 99 | 99.65% | MW370124 | No effect |
| Canilla | R1-21S-M | AS-524 | OTU46 | *Erwinia* | *endophytica* | 100 | 100.00% | OL672193 | No effect |
| Canilla | R1-21S-H | AS-511 | OTU50 | *Sphingomonas* | *paucimobilis* | 99 | 99.46% | OL672186 |  |
| Canilla | R1-21S-E | AS-512 | OTU50 | *Sphingomonas* | *paucimobilis* | 99 | 99.68% | OL672187 | No effect |
| Canilla | R1-21S-I | AS-499 | OTU51 | *Pseudomonas* | *lurida* | 100 | 100.00% | OL670786 | No effect |
| Cateto Nortista | R1-22S-B | AS-515 | OTU26 | *Clavibacter* | *michiganensis* | 100 | 99.93% | OL670790 | Biofilm |
| Cateto Nortista | R1-22S-A | AS-521 | OTU26 | *Clavibacter* | *michiganensis* | 100 | 100.00% | OL672191 |  |
| Cateto Nortista | R1-22S-E | AS-522 | OTU26 | *Clavibacter* | *michiganensis* | 100 | 100.00% | OL672192 |  |
| Cateto Nortista | R1-22S-F | AS-538 | OTU29 | *Frigoribacterium* | *faeni* | 99 | 99.55% | OL670795 | No effect |
| Cateto Nortista | R1-22S-H | AS534 | OTU30 | *Methylorubrum* | *extorguens* | 100 | 99.55% | MW370130 |  |
| Chapalote | R1-4S-A | AS-329 | OTU15 | *Pantoea* | *agglomerans* | 100 | 100.00% | OL672179 | No effect |
| Chapalote | R1-4S-B | AS-330 | OTU15 | *Pantoea* | *agglomerans* | 100 | 100.00% | OL672180 |  |
| Cristalino de Chihuahua | R1-7S-G | AS-59 | OTU10 | *Microbacterium* | *testaceum* | 99 | 99.64% | OL670798 |  |
| Cristalino de Chihuahua | R1-7S-N | AS76 | OTU10 | *Microbacterium* | *testaceum* | 100 | 99.14% | MW369810 | No effect |
| Cristalino de Chihuahua | R1-7S-J | AS-61 | OTU15 | *Pantoea* | *agglomerans* | 100 | 99.55% | OL672196 | light clearing |
| Cristalino de Chihuahua | L1-7S-F | AS-70 | OTU15 | *Pantoea* | *agglomerans* | 100 | 99.51% | OL672197 |  |
| Cristalino de Chihuahua | R1-7S-O | AS-77 | OTU15 | *Pantoea* | *agglomerans* | 100 | 99.64% | OL672198 | No effect |
| Cristalino de Chihuahua | L1-7S-A | AS-51 | OTU3 | *Acidovorax* | *avenae* | 100 | 99.64% | OL672185 |  |
| Cristalino de Chihuahua | R1-7S-I | AS-60 | OTU3 | *Acidovorax* | *avenae* | 100 | 99.41% | OL670799 | light clearing |
| Cristalino de Chihuahua | L1-7S-H | AS68 | OTU3 | *Acidovorax* | *avenae* | 99 | 99.42% | MW369802 | No effect |
| Cristalino de Chihuahua | L1-7S-E | AS-65 | OTU31 | *Paenibacillus* | *glucanolyticus* | 100 | 99.93% | OL670801 | No effect |
| Cristalino de Chihuahua | L1-7S-B | AS-52 | OTU43 | *Atlantibacter* | *hermannii* | 99 | 99.01% | OL670791 | light clearing |
| Cristalino de Chihuahua | L1-7S-D | AS54 | OTU43 | *Atlantibacter* | *hermannii* | 100 | 99.15% | MW369793 |  |
| Cristalino de Chihuahua | R1-7S-C | AS-57 | OTU43 | *Atlantibacter* | *hermannii* | 100 | 99.01% | OL670797 | Biofilm |
| Cristalino de Chihuahua | R1-7S-F | AS58 | OTU43 | *Atlantibacter* | *hermannii* | 100 | 98.66% | MW369795 |  |
| Cristalino de Chihuahua | R1-7S-P | AS-78 | OTU43 | *Atlantibacter* | *hermannii* | 99 | 99.25% | OL672199 | Biofilm |
| Cristalino de Chihuahua | R1-7S-K | AS62 | OTU49 | *Sphingomonas* | *yabuuchiae* | 100 | 99.78% | MW369799 | No effect |
| Cristalino de Chihuahua | L1-7S-C | AS-53 | OTU55 | *Pseudomonas* | *psychrotolerans* | 99 | 99.42% | OL670793 | Color change |
| Cristalino de Chihuahua | R1-7S-L | AS-63 | OTU55 | *Pseudomonas* | *psychrotolerans* | 100 | 99.42% | OL670800 |  |
| Cristalino de Chihuahua | L1-7S-G | AS-69 | OTU55 | *Pseudomonas* | *psychrotolerans* | 100 | 99.42% | OL670802 |  |
| Cristalino de Chihuahua | R1-7S-B | AS-71 | OTU55 | *Pseudomonas* | *psychrotolerans* | 99 | 99.42% | OL670803 |  |
| Cristalino de Chihuahua | R1-7S-D | AS-72 | OTU55 | *Pseudomonas* | *psychrotolerans* | 100 | 99.28% | OL670804 | No effect |
| Cristalino de Chihuahua | R1-7S-E | AS-73 | OTU55 | *Pseudomonas* | *psychrotolerans* | 99 | 99.42% | OL670805 |  |
| Cristalino de Chihuahua | R1-7S-H | AS74 | OTU55 | *Pseudomonas* | *psychrotolerans* | 99 | 99.21% | MW369808 |  |
| Cristalino de Chihuahua | R1-7S-M | AS75 | OTU55 | *Pseudomonas* | *Psychrotolerans* | 98 | 99.06% | MW369809 |  |
| Dente Branco | R1-18S-A | AS167 | OTU10 | *Microbacterium* | *testaceum* | 100 | 99.43% | MW369880 | Biofilm |
| Dente Branco | R1-18S-F | AS-180 | OTU11 | *Microbacterium* | *kyungheense* | 99 | 99.19% | OL670761 | No effect |
| Dente Branco | R1-18S-M | AS-181 | OTU11 | *Microbacterium* | *kyungheense* | 100 | 99.59% | OL672168 |  |
| Dente Branco | R1-18S-S | AS-188 | OTU11 | *Microbacterium* | *kyungheense* | 100 | 99.53% | OL672170 |  |
| Dente Branco | R1-18S-I | AS-171 | OTU12 | *Microbacterium* | *testaceum* | 100 | 99.20% | OL670755 |  |
| Dente Branco | R1-18S-C | AS-168 | OTU32 | *Chryseobacterium* | *lactis* | 100 | 99.50% | OL672166 | Biofilm |
| Dente Branco | R1-18S-L | AS-173 | OTU32 | *Chryseobacterium* | *lactis* | 100 | 99.43% | OL672167 | No effect |
| Dente Branco | R1-18S-P | AS174 | OTU4 | *Acidovorax* | *soli* | 99 | 98% | MW383962 | light clearing |
| Dente Branco | R1-18S-J | AS-172 | OTU49 | *Sphingomonas* | *yabuuchiae* | 99 | 99.78% | OL670756 | No effect |
| Dente Branco | R1-18S-D | AS-143 | OTU5 | *Acinetobacter* | *baylyi* | 99 | 99.14% | OL670745 | Color change |
| Dente Branco | R1-18S-G | AS169 | OTU52 | *Pseudomonas* | *poae* | 99 | 99.86% | MW369882 | Color change |
| Dente Branco | R1-18S-H | AS-170 | OTU55 | *Pseudomonas* | *psychrotolerans* | 99 | 99.42% | OL670754 | Color change |
| Dente Branco | R1-18S-Q | AS-175 | OTU55 | *Pseudomonas* | *psychrotolerans* | 99 | 99.35% | OL670757 |  |
| Dente Branco | R1-18S-R | AS-176 | OTU55 | *Pseudomonas* | *psychrotolerans* | 99 | 99.42% | OL670758 |  |
| Dente Branco | R1-18S-N | AS-177 | OTU55 | *Pseudomonas* | *psychrotolerans* | 99 | 99.28% | OL670759 |  |
| Dente Branco | R1-18S-E | AS-189 | OTU55 | *Pseudomonas* | *psychrotolerans* | 99 | 99.42% | OL670766 |  |
| Dente Branco | R1-18S-K | AS-190 | OTU55 | *Pseudomonas* | *psychrotolerans* | 99 | 99.42% | OL670767 |  |
| Dente Branco | R1-18S-O | AS-144 | OTU6 | *Acinetobacter* | *guillouiae* | 99 | 100.00% | OL670746 | no effect |
| Jala | R1-9S-A | AS-319 | OTU16 | *Pantoea* | *agglomerans* | 100 | 99.88% | OL672176 | No effect |
| Kulli | R1-24S-F | AS-508 | OTU1 | *Acidovorax* | *wautersii* | 99 | 99.85% | OL670787 | light clearing |
| Kulli | R1-24S-P | AS532 | OTU1 | *Acidovorax* | *wautersii* | 99 | 99.79% | MW370129 |  |
| Kulli | R1-24S-D | AS507 | OTU10 | *Microbacterium* | *testaceum* | 100 | 99.42% | MW370111 | No effect |
| Kulli | R1-24S-B | AS516 | OTU10 | *Microbacterium* | *testaceum* | 99 | 99.42% | MW370118 |  |
| Kulli | R1-24S-O | AS531 | OTU10 | *Microbacterium* | *testaceum* | 99 | 99.21% | MW370128 |  |
| Kulli | R1-24S-I | AS-503 | OTU15 | *Pantoea* | *agglomerans* | 100 | 99.72% | OL672184 | No effect |
| Kulli | R1-24S-N | AS-527 | OTU15 | *Pantoea* | *agglomerans* | 100 | 99.27% | OL672194 |  |
| Kulli | R1-24S-A | AS-500 | OTU17 | *Pantoea* | *ananatis* | 100 | 99.26% | OL672183 | color change |
| Kulli | R1-24S-G | AS501 | OTU18 | *Pantoea* | *dispersa* | 99 | 99.22% | MW370105 | Minor inhibition |
| Kulli | R1-24S-H | AS502 | OTU18 | *Pantoea* | *dispersa* | 99 | 99.08% | MW370106 | Strong inhibition |
| Kulli | R1-24S-K | AS-530 | OTU33 | *Chryseobacterium* | *hagamense* | 99 | 98.85% | OL670794 | No effect |
| Kulli | R1-24S-L | AS-565 | OTU36 | *Methylobacterium* | *bullatum* | 100 | 99.70% | OL670796 |  |
| Kulli | R1-24S-J | AS526 | OTU48 | *Sphingomonas* | *parapaucimobilis* | 100 | 99.16% | MW385287 | yellow biofilm |
| Kulli | R1-24S-C | AS-529 | OTU50 | *Sphingomonas* | *paucimobilis* | 100 | 99.04% | OL670792 | No effect |
| Nal-Tel | R1-10S-O | AS-315 | OTU1 | *Acidovorax* | *wautersii* | 100 | 100.00% | OL670774 | Color change |
| Nal-Tel | R1-10S-J | AS-331 | OTU11 | *Microbacterium* | *kyungheense* | 99 | 99.33% | OL670780 | No effect |
| Nal-Tel | R1-10S-I | AS-332 | OTU11 | *Microbacterium* | *testaceum* | 100 | 98.71% | OL670781 |  |
| Nal-Tel | R1-10S-S | AS-395 | OTU11 | *Microbacterium* | *kyungheense* | 99 | 99.41% | OL670782 |  |
| Nal-Tel | R1-10S-P | AS-316 | OTU13 | *Microbacterium* | *testaceum* | 100 | 99.34% | OL670775 | No effect |
| Nal-Tel | R1-10S-E | AS308 | OTU14 | *Microbacterium* | *neimengense* | 99 | 99.50% | MW370166 | No effect |
| Nal-Tel | R1-10S-B | AS-305 | OTU15 | *Pantoea* | *agglomerans* | 100 | 99.55% | OL672173 |  |
| Nal-Tel | R1-10S-H | AS-310 | OTU15 | *Pantoea* | *agglomerans* | 99 | 99.14% | OL672174 |  |
| Nal-Tel | R1-10S-R | AS-318 | OTU15 | *Pantoea* | *agglomerans* | 100 | 99.05% | OL672175 | Color change |
| Nal-Tel | R1-10S-A | AS-304 | OTU20 | *Pedobacter* | *suwonensis* | 100 | 99.26% | OL672172 | No effect |
| Nal-Tel | R1-10S-D | AS307 | OTU34 | *Chryseobacterium* | *camelliae* | 99 | 99.64% | MW370165 | No effect |
| Nal-Tel | R1-10S-K | AS311 | OTU35 | *Agrobacterium* | *larrymoorei* | 99 | 99.19% | MW370169 | No effect |
| Nal-Tel | R1-10S-N | AS-314 | OTU5 | *Acinetobacter* | *baylyi* | 99 | 99.79% | OL670773 |  |
| Nal-Tel | R1-10S-M | AS313 | OTU53 | *Pseudomonas* | *syringae* | 99 | 99.50% | MW370171 | Color change |
| Nal-Tel | R1-10S-Q | AS84 | OTU53 | *Pseudomonas* | *syringae* | 99 | 99.43% | MW370175 | Minor inhibition |
| Nal-Tel | R1-10S-C | AS-306 | OTU7 | *Acinetobacter* | *soli* | 98 | 99.78% | OL670770 | color change |
| Nal-Tel | R1-10S-G | AS-309 | OTU7 | *Acinetobacter* | *soli* | 100 | 100.00% | OL670771 |  |
| Nal-Tel | R1-10S-L | AS-312 | OTU7 | *Acinetobacter* | *soli* | 100 | 99.93% | OL670772 |  |
| Oloton | R1-16S-A1 | AS-112 | OTU28 | *Rouxiella* | *chamberiensis* | 100 | 99.34% | OL670733 | Strong inhibition |
| Oloton | R1-16S-D | AS-81 | OTU28 | *Rouxiella* | *chamberiensis* | 100 | 99.08% | OL670806 |  |
| Oloton | R1-16S-F | AS83 | OTU28 | *Rouxiella* | *chamberiensis* | 99 | 99.07% | MW644742 | Strong inhibition |
| Oloton | R1-16S-P | AS-92 | OTU28 | *Rouxiella* | *chamberiensis* | 100 | 99.16% | OL670810 |  |
| Oloton | L1-16S-C | AS100 | OTU40 | *Ewingella* | *americana* | 99 | 99.36% | MW369828 | Strong inhibition |
| Oloton | R1-16S-Y | AS-111 | OTU40 | *Ewingella* | *americana* | 100 | 99.57% | OL670732 |  |
| Oloton | R1-16S-G | AS84 | OTU40 | *Ewingella* | *americana* | 100 | 99.78% | MW644739 | Strong inhibition |
| Oloton | R1-16S-M | AS-90 | OTU40 | *Ewingella* | *americana* | 100 | 99.36% | OL670809 |  |
| Oloton | R1-16S-N | AS91 | OTU40 | *Ewingella* | *americana* | 100 | 99.40% | MW644749 | Minor inhibition |
| Oloton | R1-16S-R | AS-94 | OTU40 | *Ewingella* | *americana* | 100 | 100.00% | OL672201 |  |
| Oloton | R1-16S-W | AS-96 | OTU40 | *Ewingella* | *americana* | 100 | 99.50% | OL670811 |  |
| Oloton | R1-16S-I | AS86 | OTU41 | *Rahnella* | *victoriana* | 98 | 99.63% | MW644746 | Minor inhibition+Clearing |
| Oloton | R1-16S-S | AS95 | OTU42 | *Rahnella* | *aquatilis* | 99 | 99.29% | MW369824 | Strong inhibition |
| Oloton | R1-16S-T | AS-110 | OTU54 | *Pseudomonas* | *koreensis* | 100 | 100.00% | OL670731 |  |
| Oloton | R1-16S-A2 | AS-123 | OTU54 | *Pseudomonas* | *koreensis* | 100 | 99.27% | OL670736 |  |
| Oloton | R1-16S-B | AS80 | OTU54 | *Pseudomonas* | *koreensis* | 100 | 99.41% | MW644740 | Strong inhibition |
| Oloton | R1-16S-H | AS-85 | OTU54 | *Pseudomonas* | *koreensis* | 100 | 99.25% | OL670807 |  |
| Oloton | R1-16S-J | AS-87 | OTU54 | *Pseudomonas* | *koreensis* | 99 | 99.93% | OL670808 |  |
| Oloton | R1-16S-L | AS89 | OTU54 | *Pseudomonas* | *koreensis* | 100 | 99.41% | MW644741 | Strong inhibition |
| Oloton | R1-16S-Q | AS-93 | OTU56 | *Pseudomonas* | *azotoformans* | 99 | 99.86% | OL672200 | color change |
| Oloton | L1-16S-B | AS-99 | OTU56 | *Pseudomonas* | *chlororaphis* | 99 | 100.00% | OL672202 |  |
| Oloton | L1-16S-A | AS-98 | OTU57 | *Pseudomonas* | *helleri* | 99 | 99.86% | OL670812 | color change |
| Oloton | R1-16S-O | AS-120 | OTU60 | *Leuconostoc* | *mesenteroides* | 100 | 99.72% | OL670735 | No effect |
| Oloton | R1-16S-U | AS-218 | OTU61 | *Variovorax* | *boronicumulans* | 100 | 98.14% | OL672171 |  |
| Palomero Toluqueno | R1-13S-D | AS-322 | OTU1 | *Acidovorax* | *wautersii* | 100 | 99.21% | OL672177 | color change |
| Palomero Toluqueno | R1-13S-H | AS-324 | OTU1 | *Acidovorax* | *wautersii* | 100 | 99.93% | OL670777 |  |
| Palomero Toluqueno | R1-13S-L | AS328 | OTU1 | *Acidovorax* | *wautersii* | 99 | 100.00% | MW383966 | light clearing |
| Palomero Toluqueno | R1-13S-E | AS-333 | OTU10 | *Microbacterium* | *testaceum* | 100 | 99.51% | OL672181 | No effect |
| Palomero Toluqueno | R1-13S-G | AS-394 | OTU11 | *Microbacterium* | *kyungheense* | 100 | 100.00% | OL672182 | No effect |
| Palomero Toluqueno | R1-13S-B | AS-567 | OTU37 | *Methylobacterium* | *gossipiicola* | 99 | 100.00% | OL672195 |  |
| Palomero Toluqueno | R1-13S-J | AS-326 | OTU40 | *Aureimonas* | *ureilytica* | 99 | 99.48% | OL670779 | No effect |
| Palomero Toluqueno | R1-13S-K | AS-327 | OTU40 | *Aureimonas* | *ureilytica* | 100 | 98.80% | OL672178 |  |
| Palomero Toluqueno | R1-13S-F | AS323 | OTU49 | *Sphingomonas* | *yabuuchiae* | 99 | 99.12% | MW370179 |  |
| Palomero Toluqueno | R1-13S-I | AS-325 | OTU49 | *Sphingomonas* | *yabuuchiae* | 99 | 99.71% | OL670778 | No effect |
| Palomero Toluqueno | R1-13S-A | AS-320 | OTU8 | *Acinetobacter* | *calcoaceticus* | 100 | 100.00% | OL670776 | color change |
| Vandeno | R1-15S-B | AS-103 | OTU29 | *Frigoribacterium* | *faeni* | 100 | 99.86% | OL672154 | No effect |
| Vandeno | R1-15S-G | AS-106 | OTU29 | *Frigoribacterium* | *faeni* | 100 | 99.27% | OL670729 |  |
| Vandeno | R1-15S-M | AS124 | OTU39 | *Plantibacter* | *flavus* | 99 | 99.14% | MW369844 | No effect |
| Vandeno | R1-15S-N | AS125 | OTU39 | *Plantibacter* | *flavus* | 100 | 99.64% | MW383960 |  |
| Vandeno | R1-15S-C | AS115 | OTU44 | *Aeromicrobium* | *erythreum* | 99 | 99.28% | MW369838 | No effect |
| Vandeno | R1-15S-E | AS116 | OTU45 | *Aeromicrobium* | *ginsengisoli* | 99 | 99.39% | MW384860 | No effect |
| Vandeno | R1-15S-J | AS117 | OTU45 | *Aeromicrobium* | *fastidiosum* | 99 | 99.06% | MW369839 |  |
| Vandeno | L1-15S-A | AS-137 | OTU45 | *Aeromicrobium* | *fastidiosum* | 99 | 99.13% | OL670742 | No effect |
| Vandeno | L1-15S-B | AS-138 | OTU45 | *Aeromicrobium* | *fastidiosum* | 99 | 99.20% | OL670743 |  |
| Vandeno | L1-15S-C | AS139 | OTU45 | *Aeromicrobium* | *fastidiosum* | 99 | 99.13% | MW369857 |  |
| Vandeno | R1-15S-K | AS-140 | OTU45 | *Aeromicrobium* | *fastidiosum* | 100 | 99.06% | OL670744 |  |
| Vandeno | R1-15S-L | AS141 | OTU45 | *Aeromicrobium* | *fastidiosum* | 99 | 99.27% | MW369859 |  |
| Vandeno | R1-15S-O | AS142 | OTU45 | *Aeromicrobium* | *fastidiosum* | 99 | 99.13% | MW369860 |  |
| Vandeno | R1-15S-H | AS107 | OTU52 | *Pseudomonas* | *poae* | 99 | 99.29% | MW369833 | yellowing |
| Vandeno | R1-15S-D | AS-104 | OTU53 | *Pseudomonas* | *syringae* | 100 | 99.72% | OL672155 |  |
| Vandeno | R1-15S-I | AS-108 | OTU53 | *Pseudomonas* | *syringae* | 99 | 99.50% | OL670730 |  |
| Vandeno | R1-15S-A | AS-102 | OTU58 | *Pseudomonas* | *cannabina* | 99 | 99.57% | OL670728 | No effect |
| Vandeno | R1-15S-F | AS-105 | OTU59 | *Pseudomonas* | *cerasi* | 100 | 100.00% | OL672156 | No effect |
| Zapalote Chico | R1-14S-C | AS101 | OTU17 | *Pantoea* | *ananatis* | 100 | 99.42% | MW385263 | No effect |
| Zapalote Chico | R1-14S-F | AS-128 | OTU25 | *Curtobacterium* | *herbarum* | 100 | 99.74% | OL672160 | No effect |
| Zapalote Chico | R1-14S-G | AS-129 | OTU25 | *Curtobacterium* | *flaccumfaciens* | 100 | 99.85% | OL672161 |  |
| Zapalote Chico | R1-14S-H | AS-130 | OTU25 | *Curtobacterium* | *flaccumfaciens* | 100 | 99.86% | OL672162 |  |
| Zapalote Chico | R1-14S-I | AS-131 | OTU25 | *Curtobacterium* | *flaccumfaciens* | 99 | 98.91% | OL670737 |  |
| Zapalote Chico | R1-14S-A | AS-126 | OTU29 | *Frigoribacterium* | *faeni* | 100 | 99.82% | OL672158 | No effect |
| Zapalote Chico | R1-14S-D | AS-127 | OTU29 | *Frigoribacterium* | *faeni* | 100 | 99.84% | OL672159 |  |
| Zapalote Chico | R1-14S-B | AS-118 | OTU45 | *Aeromicrobium* | *fastidiosum* | 99 | 90.01% | OL672157 | No effect |
| Zapalote Chico | R1-14S-E | AS-119 | OTU45 | *Aeromicrobium* | *fastidiosum* | 100 | 99.20% | OL670734 |  |
| Zapalote Chico | R1-14S-J | AS-132 | OTU45 | *Aeromicrobium* | *fastidiosum* | 99 | 99.13% | OL670738 | No effect |
| Zapalote Chico | R1-14S-K | AS-133 | OTU45 | *Aeromicrobium* | *fastidiosum* | 100 | 99.20% | OL670739 |  |
| Zapalote Chico | R1-14S-L | AS-134 | OTU45 | *Aeromicrobium* | *fastidiosum* | 99 | 98.99% | OL670740 |  |
| Zapalote Chico | R1-14S-M | AS-135 | OTU45 | *Aeromicrobium* | *fastidiosum* | 100 | 99.22% | OL670741 |  |
| Wild Mexicana | R1-2S-D | AS-514 | OTU17 | *Pantoea* | *ananatis* | 100 | 99.49% | OL672188 | light clearing |
| Wild Mexicana | R1-2S-K | AS528 | OTU2 | *Herbaspirillum* | *huttiense* | 100 | 99.86% | MW385288 | No effect |
| Wild Mexicana | R1-2S-B | AS-490 | OTU21 | *Pedobacter* | *terrae* | 100 | 99.04% | OL670783 | No effect |
| Wild Mexicana | R1-2S-C | AS-519 | OTU38 | *Duganella* | *zoogloeoides* | 100 | 99.16% | OL672189 | No effect |
| Wild Mexicana | R1-2S-H | AS-520 | OTU38 | *Duganella* | *zoogloeoides* | 99 | 99.31% | OL672190 |  |
| Wild Mexicana | R1-2S-I | AS-509 | OTU47 | *Erwinia* | *billingiae* | 99 | 98.51% | OL670788 | light clearing |
| Wild Mexicana | R1-2S-G | AS-493 | OTU48 | *Sphingomonas* | *parapaucimobilis* | 99 | 99.70% | OL670785 | No effect |
| Wild Mexicana | R1-2S-F | AS-492 | OTU49 | *Sphingomonas* | *yabuuchiae* | 100 | 99.77% | OL670784 | No effect |
| Wild Mexicana | R1-2S-E | AS491 | OTU51 | *Pseudomonas* | *lurida* | 99 | 99.86% | MW370098 | No effect |
| Wild Mexicana | R1-2S-J | AS494 | OTU51 | *Pseudomonas* | *lurida* | 99 | 99.64% | MW370101 | No effect |
| Wild Mexicana | R1-2S-A | AS489 | OTU52 | *Pseudomonas* | *poae* | 99 | 99.64% | MW370096 | yellowing |

Supplemental Table 3. Whole genome mining of unpollinated silk-associated anti-*Fusarium* bacterial strains to identify genes previously shown to control *Fusarium graminearum* and/or other fungal pathogens.

| **Bacterial strains** | **OTU** | **Taxonomy** | | **Presence of Anti-*Fusarium*/Anti-Fungal Genes** | | | | | | | | | | | | |
| --- | --- | --- | --- | --- | --- | --- | --- | --- | --- | --- | --- | --- | --- | --- | --- | --- |
|  |  | **16S** | **WGS** | **Phenazine biosynthesis (*phzF*)** | **Redox balance (Nitronate monooxygenase)** | **Acetoin biosynthesis** | **2,3-Butanediol biosynthesis (Butanediol dehydrogenase)** | **Chitosanase/chitinase** | **Surfactin synthetase (srfAA)** | **Colicin V biosynthesis** | **Iturin biosynthesis (ituAC)** | **Fusarium acid resistance (fusE)** | **Diacetylphloroglucinol biosynthesis (phlACBD)** | **Fengycin biosynthesis (fenC, fenD)** | **Bacillomycin D synthetase (bmyB)** | **Total** |
| **AS112** | OTU28 | *Rouxiella chamberiensis* | *Rouxiella badensis* |  |  |  |  |  |  |  |  |  |  |  |  | **3** |
| **AS150** | OTU17 | *Pantoea ananatis* | *Pantoea ananatis* |  |  |  |  |  |  |  |  |  |  |  |  | **3** |
| **AS89** | OTU54 | *Pseudomonas koreensis* | *Unclassified Pseudomonas* |  |  |  |  |  |  |  |  |  |  |  |  | **2** |
| **AS95** | OTU42 | *Rahnella aquatilis* | *Rahnella aquatilis* |  |  |  |  |  |  |  |  |  |  |  |  | **3** |
| **AS100** | OTU40 | *Ewingella americana* | *Ewingella americana* |  |  |  |  |  |  |  |  |  |  |  |  | **3** |
| **AS501** | OTU18 | *Pantoea dispersa* | *Pantoea dispersa* |  |  |  |  |  |  |  |  |  |  |  |  | **3** |

**Supplementary Table 4: SAS statistical output results of individual kernel disease severity scoring**

**Supplementary Table 4A.1. Greenhouse Trial 1 Kernel disease severity scoring SAS statistical output**

| **Greenhouse Trial 1 Kernel disease severity at the individual kernel level (Treatment Least Squares Means): For Scale 4** | | | | | | | | |
| --- | --- | --- | --- | --- | --- | --- | --- | --- |
| **Trmt** | **Estimate** | **St. error** | **DF** | **t Value** | **Pr > \|t\|** | **Mean** | **SEM** | **TK Letters** |
| AS112 | -6.6248 | 0.4643 | 84 | -14.27 | <.0001 | 0.001325 | 0.000614 | c |
| AS150 | -3.0697 | 0.3283 | 84 | -9.35 | <.0001 | 0.04437 | 0.01392 | b |
| LB Control | 1.0898 | 0.3238 | 84 | 3.37 | 0.0012 | 0.7483 | 0.06098 | a |
| Proline PC | -6.9962 | 0.4977 | 84 | -14.06 | <.0001 | 0.000915 | 0.000455 | c |

**Supplementary Table 4A.2. Greenhouse Trial 1 Kernel disease severity multiple comparisons SAS output**

| **Greenhouse Trial 1 Kernel disease severity differences of trmt Least Square Means Adjustment for Multiple Comparisons: Tukey-Kramer: For Scale 4** | | | | | | | |
| --- | --- | --- | --- | --- | --- | --- | --- |
| **Trmt** | **_trmt** | **Estimate** | **St. error** | **DF** | **t Value** | **Pr > \|t\|** | **Adj P** |
| AS112 | AS150 | -3.5551 | 0.3394 | 84 | -10.48 | <.0001 | <.0001 |
| AS112 | LB Control | -7.7146 | 0.3369 | 84 | -22.90 | <.0001 | <.0001 |
| AS112 | Proline PC | 0.3714 | 0.5046 | 84 | 0.74 | 0.4638 | 0.8823 |
| AS150 | LB Control | -4.1595 | 0.07532 | 84 | -55.23 | <.0001 | <.0001 |
| AS150 | Proline PC | 3.9265 | 0.3835 | 84 | 10.24 | <.0001 | <.0001 |
| LB Control | Proline PC | 8.0860 | 0.3818 | 84 | 21.18 | <.0001 | <.0001 |

**Supplementary Table 4A.3. Greenhouse Trial 1 Kernel disease severity scoring SAS statistical output**

| **Greenhouse Trial 1 Kernel disease severity at the individual kernel level (Treatment Least Squares Means): For Scale 3** | | | | | | | | |
| --- | --- | --- | --- | --- | --- | --- | --- | --- |
| **Trmt** | **Estimate** | **St. error** | **DF** | **t Value** | **Pr > \|t\|** | **Mean** | **SEM** | **TK Letters** |
| AS112 | -5.4910 | 0.2849 | 84 | -19.27 | <.0001 | 0.004107 | 0.001165 | c |
| AS150 | -3.2202 | 0.2166 | 84 | -14.87 | <.0001 | 0.03841 | 0.008002 | a |
| LB Control | -3.0707 | 0.2162 | 84 | -14.20 | <.0001 | 0.04433 | 0.009158 | a |
| Proline PC | -4.5755 | 0.2402 | 84 | -19.05 | <.0001 | 0.01020 | 0.002424 | b |

**Supplementary Table 4A.4. Greenhouse Trial 1 Kernel disease severity multiple comparisons SAS output**

| **Greenhouse Trial 1 Kernel disease severity differences of trmt Least Square Means Adjustment for Multiple Comparisons: Tukey-Kramer: For Scale 3** | | | | | | | |
| --- | --- | --- | --- | --- | --- | --- | --- |
| **Trmt** | **_trmt** | **Estimate** | **St. error** | **DF** | **t Value** | **Pr > \|t\|** | **Adj P** |
| AS112 | AS150 | -2.2708 | 0.2078 | 84 | -10.93 | <.0001 | <.0001 |
| AS112 | LB Control | -2.4203 | 0.2071 | 84 | -11.69 | <.0001 | <.0001 |
| AS112 | Proline PC | -0.9155 | 0.2321 | 84 | -3.94 | 0.0002 | 0.0009 |
| AS150 | LB Control | -0.1495 | 0.09327 | 84 | -1.60 | 0.1128 | 0.3828 |
| AS150 | Proline PC | 1.3553 | 0.1402 | 84 | 9.66 | <.0001 | <.0001 |
| LB Control | Proline PC | 1.5047 | 0.1390 | 84 | 10.83 | <.0001 | <.0001 |

**Supplementary Table 4A.5. Greenhouse Trial 1 Kernel disease severity scoring SAS statistical output**

| **Greenhouse Trial 1 Kernel disease severity at the individual kernel level (Treatment Least Squares Means): For Scale 2** | | | | | | | | |
| --- | --- | --- | --- | --- | --- | --- | --- | --- |
| **Trmt** | **Estimate** | **St. error** | **DF** | **t Value** | **Pr > \|t\|** | **Mean** | **SEM** | **TK Letters** |
| AS112 | -3.6959 | 0.1184 | 84 | -31.21 | <.0001 | 0.02422 | 0.002800 | d |
| AS150 | -2.8273 | 0.1005 | 84 | -28.14 | <.0001 | 0.05586 | 0.005299 | b |
| LB Control | -2.0694 | 0.09332 | 84 | -22.18 | <.0001 | 0.1121 | 0.009289 | a |
| Proline PC | -3.2418 | 0.1069 | 84 | -30.32 | <.0001 | 0.03762 | 0.003872 | c |

**Supplementary Table 4A.6. Greenhouse Trial 1 Kernel disease severity multiple comparisons SAS output**

| **Greenhouse Trial 1 Kernel disease severity differences of trmt Least Square Means Adjustment for Multiple Comparisons: Tukey-Kramer: For Scale 2** | | | | | | | |
| --- | --- | --- | --- | --- | --- | --- | --- |
| **Trmt** | **_trmt** | **Estimate** | **St. error** | **DF** | **t Value** | **Pr > \|t\|** | **Adj P** |
| AS112 | AS150 | -0.8685 | 0.1031 | 84 | -8.42 | <.0001 | <.0001 |
| AS112 | LB Control | -1.6265 | 0.09639 | 84 | -16.87 | <.0001 | <.0001 |
| AS112 | Proline PC | -0.4540 | 0.1095 | 84 | -4.15 | <.0001 | 0.0005 |
| AS150 | LB Control | -0.7580 | 0.07319 | 84 | -10.36 | <.0001 | <.0001 |
| AS150 | Proline PC | 0.4145 | 0.08980 | 84 | 4.62 | <.0001 | <.0001 |
| LB Control | Proline PC | 1.1724 | 0.08165 | 84 | 14.36 | <.0001 | <.0001 |

**Supplementary Table 4A.7. Greenhouse Trial 1 Kernel disease severity scoring SAS statistical output**

| **Greenhouse Trial 1 Kernel disease severity at the individual kernel level (Treatment Least Squares Means): For Scale 1** | | | | | | | | |
| --- | --- | --- | --- | --- | --- | --- | --- | --- |
| **Trmt** | **Estimate** | **St. error** | **DF** | **t Value** | **Pr > \|t\|** | **Mean** | **SEM** | **TK Letters** |
| AS112 | 3.5460 | 0.2848 | 84 | 12.45 | <.0001 | 0.9720 | 0.007760 | a |
| AS150 | 1.8111 | 0.2768 | 84 | 6.54 | <.0001 | 0.8595 | 0.03343 | c |
| LB Control | -2.2286 | 0.2782 | 84 | -8.01 | <.0001 | 0.09721 | 0.02442 | d |
| Proline PC | 3.0756 | 0.2809 | 84 | 10.95 | <.0001 | 0.9559 | 0.01185 | b |

**Supplementary Table 4A.8. Greenhouse Trial 1 Kernel disease severity multiple comparisons SAS output**

| **Greenhouse Trial 1 Kernel disease severity differences of trmt Least Square Means Adjustment for Multiple Comparisons: Tukey-Kramer: For Scale 1** | | | | | | | |
| --- | --- | --- | --- | --- | --- | --- | --- |
| **Trmt** | **_trmt** | **Estimate** | **St. error** | **DF** | **t Value** | **Pr > \|t\|** | **Adj P** |
| AS112 | AS150 | 1.7348 | 0.08583 | 84 | 20.21 | <.0001 | <.0001 |
| AS112 | LB Control | 5.7746 | 0.09281 | 84 | 62.22 | <.0001 | <.0001 |
| AS112 | Proline PC | 0.4703 | 0.09771 | 84 | 4.81 | <.0001 | <.0001 |
| AS150 | LB Control | 4.0398 | 0.06390 | 84 | 63.22 | <.0001 | <.0001 |
| AS150 | Proline PC | -1.2645 | 0.07144 | 84 | -17.70 | <.0001 | <.0001 |
| LB Control | Proline PC | -5.3043 | 0.08003 | 84 | -66.28 | <.0001 | <.0001 |

**Supplementary Table 4B.1. Greenhouse Trial 2 Kernel disease severity scoring SAS statistical output**

| **Greenhouse Trial 2 Kernel disease severity at the individual kernel level (Treatment Least Squares Means): For Scale 4** | | | | | | | | |
| --- | --- | --- | --- | --- | --- | --- | --- | --- |
| **Trmt** | **Estimate** | **St. error** | **DF** | **t Value** | **Pr > \|t\|** | **Mean** | **SEM** | **TK Letters** |
| AS112 | -7.3000 | 0.5487 | 85 | -13.31 | <.0001 | 0.000675 | 0.000370 | c |
| AS150 | -2.8594 | 0.3695 | 85 | -7.74 | <.0001 | 0.05420 | 0.01894 | b |
| LBControl | 0.7618 | 0.3666 | 85 | 2.08 | 0.0407 | 0.6818 | 0.07954 | a |
| Proline | -6.5551 | 0.4665 | 85 | -14.05 | <.0001 | 0.001421 | 0.000662 | c |

**Supplementary Table 4B.2. Greenhouse Trial 2 Kernel disease severity multiple comparisons SAS output**

| **Greenhouse Trial 2 Kernel disease severity differences of trmt Least Square Means Adjustment for Multiple Comparisons: Tukey-Kramer: For Scale 4** | | | | | | | |
| --- | --- | --- | --- | --- | --- | --- | --- |
| **Trmt** | **_trmt** | **Estimate** | **St. error** | **DF** | **t Value** | **Pr > \|t\|** | **Adj P** |
| AS112 | AS150 | -4.4406 | 0.4120 | 85 | -10.78 | <.0001 | <.0001 |
| AS112 | LBControl | -8.0619 | 0.4112 | 85 | -19.61 | <.0001 | <.0001 |
| AS112 | Proline | -0.7449 | 0.5006 | 85 | -1.49 | 0.1404 | 0.4491 |
| AS150 | LBControl | -3.6212 | 0.06644 | 85 | -54.50 | <.0001 | <.0001 |
| AS150 | Proline | 3.6957 | 0.2940 | 85 | 12.57 | <.0001 | <.0001 |
| LBControl | Proline | 7.3169 | 0.2926 | 85 | 25.00 | <.0001 | <.0001 |

**Supplementary Table 4B.3. Greenhouse Trial 2 Kernel disease severity scoring SAS statistical output**

| **Greenhouse Trial 2 Kernel disease severity at the individual kernel level (Treatment Least Squares Means): For Scale 3** | | | | | | | | |
| --- | --- | --- | --- | --- | --- | --- | --- | --- |
| **Trmt** | **Estimate** | **St. error** | **DF** | **t Value** | **Pr > \|t\|** | **Mean** | **SEM** | **TK Letters** |
| AS112 | -6.1834 | 0.3390 | 85 | -18.24 | <.0001 | 0.002059 | 0.000697 | c |
| AS150 | -3.6963 | 0.2326 | 85 | -15.89 | <.0001 | 0.02422 | 0.005497 | b |
| LBControl | -1.9280 | 0.2224 | 85 | -8.67 | <.0001 | 0.1270 | 0.02465 | a |
| Proline | -5.4911 | 0.2858 | 85 | -19.21 | <.0001 | 0.004106 | 0.001169 | c |

**Supplementary Table 4B.4. Greenhouse Trial 2 Kernel disease severity multiple comparisons SAS output**

| **Greenhouse Trial 2 Kernel disease severity differences of trmt Least Square Means Adjustment for Multiple Comparisons: Tukey-Kramer: For Scale 3** | | | | | | | |
| --- | --- | --- | --- | --- | --- | --- | --- |
| **Trmt** | **_trmt** | **Estimate** | **St. error** | **DF** | **t Value** | **Pr > \|t\|** | **Adj P** |
| AS112 | AS150 | -2.4872 | 0.2701 | 85 | -9.21 | <.0001 | <.0001 |
| AS112 | LBControl | -4.2554 | 0.2617 | 85 | -16.26 | <.0001 | <.0001 |
| AS112 | Proline | -0.6923 | 0.3169 | 85 | -2.18 | 0.0317 | 0.1359 |
| AS150 | LBControl | -1.7682 | 0.08760 | 85 | -20.19 | <.0001 | <.0001 |
| AS150 | Proline | 1.7949 | 0.1991 | 85 | 9.02 | <.0001 | <.0001 |
| LBControl | Proline | 3.5631 | 0.1876 | 85 | 19.00 | <.0001 | <.0001 |

**Supplementary Table 4B.5. Greenhouse Trial 2 Kernel disease severity scoring SAS statistical output**

| **Greenhouse Trial 2 Kernel disease severity at the individual kernel level (Treatment Least Squares Means): For Scale 2** | | | | | | | | |
| --- | --- | --- | --- | --- | --- | --- | --- | --- |
| **Trmt** | **Estimate** | **St. error** | **DF** | **t Value** | **Pr > \|t\|** | **Mean** | **SEM** | **TK Letters** |
| AS112 | -3.4131 | 0.1198 | 85 | -28.48 | <.0001 | 0.03189 | 0.003699 | c |
| AS150 | -2.6112 | 0.1089 | 85 | -23.98 | <.0001 | 0.06842 | 0.006939 | b |
| LBControl | -2.0235 | 0.1052 | 85 | -19.23 | <.0001 | 0.1168 | 0.01085 | a |
| Proline | -3.6013 | 0.1244 | 85 | -28.95 | <.0001 | 0.02656 | 0.003217 | c |

**Supplementary Table 4B.6. Greenhouse Trial 2 disease severity multiple comparisons SAS output**

| **Greenhouse Trial 2 Kernel disease severity differences of trmt Least Square Means Adjustment for Multiple Comparisons: Tukey-Kramer: For Scale 2** | | | | | | | |
| --- | --- | --- | --- | --- | --- | --- | --- |
| **Trmt** | **_trmt** | **Estimate** | **St. error** | **DF** | **t Value** | **Pr > \|t\|** | **Adj P** |
| AS112 | AS150 | -0.8019 | 0.08655 | 85 | -9.27 | <.0001 | <.0001 |
| AS112 | LBControl | -1.3896 | 0.08209 | 85 | -16.93 | <.0001 | <.0001 |
| AS112 | Proline | 0.1883 | 0.1055 | 85 | 1.78 | 0.0779 | 0.2880 |
| AS150 | LBControl | -0.5877 | 0.06495 | 85 | -9.05 | <.0001 | <.0001 |
| AS150 | Proline | 0.9902 | 0.09281 | 85 | 10.67 | <.0001 | <.0001 |
| LBControl | Proline | 1.5778 | 0.08867 | 85 | 17.79 | <.0001 | <.0001 |

**Supplementary Table 4B.7. Greenhouse Trial 2 Kernel disease severity scoring SAS statistical output**

| **Greenhouse Trial 2 Kernel disease severity at the individual kernel level (Treatment Least Squares Means): For Scale 1** | | | | | | | | |
| --- | --- | --- | --- | --- | --- | --- | --- | --- |
| **Trmt** | **Estimate** | **St. error** | **DF** | **t Value** | **Pr > \|t\|** | **Mean** | **SEM** | **TK Letters** |
| AS112 | 3.3702 | 0.2125 | 85 | 15.86 | <.0001 | 0.9668 | 0.006830 | a |
| AS150 | 1.6485 | 0.2043 | 85 | 8.07 | <.0001 | 0.8387 | 0.02764 | b |
| LBControl | -2.4011 | 0.2069 | 85 | -11.61 | <.0001 | 0.08309 | 0.01576 | c |
| Proline | 3.4236 | 0.2134 | 85 | 16.04 | <.0001 | 0.9684 | 0.006524 | a |

**Supplementary Table 4B.8. Greenhouse Trial 2 disease severity multiple comparisons SAS output**

| **Greenhouse Trial 2 Kernel disease severity differences of trmt Least Square Means Adjustment for Multiple Comparisons: Tukey-Kramer: For Scale 1** | | | | | | | |
| --- | --- | --- | --- | --- | --- | --- | --- |
| **Trmt** | **_trmt** | **Estimate** | **St. error** | **DF** | **t Value** | **Pr > \|t\|** | **Adj P** |
| AS112 | AS150 | 1.7217 | 0.07637 | 85 | 22.54 | <.0001 | <.0001 |
| AS112 | LBControl | 5.7713 | 0.08464 | 85 | 68.19 | <.0001 | <.0001 |
| AS112 | Proline | -0.05344 | 0.09774 | 85 | -0.55 | 0.5860 | 0.9472 |
| AS150 | LBControl | 4.0496 | 0.06036 | 85 | 67.09 | <.0001 | <.0001 |
| AS150 | Proline | -1.7751 | 0.07875 | 85 | -22.54 | <.0001 | <.0001 |
| LBControl | Proline | -5.8247 | 0.08673 | 85 | -67.16 | <.0001 | <.0001 |

Supplemental Table 5. Type III secretion system operons with their functional name encoded in the genomes of unpollinated silk-associated anti-*Fg* bacteria that were tested in the replicated greenhouse trials.

| **Type III secretion system operons** | **Silk-associated bacteria** | | | |
| --- | --- | --- | --- | --- |
|  | **AS112 (*Rouxiella badensis*)** | | | **AS150 (*Pantoea ananatis*)** |
| Secretin |  | EscC/YscC/HrcC |  |  |
| Outer MS ring protein | SctD |  |  |  |
| Inner MS ring protein | SctJ |  |  |  |
| Minor export apparatus protein | SctR | EscR/YscR/HrcR |  | FliP |
| Minor export apparatus protein | SctS |  |  | FliQ |
| Minor export apparatus protein | SctT |  |  | FliR |
| Export apparatus switch protein |  | EscU/YscU/HrcU |  | FlhB |
| Major export apparatus protein |  | EscV/YscV/HrcV |  | FlhA |
| C ring protein | SctQ |  |  |  |
| Stator |  |  |  | FliH |
| ATPase | SctN | EscN/YscN/HrcN |  |  |
| Stalk |  |  |  |  |
| Needle Filament protein | SctF |  |  |  |
| Inner rod protein | SctI |  |  |  |
