# Supplementary figures and images for: Bacteria existing in pre-pollinated styles (silks) can defend the exposed male gamete fertilization channel of maize against an environmental Fusarium pathogen
Source: Front Plant Sci. 2023 Dec 4;14:1292109. doi: 10.3389/fpls.2023.1292109 (PMC10726056; doi:10.3389/fpls.2023.1292109)

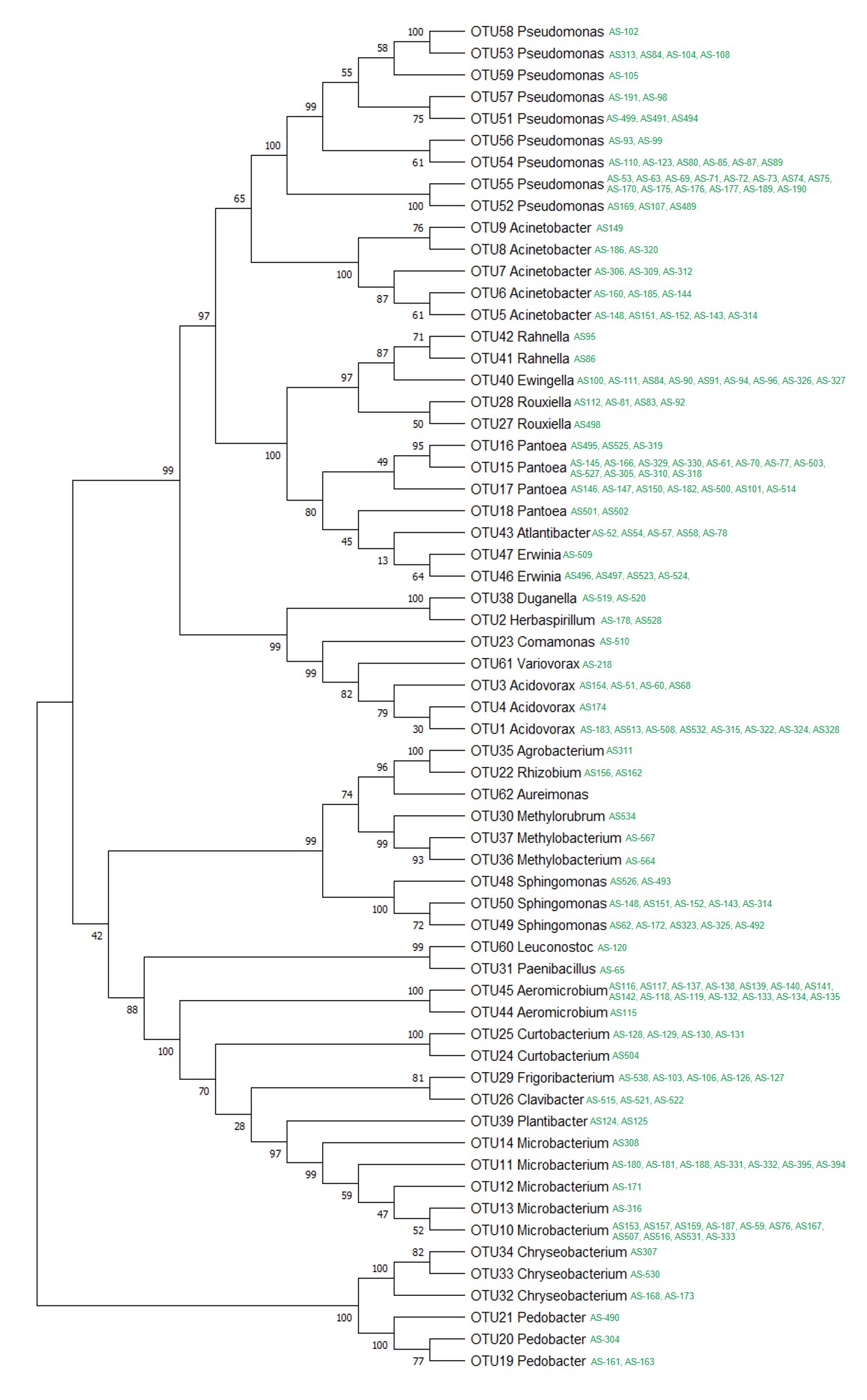

Supplement: Supplementary Figure 1 — A Maximum likelihood (ML) phylogenetic tree of the entire bacterial population based on unique operational taxonomic units (OTUs) (in black letters) and all the 201 bacterial strains cultured (strains ID in green letters). Bootstrap values are indicated above the branches. [file DataSheet_1.zip › Figure S1.JPEG]

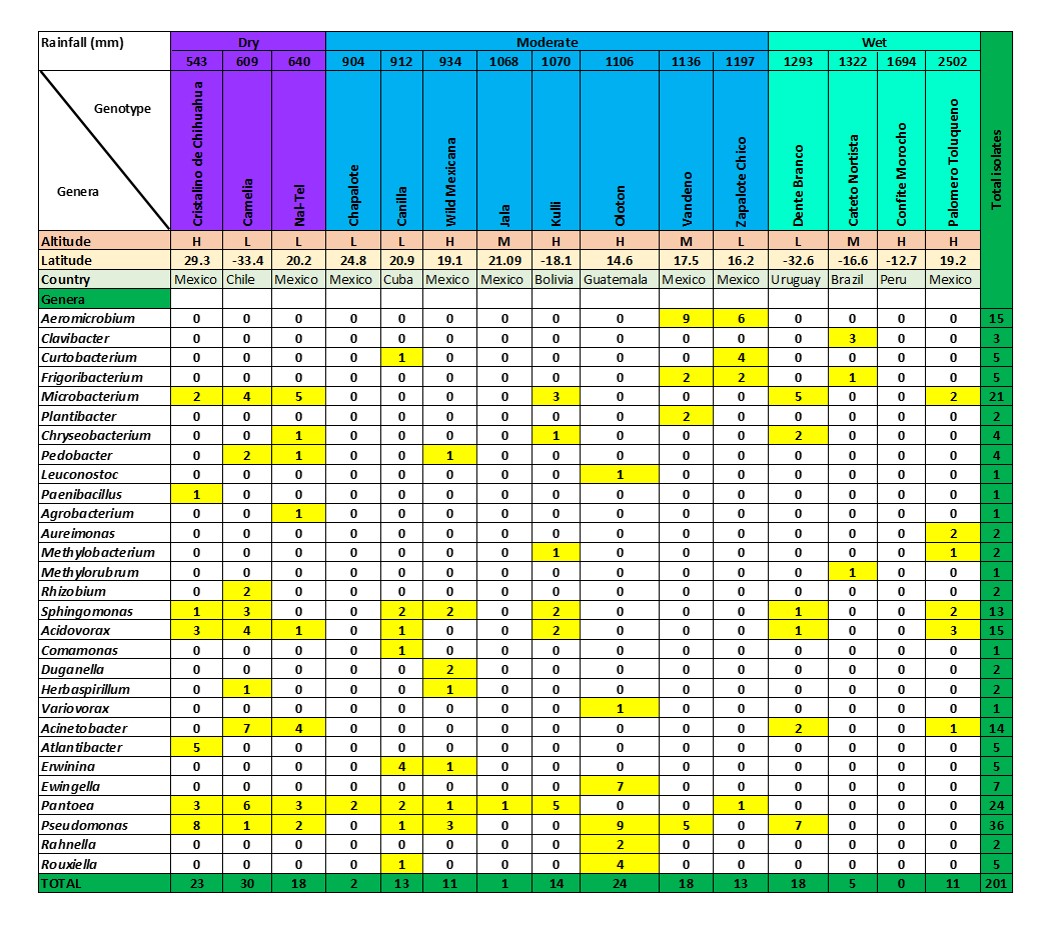

Supplement: Supplementary Figure 1 — A Maximum likelihood (ML) phylogenetic tree of the entire bacterial population based on unique operational taxonomic units (OTUs) (in black letters) and all the 201 bacterial strains cultured (strains ID in green letters). Bootstrap values are indicated above the branches. [file DataSheet_1.zip › Figure S2.JPEG]

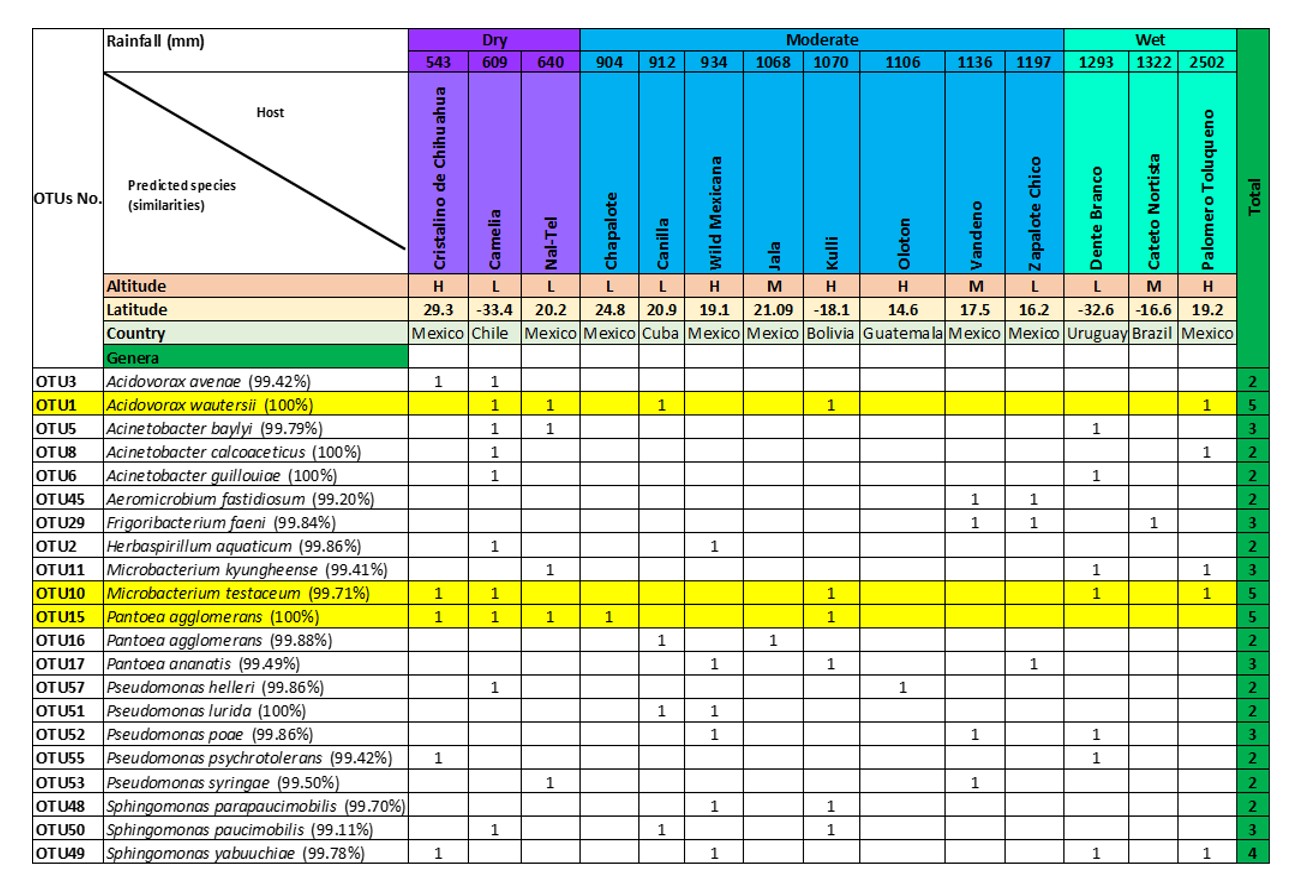

Supplement: Supplementary Figure 1 — A Maximum likelihood (ML) phylogenetic tree of the entire bacterial population based on unique operational taxonomic units (OTUs) (in black letters) and all the 201 bacterial strains cultured (strains ID in green letters). Bootstrap values are indicated above the branches. [file DataSheet_1.zip › Figure S3.JPEG]

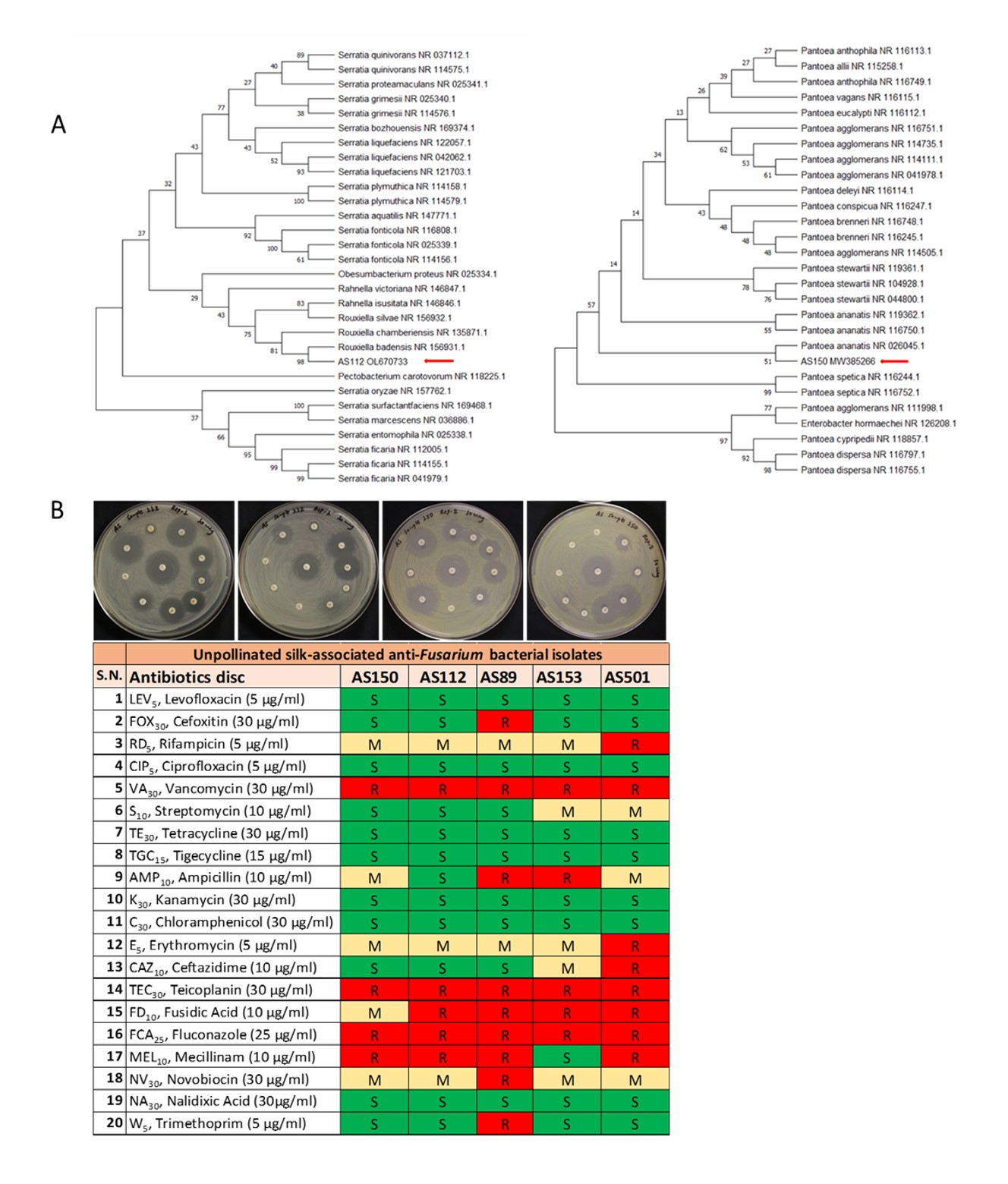

Supplement: Supplementary Figure 1 — A Maximum likelihood (ML) phylogenetic tree of the entire bacterial population based on unique operational taxonomic units (OTUs) (in black letters) and all the 201 bacterial strains cultured (strains ID in green letters). Bootstrap values are indicated above the branches. [file DataSheet_1.zip › Figure S4.JPEG]
